# MARS: leveraging allelic heterogeneity to increase power of association testing

**DOI:** 10.1186/s13059-021-02353-8

**Published:** 2021-04-30

**Authors:** Farhad Hormozdiari, Junghyun Jung, Eleazar Eskin, Jong Wha J. Joo

**Affiliations:** 1 Department of Epidemiology, Harvard T.H. Chan School of Public Health, Boston, 02115 MA USA; 2 Program in Medical and Population Genetics, Broad Institute of MIT and Harvard, Cambridge, MA USA; 3 Department of Life Science, Dongguk University-Seoul, Seoul, 04620 South Korea; 4 Department of Computer Science, University of California, Los Angeles, Los Angeles, 90095 CA USA; 5 Department of Human Genetics, University of California, Los Angeles, Los Angeles, 90095 CA USA; 6 Department of Computer Science and Engineering, Dongguk University-Seoul, Seoul, 04620 South Korea

**Keywords:** Association studies, Causal variants, Set-based association analysis

## Abstract

In standard genome-wide association studies (GWAS), the standard association test is underpowered to detect associations between loci with multiple causal variants with small effect sizes. We propose a statistical method, Model-based Association test Reflecting causal Status (MARS), that finds associations between variants in risk loci and a phenotype, considering the causal status of variants, only requiring the existing summary statistics to detect associated risk loci. Utilizing extensive simulated data and real data, we show that MARS increases the power of detecting true associated risk loci compared to previous approaches that consider multiple variants, while controlling the type I error.

**Supplementary Information:**

The online version contains supplementary material available at (10.1186/s13059-021-02353-8).

## Background

Over the past decade, genome-wide association studies (GWAS) have successfully identified many variants significantly associated with diseases and complex traits. Unfortunately, those variants only explain an extremely small proportion of phenotypic variation [1, 2] and there are many more variants with even smaller effects that we have yet to identify [1, 3–5]. Detecting all loci that harbor associated risk loci can help elucidate the biological mechanisms of diseases and complex traits. All biological follow-up studies have been performed on loci that harbor at least one significant variant. The standard association test used in GWAS examines one variant at a time to identify associated variants; we refer to this method as univariate testing.

Previous works have shown that many loci in the genome harbor more than one causal variant for a given disease or a trait [6–15]. The phenomenon is known as allelic heterogeneity, which is very common in Mendelian traits [16] and recent works have demonstrated widespread allelic heterogeneity in expression quantitative trait loci (eQTLs) and complex traits [17, 18]. The univariate test may be underpowered for a locus containing multiple causal variants with small effect sizes. Alternatively, an approach that considers the effects of multiple causal variants simultaneously may have increased statistical power to detect signals for the locus by aggregating the effects of causal variants.

In this paper, we propose a new model-based method for identifying the association between multiple variants in a locus and a trait we call Model-based Association test Reflecting causal Status (MARS). Our approach builds upon recent progress in fine-mapping approaches that attempt to identify causal variants in a locus. Causal variants are responsible for the association signal at a locus; however, each locus often contains tens to hundreds of variants tightly linked (linkage disequilibrium, LD) to the reported associated single nucleotide polymorphism (SNP). Therefore, the LD hinders the identification of causal variants at the risk locus. CAVIAR [13] is a recent fine-mapping approach that estimates the probability of each variant being causal, thus allowing an arbitrary number of causal variants by jointly modeling the association statistics at all variants. We extend the likelihood model of CAVIAR to explicitly incorporate the LD structure of data utilizing multivariate normal (MVN) distribution conditional on the causal status of the variants. MARS computes a likelihood ratio of a null model, where none of the variants are causal against an alternate model, where at least one variant is causal. Then, an efficient re-sampling approach is applied for the significance test.

Our method does not require individual-level data, which is often not provided in GWAS. MARS only requires summary statistics such as *Z*-scores and the LD of variants in a locus, which can be obtained from a reference dataset such as HapMap [19, 20] or the 1000 Genome project [21], and reports a *p*-value that indicates the significance of the association between the locus and the corresponding trait. This approach is related to set-based association tests that examine an association between a set of variants and a trait [22–24]. MARS outperforms these previous methods because its underlying model, which builds upon the model of CAVIAR, explicitly models the joint distribution of observed statistics given multiple signals of associations. Furthermore, MARS uses a significance level that corresponds to the standard GWAS significance level, thus facilitating interpretation.

When applied to several simulated data sets, we show that MARS robustly controls type I errors and has improved statistical power compared to the univariate test and widely utilized set-based association tests, a fast and flexible set-Based Association Test (fastBAT) [25], Deterministic Approximation of Posteriors (DAP-G) [26], and Sequence Kernel Association Test (SKAT) [27]. In addition, to show the performance of MARS on both eQTL studies and GWAS, we have applied MARS to representative eQTL and GWAS datasets; Genotype-Tissue Expression (GTEx) data and Northern Finland Birth Cohort (NFBC) data, respectively. Applied to the data of 44 tissues provided by the GTEx consortium [28, 29], MARS identified more eGenes, which are genes with at least one variant significantly associated with *cis* compared to those reported by the GTEx consortium in most tissues, e.g., in the Whole Blood data, MARS identified 29% more eGenes than the consortium; 57% of the extra eGenes that had only been identified by MARS, i.e., not by consortium, were reported in studies elsewhere. To demonstrate the increased power of MARS on real data, we followed a strategy of applying MARS to an older data set and validated the additionally discovered loci using current datasets that have higher statistical power because they are much larger. Applied to the 2009 NFBC data, we show that MARS effectively identifies more association loci than the univariate test and show that many of the new loci have since been discovered in recent GWAS studies.

## Results

### Overview of MARS

Causal variants are those that are responsible for the association signal at a locus. The ultimate goal of the standard association test, which examines the association between each variant and a trait, is to find causal variants; we refer to this method as univariate testing. However, multiple causal variants with small effect sizes often exist in a locus. For these cases, the univariate test may not detect those associations due to its low statistical power. Alternatively, we can examine the aggregated effect of multiple variants simultaneously on the trait to increase statistical power.

We developed a novel statistical method referred to as Model-based Association test Reflecting causal Status (MARS). MARS examines the association between a set of variants and a trait. MARS requires summary statistics estimated for variants (e.g.,*z*-score) for a locus of interest and a correlation structure, LD, between the variants, which can be readily obtained from a reference dataset. To test the association between a set of variants of a locus and a trait, MARS estimates a likelihood ratio to compute a test statistic, which is referred to as Likelihood Ratio Test (LRT) statistic; *LRT*_*stat*_. We consider the likelihood of a null model (*L*_0_) and the alternative model (*L*_1_). Note that we are computing the likelihood ratio of a null model against the alternative model, not the full model, which is the standard form of the “Likelihood ratio test” uses. The null model assumes that there is no causal variant to the trait while the alternative model assumes that there is at least one causal variant to the trait. Then, we compute the *LRT*_*stat*_ as *L*_1_/*L*_0_. Suppose that we test the association between *m* number of variants and a trait. Given the observed summary statistics, we can compute the *LRT*_*stat*_ as follows:

$${LRT_{{stat}}} = \frac{\sum_{C\in \zeta} p(S|C)p(C)}{p(S|C_{0})p(C_{0})} $$

**Fig. 1:**
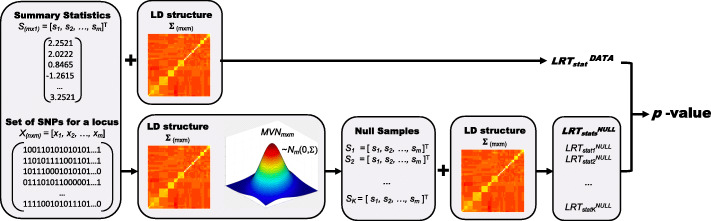
Overview of MARS. Here, we assume that we are testing an association between a locus of *m* variants and a trait. The leftmost panel shows the input of MARS; *m* number of summary statistics for the locus and an *n*×*m* matrix that contains genotypes of *m* SNPs for *n* samples. The next two panels on the bottom show the re-sampling process in which we sample the null statistics *K* times from an MVN distribution with a variance-covariance matrix of *Σ* that contains LD of the genotypes *X*. The rightmost panel shows the process by which we estimate *LRT*_*stats*_ for the null panel from which we can compute a *p*-value for the data

Here, *S*=[*s*_1_,⋯,*s*_*m*_]^**T**^ indicates summary statistics of *m* variants and *C* indicates the causal status of *m* variants. *C* is a binary vector of length *m*, where 0 indicates that a variant is non-causal and 1 indicates that a variant is causal. Specifically, *C*_0_ indicates the causal status where none of the variants are causal and *ζ* is a set that contains all possible casual statuses except for *C*_0_. Since there are *m* number of variants, there are 2^*m*^ possible causal statuses. In practice, we limited the number of allowed causal SNPs as well as the number of variants considered for a region to reduce the running time in experiments throughout the paper. We find that considering up to 3 causal variants and use 50 variants in a region to be reasonable in respect to both the running time and the accuracy in our experiments. However, a user may increase the numbers, which is amendable in high computing servers and may provide more accurate results in expense of the running time. For details, see the “Methods” section. To assess the association significance for a locus, we utilize the re-sampling approach, where we sample null statistics from a MVN distribution with a corresponding LD and estimate the *LRT*_*stats*_ for the null statistics to generate a null panel of *LRT*_*stats*_;${LRT}_{{stats}}^{NULL}$. From the null panel, we estimate the significance of ${LRT}_{{stat}}^{DATA}$ computed from the data. Figure 1 shows the basic overall process of MARS. The “Methods” section describes the details and techniques to make this process computationally feasible for big genomic data.

### MARS controls type I error while improving power in simulation studies

We demonstrate that MARS controls type I errors through simulations of null panels utilizing the GTEx data as a starting point and consider the SNPs ±1Mb around the transcription start site (TSS) of 10 genes of Whole Blood data from the GTEx consortium [28, 29]. Half of the genes are randomly selected from those reported as eGenes by the GTEx consortium and the other half are randomly selected from other genes, i.e., non-eGenes, of the GTEx consortium [28, 29]. For each gene locus, we simulate 10^7^ null summary statistics according to the generative model described in the Materials and Methods section, which uses the LD structure estimated from the genotypes of the SNPs in the locus and applies MARS to compute the *LRT*_*stats*_.

**Fig. 2:**
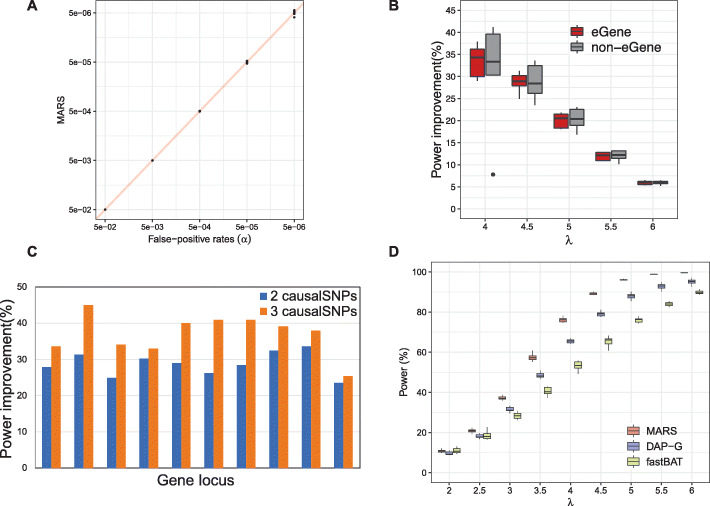
Comparison of eGenes identified by MARS and eGenes reported by GTEx consortium. **a** The plot shows that MARS controls type I error. The points represent 10 different gene loci used for the test where the five gene loci are from eGenes as reported by the GTEx consortium. **b** Box plot showing the percentage of power improvement of MARS over the univariate test for different effect sizes of two causal SNPs that exist in the data. The *X*-axis shows five different effect sizes of *λ*=4,4.5,5,5.5, and 6 used for the test. The *Y*-axis shows the percentage of power improvement. The red and black bars show the power improvement when loci not reported and reported as eGenes by the GTEx consortium are used for the test, respectively. **c** Plot comparing the percentage of power improvement of MARS over the univariate test when two and three causal variants implanted in the simulation data. Each variant has an effect size of *λ*=4.5.**d** Box plot showing the power of MARS, DAP-G, and fastBAT for different effect sizes. The *X*-axis shows the effect sizes of *λ*=2,2.5,3,3.5,4,4.5,5,5.5, and 6 used for the test. The *Y*-axis shows the power in percentages. The red, green, and blue boxplots show the power of MARS, DAP-G, and fastBAT, respectively

To show that MARS controls type I error, the false-positive rates are estimated for different thresholds of *α*=5×10^−6^ to 5×10^−2^. Half of the simulated data is used to compute a threshold of *LRT*_*stats*_ for the corresponding *α*; $\phantom {\dot {i}\!} {LRT}_{threshold^{\alpha }}$ and the other half of the simulated data is used to compute a quantile of *LRT*_*stats*_ smaller than the $\phantom {\dot {i}\!}{LRT}_{threshold^{\alpha }}$. Figure 2a shows that MARS robustly controls type I error for all examined gene loci as the false-positive rates for different gene loci are very close to the corresponding *α*=5×10^−6^ to 5×10^−2^, respectively.

To show that MARS increases the statistical power, we performed extensive simulation studies for various scenarios and compared the power of MARS with those of the univariate test. Here, we defined the univariate test as a set-based association test that uses the maximum summary statistic among SNPs in the locus we are testing (for details see the “Methods” section). The same gene loci from the previous section are used for the test and we estimate power of each gene locus for cases with two causal variants implanted with different effect sizes of *λ*=4,4.5,5,5.5, and 6. For a fair comparison of the powers between the univariate test and MARS, we utilized the standard GWAS *p*-value threshold of 5×10^−8^. We simulated 10^8^ summary statistics under the null model of no effect to generate a null panel and 10^8^ summary statistics under the alternative model of effect size *λ* for two causal variants to examine the power. To set a threshold for computing the power, we utilized the concept of the univariate test (see “The standard univariate test, fastBAT, and DAP-G” sub-section of the “Methods” section). For each null statistic, we selected the maximum *p*-value to get 10^8^ maximum *p*-value from the null panel. Then, we ordered the maximum *p*-values to get the quantile, *q*, where the maximum *p*-values corresponds to 5×10^−8^; *q* is used for setting the LRT thresholds. Then, the power is estimated as a quantile of alternative cases that show *LRT*_*stats*_ greater than the LRT threshold. The details of the whole processes of computing the threshold and estimated the power is described in the “Power estimation” sub-section of the “Methods” section. The percentage of power improvement is defined as (power of MARS – power of the univariate test)/(power of the univariate test) ×100. Figure 2b shows that MARS has increased statistical power compared to the univariate test. While the extent of power improvements differs between the gene loci as LD structures differ between loci, it is clear for all cases that the powers are improved over the univariate test. Depending on the effect size *λ* implanted in the simulated data, the power has improved from 5.2 to 41.18% in our experiments and as expected, the smaller the effect size, the better MARS performs over the univariate test. The results do not show noticeable differences between the loci of the eGenes and of the non-eGenes used for the simulations.

In addition, we examine the cases where two and three causal variants, each with an effect size of *λ*=4.5, are implanted in the simulated data. As the number of causal variants increases from two to three, MARS shows a bigger power improvement over the univariate test (Fig. 2c). The result shows that the more causal variants that exist in a locus, the better MARS performs over the univariate test.

Besides the univariate test, we compared MARS with the widely used set-based association test methods, fastBAT [25], DAP-G [26], and SKAT [27]. Due to the heavy I/O of fastBAT and DAP-G (an extended version of DAP), we used 10^5^ simulations and a threshold of 10^−5^. We computed the power of MARS, DAP-G, and fastBAT for the different effect sizes of *λ*=2,2.5,3,3.5,4,4.5,5,5.5,6 and show that MARS outperforms fastBAT and DAP-G for all cases by improving the power from 0.07 to 23.38% and from 0.6 to 10.61%, respectively, depending on the effect sizes in the experiments (Fig. 2d). In addition, we compared MARS with SKAT, optimal unified SKAT (SKAT-O) [27] and Meta-SKAT [30], which is designed for meta-analysis but applicable for a single study as well. As SKAT does not allow *z*-scores that we have used for our simulated studies as its input, we generated phenotypes with a range of effect sizes *β* for the comparison. The results show that MARS outperforms SKAT, SKAT-O, and Meta-SKAT. Additional file 1: Fig. S1 describes the experiments and results in detail.

### MARS detects novel eGenes in GTEx data

Recently, a larger number of expression quantitative trait loci (eQTLs) studies have been reported. In particular, numerous *cis*-eQTLs, which are eQTLs that map to the approximate location of their gene-of-origin, have been identified. As part of this effort, the GTEx consortium reported eGenes, which are genes with at least one *cis*-eQTL. We applied MARS to GTEx data to show that it can detect more eGenes than those reported by the GTEx consortium. Among the 44 tissues provided by the GTEx consortium, we first applied MARS to the Whole Blood data for evaluation as this data contains the largest number of samples among all tissues.

**Fig. 3:**
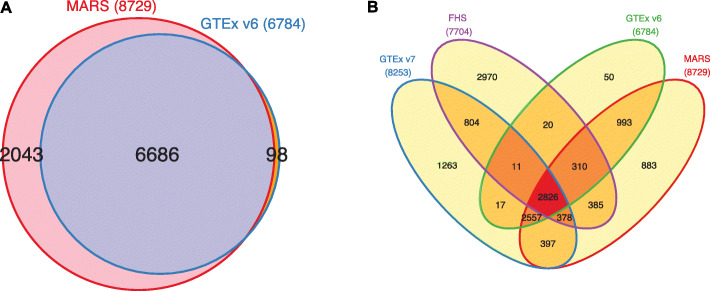
Comparison of the eGenes identified by MARS and those reported by GTEx consortium. **a** The red circle shows the eGenes identified by MARS and the blue circle shows the eGenes detected by GTEx version 6. Whole blood data was used for the analysis. **b** A Venn diagram comparing eGenes identified by GTEx version 6, GTEx version 7, FHS, and MARS. Whole blood data was used for all three studies. The blue, purple, green, and red circles show the eGenes identified by GTEx version 7, the Framingham Heart Study (FHS), GTEx version 7, and MARS, respectively. Note that MARS used data from GTEx version 6

For simulation studies and GWAS, we applied a threshold that corresponds to the *p*-values threshold of 5×10^8^ utilizing the univariate test. However, for a fair comparison of the MARS results with those reported by the GTEx consortium, a different strategy has been used. We used 10,000 simulations, which is the number of simulations used by the GTEx consortium to compute their “empirical *p*-values” and select eGenes. To identify eGenes for MARS, we set the threshold as the border of empirical *p*-values between eGenes and other genes, referred to as non-eGenes, reported by the GTEx consortium. Figure 3 is a Venn diagram that compares the identifications of eGenes by MARS and those reported by the GTEx consortium. MARS identified 2043 extra eGenes that were not reported by the consortium, while MARS missed only 98 eGenes that were reported by the consortium [28, 29]. MARS and the GTEx consortium detected 6686 eGenes in common.

To verify that the eGenes identified by MARS are true associations, we compared the extra eGenes with those reported by other studies that have larger sample sizes. Note that the results throughout the paper used data from GTEx version 6. GTEx version 7 has recently been published with more samples and improved technology in experiments. We expect more eGenes are detected in the newer version of the data as the power increases with the number of samples and etc. We compared the extra eGenes with those reported by GTEx version 7. In addition, we utilized a Whole Blood data of Framingham Heart Study (FHS) [31], which is independent of GTEx data but contains a larger sample size (5257 samples), to validate the extra eGenes. Figure 3b is a Venn diagram that compares the identifications of eGenes from four studies, GTEx version 6, GTEx version 7, FHS, and MARS. Among the 2043 extra eGenes, about 57% (1160 genes) were reported in either GTEx version 7 or FHS; 775 genes were reported as eGenes in GTEx version 7, 763 genes were reported as eGenes in FHS, and 378 genes were identified as eGenes by both GTEx version 7 and FHS. Even with the older version of the data, MARS still found more eGenes than GTEx version 7 and MARS is expected to identify even more eGenes when using data with a larger number of samples in further studies. Moreover, some of the 883 genes (Fig. 3b) that were only identified by MARS and not by GTEx version 6, GTEx version 7, or FHS, have biological evidence of being eGenes based on many studies in the literature. Variants of the *SP*140 (ENSG00000079263) gene are known to be related to multiple sclerosis (MS) [32] and chronic lymphocytic leukemia [33]. Sille et al. have demonstrated that the expression level of *SP*140 is regulated by *cis*-eQTLs in lymphoblastoid cell lines [34]. Besides, the *SP*140 protein levels are shown to be downregulated by a *cis*-acting mechanism in peripheral blood mononuclear cells (PBMCs) from MS patients [35]. *HSPB*8 (ENSG00000152137) has been recently identified as an eGene using PBMCs and the expression level of *HSPB*8 is known to be regulated by several SNPs [36]. Surfactant protein D encoded by SFTPD (ENSG00000133661) gene is known to be regulated by in a *cis*-acting manner in human blood [37], and *CD*83 (ENSG00000112149) has been recently identified as *cis*-eQTLs gene in CD19+ B lymphocyte [38]. Additionally, in Additional file 1: Fig. S2, we thoroughly analyzed 100 randomly selected genes and compared the *p*-values for MARS, the univariate test, and those reported by the GTEx consortium to show that MARS could identify more eGenes with better *p*-values. These results show that MARS is capable of identifying novel eGenes that cannot be detected using standard association test approaches. Additional file 2: Table S1 lists the 2048 extra eGenes and their identifications in GTEx version 7 and FHS.

**Fig. 4:**
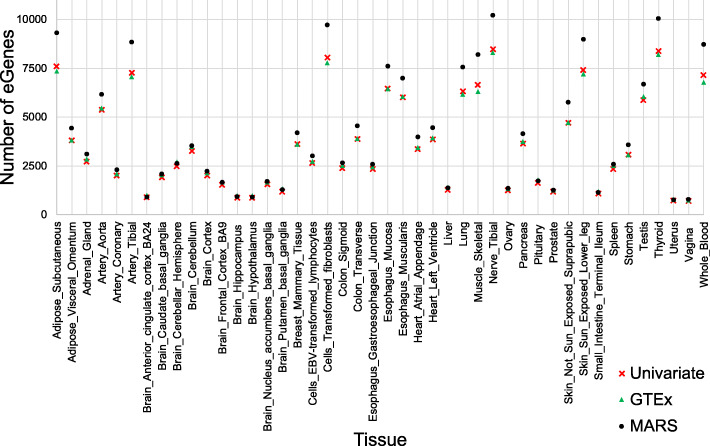
Number of eGenes identified by MARS, the univariate test, and those reported by the GTEx consortium. The *x*-axis shows the 44 tissues provided by the GTEx consortium and the *y*-axis shows the number of eGenes identified by each method. The black circle shows the number of eGenes identified by MARS, the red cross shows the number of eGenes identified by the univariate test, and the green triangle shows the number of eGenes reported by the GTEx consortium

One advantage of MARS is that once the null panel of *LRT*_*stats*_ for each gene has been established, this can be applied to the gene in any other tissues. Utilizing the null panel of *LRT*_*stats*_ estimated from the Whole Blood data of the GTEx consortium, we computed the *p*-values of the genes in all 44 tissues of GTEx using their summary statistics and LD structures. Figure 4 shows that MARS identifies comparable or more eGenes than the univariate test in addition to those reported by the GTEx consortium in all tissues. As expected, the number of eGenes identified by the univariate test and those reported by the GTEx consortium are very close to each other in all of the tissues. The numbers of genes differ between tissues due to factors such as sample size differences and we only used the common genes in each tissue and the Whole Blood data of the GTEx consortium because the null panel of *LRT*_*stats*_ was estimated for genes in the Whole Blood data. Additional file 3: Table S2 provides a comparison of eGenes identified by MARS, the univariate test, and the GTEx consortium for each tissue. In addition, we compared eGenes identified by MARS, GTEx version 6, and GTEx version 7 and provided Venn diagrams as in Fig. 3 for all tissues (Additional file 1: Fig. S3).

### MARS detects more set-based associations in GWAS

We show the effectiveness of our method on GWAS by applying MARS to the Northern Finland Birth Cohort (NFBC) data [39]. The NFBC data consist of 10 traits collected from 5327 individuals, namely triglycerides (TG), high-density lipoproteins (HDL), low-density lipoproteins (LDL), glucose (GLU), insulin (INS), body mass index (BMI), and C-reactive protein (CRP) as a measure of inflammation, systolic blood pressure (SBP), diastolic blood pressure (DBP), and height. For NFBC data, we examined 51,762 loci, where each locus is defined as ±1 Mb of TSS of genes provided by the GTEx consortium.

**Fig. 5:**
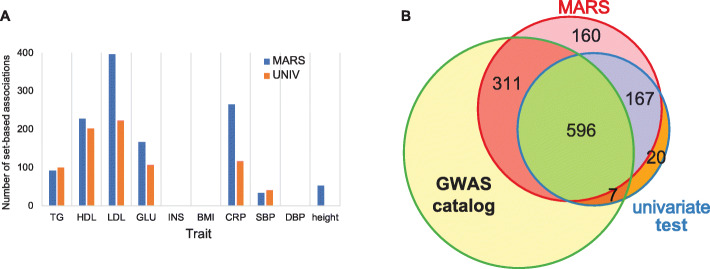
Significant associations identified by MARS and the univariate test in NFBC data. **a** Number of significant associations identified by MARS and the univariate test. The *x*-axis shows the 10 traits of NFBC data and the *y*-axis shows the number of set-based associations that will likely be associated with the traits. The blue bars show associations identified by MARS and the orange bars show associations identified in the univariate test. The 10 phenotypes are triglycerides (TG), high-density lipoproteins (HDL), low-density lipoproteins (LDL), glucose (GLU), insulin (INS), body mass index (BMI), C-reactive protein (CRP) as a measure of inflammation, systolic blood pressure (SBP), diastolic blood pressure (DBP), and height. **b** A Venn diagram showing the number of loci was found by the GWAS catalog for 10 traits. The red circle shows the number of loci identified by MARS, the blue circle shows the number of loci identified by the univariate test, and the green circle shows the number of identifications reported by the GWAS catalog

MARS requires a lot of sampling to apply the standard GWAS *p*-value threshold of 5×10^−8^. To reduce the running time, we apply the idea of importance sampling [40] on MARS for the GWAS data, which well approximates the *p*-value estimated from the original sampling approach while reducing the sampling number dramatically, from 10^8^ to 10^4^ (Additional file 1: Fig. S4). For further details, see the “Fast and space-efficient sampling for MARS” sub-section of the “Methods” section. Figure 5a shows that, for all traits, MARS identifies more or comparable loci that are likely significantly associated with the traits. A total of 471 loci were only identified by MARS and not by the univariate test. Additional file 4: Table S3 lists significantly associated loci identified by MARS and by the univariate test and Additional file 1: Fig. S5 provides Venn diagrams that compare the identifications of MARS and the univariate test. To verify those extra loci, we searched the loci from other GWAS by utilizing the GWAS catalog [41]. As a result, several variants associated with 311 loci among those 471 extra loci have been previously reported [7, 42–66]. For example, the rs6060369 locus associated with height was reported by large GWAS [49–51]. The rs1800961 locus related to HDL was previously reported by large GWAS and meta-analysis GWAS [59–61, 65, 66]. The rs6511720 locus related to LDL was discovered by several previous studies [45, 55, 56, 60, 61, 65]. A Venn diagram in Fig. 5b compares the number of loci identified by MARS and the univariate test as well as showing the number of loci for which at least one associated variant has been reported by the GWAS catalog. The list of SNPs, the corresponding loci found by previous studies, and their detailed information including PubMed id and SNP position are provided in Additional file 5: Table S4. Note that loci are defined based on the gene map of GTEx (±1Mb of TSS), so some loci may overlap (Additional file 1: Fig. S6). For the height, the univariate test found no associations while MARS found 53 associations. To verify the 53 identifications of MARS on the height, we performed the univariate test on the genetic investigation of anthropometric traits (GIANT) consortium data set [67] that contains 131,547 samples and is thus much larger than the NFBC data set and expected to have greater power on the association test. As a result, we found 5788 associations where all 53 associations that MARS found are included to show that MARS’s identifications on height are true-positive signals. These results demonstrate that MARS can efficiently identify novel associations in GWAS.

## Discussion

Great efforts have been spent on finding the hidden heritability and many studies suspect that single-level variant tests miss signals due to the small effect sizes and power problems. Approaches that examine multiple variants together may have increased statistical power to detect risk loci with small effect sizes. Moreover, interpreting Genome-wide Association Studies (GWAS) at the gene level is an important step toward understanding the molecular processes that lead to disease [68, 69]. Several statistical approaches have been proposed that test the association between a set of variants and a trait or disease status; however, they simply use naïve statistics such as the mean or sum of *χ*^2^ for statistics in the risk loci [24, 25, 70, 71].

Our method examines the association between a set of variants and a trait considering the causal status and LD between variants, utilizing the model used in a recent fine-mapping approach [13, 72]. One of the advantages of MARS is that it may be applicable to data with only summary statistics, utilizing LD estimated from a global reference dataset such as 1000 genome data [73]. Note that a special attention may be required for the data from multiancestry or human leukocyte antigen (HLA) region, for which a reference dataset may not provide an accurate LD estimate due to population stratification [73] or complex LD patterns [74]. Another advantage of our method is that once a null panel of test statistics has been established for a locus, it can be applied to the locus in other studies, only if the analyzed variants (thus, the LD structures) of the locus are the same. For example, in our GTEx analysis, the null panel statistics for genes were established only once and applied to all of the 44 tissues. This may reduce the running time significantly as most of the running burden comes from the null panel generation.

Applied to extensive simulated data sets with different effect sizes and the number of causal variants, our method shows improved power compared to previous approaches including the widely used set-based association test, fastBAT and DAP-G, while successfully controlling type I errors. Especially, when there are many causal variants with small effect sizes, our method shows superior performance to the standard univariate association test approach. Applied to Genotype-Tissue Expression (GTEx) data, our method identifies more or comparable eGenes compared to the standard univariate approach as well as those reported by the GTEx consortium in all tissues. In addition, using Whole Blood data, we show that a large portion of the eGenes only identified by MARS have been reported by other larger studies and some of them have biological evidence of being eGenes based on previous literature. Lastly, utilizing the Northern Finland Birth Cohort (NFBC) data, we show the effectiveness of our method to the GWAS in that our method effectively identifies more association loci in GWAS compared to the standard association test approach. In the experiments, we have defined each locus as a gene; however, it could be defined as any set of variants that a user wants to apply to.

We note some limitations in our work. First, MARS is computationally costly compared to the standard GWAS method as MARS tests the significance of an association based on the re-sampling approach. However, in practice, we introduce fast and space-efficient sampling techniques including importance sampling to dramatically reduce the sampling time, which closely approximates the original result, while we were able to successfully handle big eQTL data sets that contain tens of thousands of genes and GWAS data sets with thousands of samples. Second, we limited the number of causal variants in a locus up to three in the simulated studies to reduce the running time in experiments. We believe this is a reasonable assumption, as it has been reported that a relatively small number of variants exist in a region [13] and MARS showed better results in practice, utilizing real eQTL and GWAS datasets: GTEx data and NFBC data. However, this may not be a general assumption and further investigations may require for the cases with a larger number of causal variants (>3), comparing with previous methods such as DAP-G and fastBAT, which may give better results for the cases. Third, our model is based on a linear model and can only be applied to common variants, not rare variants. For data that does not follow a normal distribution, we recommend to fit the data into a normal distribution using techniques such as inverse normal transformation. Also, we assume the summary statistics are corrected for population stratification. We can extend our likelihood ratio model using CAVIAR-gene [15] instead of CAVIAR to consider the population stratification in the future study. Lastly, MARS does not utilize existing functional data; some current methods utilize functional data to detect more eGenes [75–77]. We can extend the statistical framework of MARS to utilize functional data in future work. Despite these limitations, MARS is a novel statistical method that can detect newly associated loci and increase the number of loci in follow-up studies. Through this, MARS can increase our biological understanding of diseases and complex traits.

## Methods

In this section, we assume that phenotypic values are continuous values to ease some of mathematical derivations of computing the summary statistics. MARS only require the joint distribution of summary statistics to follow multivariate normal distribution. It has been shown that for binary phenotypes the joint distribution of summary statistics follows a multivariate normal distribution [15, 78, 79]; thus, summary statistics obtained from case/control phenotypes are applicable for MARS as well.

### GWAS statistics

Consider GWAS on a quantitative trait where we genotype *n* individuals and collect a phenotype for them. Let *X*_*i*_ be a vector of length *n* with the standardized genotypic values (i.e., mean zero and variance one) of the *i*th marker that we are testing and *Y* be a vector of length *n* with the phenotypic values. We assume that the data-generation model follows the following linear additive model:

$$Y=\mu \mathbf{1_{n}}+X_{i}\beta_{i}+\mathbf{e}  $$

Here, *μ* is the mean of the phenotypic values, 1_*n*_ is a vector of *n* ones, *β*_*i*_ is their coefficients, and **e** is a vector of length *n* sampled from $\mathcal {N}(0, \sigma ^{2}\mathbf {I})$ accounting for the residual errors, where **I** is an *n*×*n* identity matrix.

Under this model, the phenotype follows a MVN with the following mean and variance:

$$ Y \sim \mathcal{N}\left(\mu\mathbf{1_{n}}+X_{i}\beta_{i}, \sigma_{e}^{2}\mathbf{I}\right)  $$

By maximizing the likelihood of the model, we can estimate *β*_*i*_ as follows:

$$ \hat{\beta}_{i} = \frac{X_{i}^{T}Y}{X_{i}^{T}X_{i}},\hat{\beta}_{i}\sim \mathcal{N}\left(\beta_{i}, \frac{\sigma_{e}^{2}}{(X_{i}^{T}X)^{-1}}\right)  $$

and the summary statistic is computed as follows:

$$ S_{i}=\frac{\hat{\beta_{i}}}{\hat{\sigma_{e}}}\sqrt{X_{i}^{T}X_{i}}, S_{i} \sim \mathcal{N}\left(\lambda_{i},1\right)  $$,

where *λ*_*i*_ is non-centrality parameter (NCP) and is equal to $\frac {\beta _{i}}{\sigma _{e}}\sqrt {X_{i}^{T}X_{i}}$. We obtained the estimated values for *μ*,**e**, and *σ*_*e*_ as $\hat {\mu }=\frac {\mu \mathbf {1_{n}}^{T}X_{i}}{n}, \hat {e}=Y-\mathbf {1_{n}}\hat {\mu }-\hat {\beta _{i}}X_{i}$, and $\hat {\sigma _{e}}=\sqrt {\frac {\hat {e}^{T}\hat {e}}{n-2}}$, respectively.

### The effect of linkage disequilibrium on the statistics

Consider the case where that the *i*th SNP is causal to a phenotype and the *j*th SNP is non-causal but in LD with the *i*th SNP. The correlation between the two variants is *r*, which is approximated by $\frac {1}{n}X_{j}^{T}X_{i}$. The effect size of the *j*th SNP is computed as follows:

$$ \hat{\beta}_{j} = \frac{X_{j}^{T}Y}{X_{j}^{T}X_{j}},\hat{\beta}_{j}\sim N\left(r\beta_{i}, \frac{\sigma_{e}^{2}}{(X_{j}^{T}X)^{-1}}\right)  $$

and the statistics for the *j*th SNP are computed as follows:

$$S_{j}=\frac{\hat{\beta_{j}}}{\hat{\sigma_{e}}}\sqrt{X_{j}^{T}X_{j}}, S_{j} \sim N\left(r\lambda_{i},1\right)  $$

We can show that the covariance between the statistics is equal to the correlation of the genotypes as follows:

$$\begin{array}{*{20}l} \texttt{Cov}(S_{i}, S_{j})=&\frac{X^{T}_{i}X_{j}}{\sqrt{X^{T}_{i}X_{i}}\sqrt{X^{T}_{j}X_{j}}}\\ =&\texttt{Cor}(X_{i}, X_{j})\equiv r_{{ij}}  \end{array} $$

Then, the joint distribution of the summary statistics for the two variants given their NCPs, *λ*_*i*_ and *λ*_*j*_, follows a multivariate normal distribution as follow:

$$\left(\left[\begin{array}{ll}S_{i}\\S_{j}\end{array}\right] {\mid}{ \left[\begin{array}{ll}\lambda_{i}\\ \lambda_{j}\end{array}\right]}\right) \sim \mathcal{N} \left(\left[\begin{array}{ll}\lambda_{i}\\ \lambda_{j} \end{array}\right], \left[\begin{array}{ll}1&r_{{ij}}\\r_{{ij}}&1\end{array}\right]\right) $$

### CAVIAR generative model

Now we consider the case with *m* SNPs. Given the true effect sizes of *m* SNPs, *Λ*=[*Λ*_1_,*Λ*_2_,⋯,*Λ*_*m*_], the summary statistics of *m* SNPs, *S*=[*S*_1_,⋯,*S*_*m*_]^*T*^, is as follows:

1 $$ (S|\Lambda) \sim \mathcal{N}(\Sigma\Lambda, \Sigma)   $$

Here, *Σ* is a correlation matrix, where *Σ*{*i,j*}=*r*_*ij*_. We utilize Fisher’s polygenic model and assume that the effect sizes follow a normal distribution. Let *C* be a binary vector of length *m* that indicates the causal status of *m* SNPs; 0 indicates that a SNP is non-causal and 1 indicates that a SNP is causal. Given the causal status *C*, we assume that the true effect size is as follows:

2 $$ (\Lambda|C) \sim \mathcal{N}\left(0,\Delta\right)   $$

where *Δ* is a diagonal matrix, where *Δ*{*i,i*}=*σ*^2^ if the *i*th SNP is causal and *Δ*{*i,j*}=*ε*, otherwise. From Eqs. (1) and (2), the likelihood of summary statistics follows a multivariate normal distribution as follows:

3 $$ (S|C) \sim \mathcal{N}\left(0,\Sigma+\Sigma\Delta\Sigma\right)   $$

Then the likelihood function is given as follows:

4 $$ \mathcal{L}(\Sigma, \Delta|S)=\frac{1}{\sqrt{(2\pi)^{m}|\Sigma+\Sigma\Delta\Sigma|}}\texttt{exp}\left(-\frac{1}{2}S^{T}(\Sigma+\Sigma\Delta\Sigma)^{-1}S\right)   $$

We use a simple model where the probability that an SNP begins causal is *γ*, which is independent from other SNPs. To compute the prior of causal status, we use the same assumptions that are widely used in fine-mapping methods, and *γ* is set to 0.01 [13, 78, 80–83]. We have shown that the choice of *γ* does not make big differences on the results (for details, see Additional file 1: Fig. S7) We assume each SNP is independent and that the probability of a SNP to be causal is equal to 0.01 [81, 82]. Therefore, we compute the prior probability as follows:

5 $$ p(C)=\prod^{m}_{i=1}\gamma^{|c_{i}|}(1-\gamma)^{1-|c_{i}|}   $$

Here, |*c*_*i*_|=1 if the *i*th SNP is causal and |*c*_*i*_|=0, otherwise. Although we use a simple prior, we can incorporate external information by using the SNP-specific prior *γ*_*i*_, which is the prior for the *i*th SNP, and then the prior probability for a more general case is $\phantom {\dot {i}\!}p(C|\gamma =[\gamma _{1}, \cdots \gamma _{m} ])=\prod ^{m}_{i=1}\gamma _{i}^{|c_{i}|}(1-\gamma _{i})^{1-|c_{i}|}$.

### Model-based Association test Reflecting causal Status (MARS)

MARS examines the association between a set of SNPs and a phenotype of interest. For the test statistic, we utilize a likelihood ratio test (LRT). We consider the likelihoods of two models: that of the null model (*L*_0_) and that of the alternative model (*L*_1_). The null model assumes that there is no causal SNP to the phenotype while the alternative model assumes that there is at least one causal SNP for the phenotype. Then, we can compute the test statistic as *LRT*_*stat*_=*L*_1_/*L*_0_. Given the observed marginal association statistics *S* and correlation matrix *Σ*, we can compute the *LRT*_*stat*_ as follows:

6 $$ {LRT_{{stat}}} = \frac{\sum_{C\in \zeta} p(S|C)p(C)}{p(S|C_{0})p(C_{0})}   $$

**Fig. 6:**
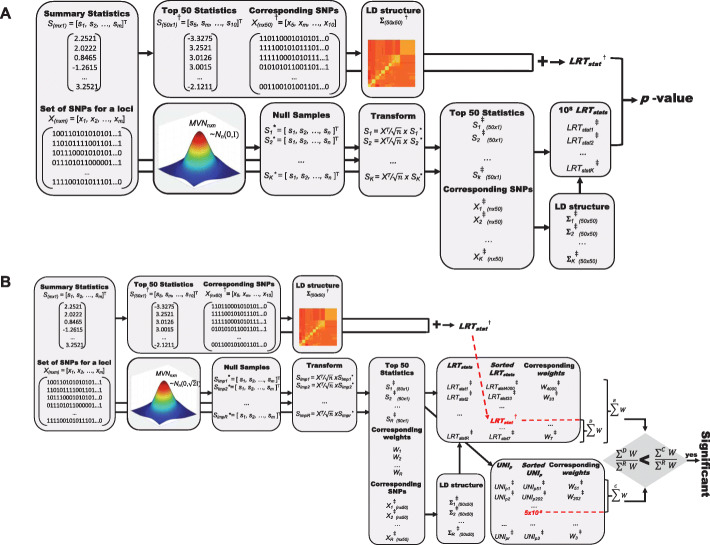
Practical implementation of MARS and fast and efficient sampling strategy for MARS. **a** To reduce the running time and space requirement, MARS uses the top 50 statistics instead of all the SNPs in its analysis. **b** For GWAS, we introduce a fast and efficient sampling strategy

Here, we can compute the prior using Eq. (5) and the likelihood using Eq. (4). Since there are *m* SNPs, there are 2^*m*^ potential causal statuses. In practice, we limit the number of allowed causal SNPs to two or three as which is consistent with reports from previous studies that a relatively small number of causal SNPs exist in a region. In addition, as the size of genes are often very large—many genes contain more than 10,000 SNPs within ±1Mb of TSS for the GTEx data—we order the SNPs by the value of its summary statistics and only used the top 50 SNPs for computing the *LRT*_*stats*_ to reduce the running time and the space. Figure 6a shows this practical implementation of MARS used for the experiments. This strategy dramatically reduces the running time while well approximating the results using all the SNPs in the loci (Additional file 1: Fig. S8) because the causal SNPs are expected to be included in the top 50 SNPs. When limiting the number of causal SNPs up to three and using care $\sum ^{3}_{i=1}\left (\begin {array}{c}50\\i\end {array}\right)$casual statuses to consider and *ζ* becomes a set that contains all the possible casual statuses with 1, 2, or 3 causal SNPs. However, depending on the available computational power and size and properties of the data, the number of possible causal variants for running MARS may increase using options in the MARS program.

### eGene detection in GTEx data

To identify an eGene, we examine the association between the gene expression levels and SNPs within ±1Mb of TSS of the gene, which can be the candidates of *cis*-eQTLs for the gene. To assess the significance of a gene, we sample summary statistics from an MVN distribution under the null hypothesis, *S*∼*N*(0,*Σ*). Here, *Σ* is a variance-covariance matrix estimated from the SNPs within ±1Mb of TSS of the gene. Based on the simulation data, we order the SNPs by values of its summary statistics and used only top 50 SNPs for computing the *LRT*_*stats*_; ${LRT}_{{stats}}^{NULL}$ using equation (6). Then, we also select the top 50 SNPs of summary statistics to compute the *LRT*_*stat*_ of the gene; ${LRT}_{{stat}}^{DATA}$, using the equation (6). The *p*-value of the gene is estimated as the quantile of ${LRT}_{{stat}}^{DATA}$ among ${LRT}_{{stats}}^{NULL}$. One of the advantages of MARS is that once the null panel, ${LRT}_{{stats}}^{NULL}$, has been estimated for a locus, the panel can be rapidly applied to the locus in any other tissues or traits to compute a *p*-value. We use the Whole Blood data, which contains the greatest number of samples among the 44 tissues, to estimate the null panels of 23,163 genes and applied the panels to all the other tissues. To compare the MARS results with GTEx’s results, we use 10^4^ simulations, the number used by GTEx Consortium to compute their “empirical *p*-values” to select eGenes. To identify eGenes for MARS, we set the threshold as the border of empirical *p*-value between eGenes and genes other than those eGenes, referred to as non-eGenes, reported by the GTEx consortium, which is differ by tissues as GTEx used the FDR approach to find their eGenes. A similar process can be applied to detect eGenes in the univariate test except using the maximum summary statistic as the test statistic instead of *LRT*_*stat*_.

### Power estimation

To show MARS increases statistical power over the univariate test, we compare the power between MARS and the univariate test. For a fair comparison, we utilized the standard GWAS *p*-value threshold of 5×10^−8^. We sampled 10^8^ number of summary statistics under the null hypothesis, *S*^*NULL*^∼*N*(0,*Σ*) and 10^8^ number of summary statistics under the alternative hypothesis, *S*^*ALT*^∼*N*(*ΣΛ*,*Σ*). Here, *Λ* is a vector of length *m*, where *m* is the number of SNPs that contain zeros except for the causal SNPs. For example, for a simulation, in which two SNPs (e.g., SNP 1 and SNP 2) with effect size *λ* are implanted in the data, *Λ* is [*λ*,*λ*,0,...,0]. We examined the power for cases with two causal or three causal SNPs implanted in the simulated data, where the causal SNPs are randomly selected for each simulation. Then, we computed the *p*-value of *S*^*NULL*^ using the univariate test, UNI *p*^*NULL*^, and found the quantile *q*, where the *p*-value equals to the standard GWAS *p*-value threshold of 5×10^−8^ as follows:

$$ q=\text{Number of (UNI}p^{NULL} < 5\times 10^{-8}) $$

We compute the *LRT*_*stats*_ of *S*^*NULL*^ as ${LRT}_{{stats}}^{NULL}$, using MARS and set the *LRT*_*stat*_ at the quantile *q* as the threshold of *LRT*_*stats*_ as ${LRT}_{{stat}}^{THR}$, which satisfies the following equation:

$$ \text{Number of} ({LRT}_{{stat}}^{NULL} > {LRT}_{{stat}}^{THR}) = q $$

Here, ${LRT}_{{stat}}^{THR}$ corresponds to the standard GWAS *p*-value threshold of 5×10^−8^. Now, we compute the *LRT*_*stats*_ of *S*^*ALT*^ as ${LRT}_{{stats}}^{ALT}$, and the power of MARS is defined as the number of ${LRT}_{{stats}}^{ALT}$ that are greater than the ${LRT}_{{stat}}^{THR}$ as follows:

$$ \text{Power of MARS} = \frac{\text{Number of} ({LRT}_{{stat}}^{ALT} > {LRT}_{{stat}}^{THR})}{10^{8}} \times 100 $$

Similarly, the power of the univariate test is defined similarly by computing the *p*-value of *S*^*ALT*^ using the univariate test; UNI *p*^*ALT*^, as follows:

$$ \text{Power of the univariate test} = \frac{\text{Number of (UNI}p^{ALT}< 5\times 10^{-8})}{10^{8}} \times 100 $$

In the power comparison of MARS, fastBAT and DAP-G, the power estimation process is the same as that described above except for that 10^5^ simulations and a threshold of 10^−5^ is used instead of 10^8^ simulations and a threshold of 5×10^−8^, respectively.

### Fast and space-efficient sampling for MARS

To access the significance of associations, MARS uses a re-sampling approach that requires a lot of sampling from MVN distribution. There are two main obstructions to this standard re-sampling approach. One is that a locus may contain many SNPs; for example, many genes in the GTEx data contain >10,000 SNPs within ±1Mb of their TSS. When the number of SNPs *m* is very large, the standard re-sampling approach, *S*∼*N*(0,*Σ*_*m*×*m*_), using the Cholesky decomposition [84] is impractical. This takes a lot of time and space as *Σ*_*m*×*m*_ itself often requires a few gigabytes of space. We can reduce the space and time complexity dramatically by utilizing the fact that *Σ*_*m*×*m*_ is a covariance matrix of *X*; *Σ*_*m*×*m*_=*X*^*T*^*X*/*n*, where *n* is the number of samples. Instead of sampling statistics from MVN with the variance-covariance matrix of *Σ*_*m*×*m*_; *S*∼*N*(0,*Σ*_*m*×*m*_), we sample statistics from MVN with the variance-covariance matrix of *I*_*n*×*n*_; *S*^∗^∼*N*(0,*I*_*n*×*n*_). This neither takes time nor space because in general *n*<<*m* and *n* is not large. Then we multiply *S*^∗^ by $X^{T}/ \sqrt {N}$ to compute the statistics $S=\frac {X^{T}}{\sqrt {N}}S^{*}$.

The other main obstruction of the standard sampling approach is that the number of sampling required to find a proper threshold for MARS may be very large. For the GTEx data, we compared the eGenes to those reported by the GTEx consortium and performed 10,000 samplings to determine the number of samples used for computing their empirical *p*-values. However, for the GWAS analysis, MARS needs to perform a lot of samplings to find a LRT threshold that corresponds to the standard GWAS *p*-value threshold of 5×10^−8^. Thus, for GWAS, we have applied importance sampling, which is an approximation method of standard sampling. The main idea of importance sampling is that it draws the sample from a distribution with thicker tails than a target distribution. Then, it uses importance weights so that the correct distribution is targeted [40]. The procedure is as follows. Instead of sampling from MVN with the variance-covariance matrix of *I*_*n*×*n*_; *S*^∗^∼*N*(0,*I*_*n*×*n*_), we sample statistics from MVN with the variance-covariance matrix of $\sqrt {2}I_{n\times n}$; $S^{*}_{{imp}} \sim N(0, \sqrt {2}I_{n\times n})$. Then, the new statistics from importance sampling become $S_{{imp}}=\frac {X^{T}}{\sqrt {N}}S^{*}_{{imp}}$. We record an additional information, referred to as the importance weight, which defined as follows:

$$ W=\frac{f(S^{*}_{}|0,I_{n\times n})}{f(S^{*}_{{imp}}|0,\sqrt{2}I_{n\times n})}  $$

Here, *f* indicates the probability density function of MVN. We repeat the process of sampling statistics $S_{{imp}}^{*}$ and computing *S*_*imp*_ and *W*, *K* times. We call each process as a run and after *K* runs we have a set of statistics $\{S_{imp_{1}}, S_{imp_{2}}, \cdots, S_{imp_{K}}\}$ and a set of weights {*W*_1_,*W*_2_,⋯,*W*_*K*_}. Then, we estimate a univariate *p*-value from each *S*_*imp*_ and compute the *p*-value threshold as the ratio of the sum of weights that have the univariate *p*-value <5×10^−8^ over the sum of all the weights as follows:

7 $$ \frac{\sum_{i}^{C}{W_{i}}}{\sum_{i}^{K}{W_{i}}}   $$

Here, *i* indicates the index of a run and *C* is a set containing the indices of runs where the univariate *p*-value <5×10^−8^. Given summary statistics of a locus, we can access the significance of the locus by computing *LRT*_*stat*_ of the summary statistics as ${LRT}_{{stat}}^{DATA}$. In addition, we compute the *K* number of *LRT*_*stats*_ for the top 50 SNPs of the *S*_*imp*_ as ${LRT}_{{stats}}^{NULL}$, as well. Then we compute the *p*-value of the locus as the ratio of the sum of weights where ${LRT}_{{stats}}^{NULL}>{LRT}_{{stats}}^{DATA}$ over the sum of all the weights as follows:

8 $$ \frac{\sum_{i}^{D}{W_{i}}}{\sum_{i}^{K}{W_{i}}}   $$

Here, *D* is a set containing indices of runs with ${LRT}_{{stats}}^{NULL}>{LRT}_{{stats}}^{DATA}$. The association is significant if the *p*-value estimated from the Eq. (8) is smaller than the *p*-value threshold estimated from the Eq. (7). Applied to 10 randomly selected genes, we find that the *p*-value estimated from the 10^4^ number of importance sampling well approximates the *p*-values estimated from the 10^8^ number of the original re-sampling (Additional file 1: Fig. S4). Utilizing the importance sampling, we can reduce the number of samplings dramatically from 10^8^ to 10^4^ in GWAS experiments. Figure 6 shows an overview of the fast and efficient association strategy for MARS (Fig. 6b).

For the GTEx data analysis, we used MARS as described in Fig. 6a, where 10^4^ number of samplings were performed and up to two causal variants were considered. In this case, MARS took approximately 3.5 min to test the significance for an average-size gene with 7522 SNPs for 338 samples in our system. Using parallel processing, we were able to run the 23,163 genes over several hours, which was approximately 3 h for sampling and computing *LRT*_*stats*_ and some extra time for pre-processing and post-processing the data. For the GWAS data analysis, we applied the fast and efficient strategy of MARS as described in Fig. 6b, where 10^4^ number of importance sampling was performed and up to two causal variants were considered. In this case, MARS took approximately 50 min to test a significance for an average-sized locus with 299 SNPs and 5326 samples in our system. Using parallel processing, we were able to run the 56,319 number of genes in approximately tow days.

### The standard univariate test, fastBAT, and DAP-G

To compare MARS with the standard approach of the set-based association test, we defined a univariate test that uses a maximum summary statistic among the SNPs in the analysis locus. In addition, the widely used set-based association tests fastBAT [25] and DAP-G [26] were used for the comparison. A Genome-wide Complex Trait Analysis (GCTA) [85] program was downloaded from the GCTA website (http://gcta.freeforums.net/thread/309/gcta-fastbat-based-association-analysis) and the “fastBAT” option was used to run GCTA-fastBAT. The DAP-G program was downloaded from the appropriate website (https://github.com/xqwen/dap/tree/master/dap_src), and summary statistics were used from the run option.

### GTEx data

The summary statistics and genotypes of 44 tissues of GTEx data version 6 were downloaded from dbGap (https://www.ncbi.nlm.nih.gov/gap); these were used to generate all results throughout this paper. The eGene list of GTEx data version 7 was downloaded from dbGap and only used to validate eGenes that had been identified by MARS applied on GTEx data version 6. In total, 23,163 gene loci selected from the Whole Blood data were used for the analysis; these contain at least 50 SNPs in their ± Mb of TSS. We generated the null panel of *LRT*_*stats*_ using Whole Blood data that contains the most samples, 338. The numbers of genes differ between tissues due to factors such as sample size differences; therefore, for eGene detection in 44 tissues, we used common gene regions in each tissue and the Whole Blood data.

### Northern Finland Birth Cohort dataset

The genotypes and 10 phenotype values of triglycerides (TG), high-density lipoproteins (HDL), low-density lipoproteins (LDL), glucose (GLU), insulin (INS), body mass index (BMI), C-reactive protein (CRP) as a measure of inflammation, systolic blood pressure (SBP), diastolic blood pressure (DBP), and height of 5326 samples from the Northern Finland Birth Cohort (NFBC) dataset were downloaded from dbGap. PLINK, a whole-genome association analysis toolset (http://zzz.bwh.harvard.edu/plink/index.shtml), was used to compute the statistics. For the set-based association test, the gene map of the GTEx data that contains 56,319 gene positions was used to define the loci for analysis. SNPs ±1 Mb around the transcription start site (TSS) of the genes were searched in the NFBC genotype data and 51,762 regions with >50 SNPs were used for the analysis. 10^4^ importance samplings were performed to generate the null panel to estimate the *p*-values of MARS and the univariate test.

### GIANT consortium height dataset

To evaluate the identifications of MARS on the NFBC height data, we performed univariate tests on the GIANT consortium height dataset, which contains 131,547 samples. [67].

## Supplementary Information

**Additional file 1** Fig. S2— MARS detects more eGenes in GTEx Whole Blood data.
Fig. S3— Venn diagrams comparing eGenes identified by MARS using GTEx v6, eGenes reported by GTEx v6, and eGenes reported by GTEx v7 for all the tissues.
Fig. S4— Compare p-values estimated from the standard sampling and those estimated from the importance sampling.
Fig. S5— Venn diagrams comparing set-based association identified by MARS and the univariate test for traits in NFBC data.
Fig. S6— Number of loci found by previous studies among the loci identified by MARS but not by the univariate test.
Fig. S7—Power of MARS on the choice of priors on gamma.
Fig. S8— Using only top 50 SNPs with bigger summary statistics well approximates the result using all the SNPs in MARS.

**Additional file 2** Extra eGenes identified by MARS but not by GTEx v6. have been reported by other studies. We used the Whole Blood data of GTEx v6 and identified 2,043 extra eGenes that were not reported by the GTEx consortium (GTEx v6). We compared those extra eGenes with a newer version of data recently published by the GTEx consortium, GTEx v7, as the newer version of the data is expected to contain more eGenes with a bigger sample size. In addition, we compared those extra eGenes with the eGenes reported by Framingham Heart Study (FHS) data that has a large number of samples, 5257 samples, using the Whole Blood data. In each column, ‘0’ indicates the gene is not identified as an eGene and ‘1’ indicates the gene is identified as an eGene.

**Additional file 3** List of eGenes identified by MARS, the univariate test, and GTEx 23,163 genes from the Whole Blood data were used for the analysis, which contain at least 50 SNPs in their +/- 1Mb of TSS. We generate null distribution of LRTstats using the Whole Blood data as which contains the maximum number of samples,338. The number of genes is different between tissues due to the sample size differences and etc, thus, for the eGene detection in 44 tissues, we use genes common in each tissue and the Whole Blood data. The first column shows the gene ID, the second column shows eGenes identified by MARS, the third column shows eGenes identified by the univariate test and last column shows the eGenes reported by GTEx consortium. In each column ‘0 ‘indicates the gene is not identified as an eGene and ‘1’ indicates the gene is identified as an eGene.

**Additional file 4** List of genes significantly associated with traits in NFBC data detected by MARS and the univariate test. The first column shows the geneID. Only genes at least one of the method, MARS or the univariate test, is significantly associated with the trait is shown. The second column and the third column show whether SNPs +/- 1Mb of TSS of the gene is significantly associated with the trait or not for MARS and the univariate test. Here, ‘1’ indicates the gene is significantly associated with the trait and ‘0’ indicates the gene is not significantly associated with the trait.

**Additional file 5** List of loci found by previous studies among the loci identified by MARS but not by the univariate test

**Additional file 6** Review history

## Data Availability

GTEx and NFBC data are available from The database of Genotypes and Phenotypes (dbGaP) under accession number phs000424.v8.p2 (https://www.ncbi.nlm.nih.gov/projects/gap/cgi-bin/study.cgi?study_id=phs000424.v8.p2) and phs000276.v2.p1 (https://www.ncbi.nlm.nih.gov/projects/gap/cgi-bin/study.cgi?study_id=phs000276.v2.p1), respectively. The FHS eQTL results are available from the NCBI ftp site (ftp://ftp.ncbi.nlm.nih.gov/eqtl/original_ submissions/FHS_eQTL/). The GIANT height data is publicly available online (https://alkesgroup.broadinstitute.org/sumstats_formatted/). The MARS R package is offered under a GPLv3 license on the GitHub repository (https://github.com/junghyunJJ/marsR) [86] and Zendo (10.5281/zenodo.4679205) [87]. Declarations

## References

[1] Yang J, Benyamin B, McEvoy BP, Gordon S, Henders AK, Nyholt DR, Madden PA, Heath AC, Martin NG, Montgomery GW, Goddard ME, Visscher PM (2010). Common SNPs explain a large proportion of the heritability for human height. Nat Genet.

[2] Wood AR, Esko T, Yang J, Vedantam S, Pers TH, Gustafsson S, Chu AY, Estrada K, Kutalik Z, Amin N (2014). Defining the role of common variation in the genomic and biological architecture of adult human height. Nat Genet.

[3] Goldstein DB (2009). Common genetic variation and human traits. N Engl J Med.

[4] Loh P-R, Bhatia G, Gusev A, Finucane HK, Bulik-Sullivan BK, Pollack SJ, de Candia TR, Lee SH, Wray NR, Kendler KS (2015). Contrasting genetic architectures of schizophrenia and other complex diseases using fast variance-components analysis. Nat Genet.

[5] Boyle EA, Li YI, Pritchard JK (2017). An expanded view of complex traits: from polygenic to omnigenic. Cell.

[6] Haiman CA, Le Marchand L, Yamamato J, Stram DO, Sheng X, Kolonel LN, Wu AH, Reich D, Henderson BE (2007). A common genetic risk factor for colorectal and prostate cancer. Nat Genet.

[7] Lango Allen H, Estrada K, Lettre G, Berndt SI, Weedon MN, Rivadeneira F, Willer CJ, Jackson AU, Vedantam S, Raychaudhuri S, Ferreira T, Wood AR, Weyant RJ, Segre AV, Speliotes EK, Wheeler E, Soranzo N, Park JH, Yang J, Gudbjartsson D, Heard-Costa NL, Randall JC, Qi L, Vernon Smith A, Magi R, Pastinen T, Liang L, Heid IM, Luan J, Thorleifsson G, Winkler TW, Goddard ME, Sin Lo K, Palmer C, Workalemahu T, Aulchenko YS, Johansson A, Zillikens MC, Feitosa MF, Esko T, Johnson T, Ketkar S, Kraft P, Mangino M, Prokopenko I, Absher D, Albrecht E, Ernst F, Glazer NL, Hayward C, Hottenga JJ, Jacobs KB, Knowles JW, Kutalik Z, Monda KL, Polasek O, Preuss M, Rayner NW, Robertson NR, Steinthorsdottir V, Tyrer JP, Voight BF, Wiklund F, Xu J, Zhao JH, Nyholt DR, Pellikka N, Perola M, Perry JR, Surakka I, Tammesoo ML, Altmaier EL, Amin N, Aspelund T, Bhangale T, Boucher G, Chasman DI, Chen C, Coin L, Cooper MN, Dixon AL, Gibson Q, Grundberg E, Hao K, Juhani Junttila M, Kaplan LM, Kettunen J, Konig IR, Kwan T, Lawrence RW, Levinson DF, Lorentzon M, McKnight B, Morris AP, Muller M, Suh Ngwa J, Purcell S, Rafelt S, Salem RM, Salvi E, Sanna S, Shi J, Sovio U, Thompson JR, Turchin MC, Vandenput L, Verlaan DJ, Vitart V, White CC, Ziegler A, Almgren P, Balmforth AJ, Campbell H, Citterio L, De Grandi A, Dominiczak A, Duan J, Elliott P, Elosua R, Eriksson JG, Freimer NB, Geus EJ, Glorioso N, Haiqing S, Hartikainen AL, Havulinna AS, Hicks AA, Hui J, Igl W, Illig T, Jula A, Kajantie E, Kilpelainen TO, Koiranen M, Kolcic I, Koskinen S, Kovacs P, Laitinen J, Liu J, Lokki ML, Marusic A, Maschio A, Meitinger T, Mulas A, Pare G, Parker AN, Peden JF, Petersmann A, Pichler I, Pietilainen KH, Pouta A, Ridderstrale M, Rotter JI, Sambrook JG, Schmidt CO, Sinisalo J, Smit JH, Stringham HM, Bragi Walters G, Widen E, Wild SH, Willemsen G, Zagato L, Zgaga L, Zitting P, Alavere H, Farrall M, McArdle WL, Nelis M, Peters MJ, Ripatti S, van Meurs JB, Aben KK, Ardlie KG, Beckmann JS, Beilby JP, Bergman RN, Bergmann S, Collins FS, Cusi D, den Heijer M, Eiriksdottir G, Gejman PV, Hall AS, Hamsten A, Huikuri HV, Iribarren C, Kahonen M, Kaprio J, Kathiresan S, Kiemeney L, Kocher T, Launer LJ, Lehtimaki T, Melander O, Mosley TH, Musk AW, Nieminen MS, O’Donnell CJ, Ohlsson C, Oostra B, Palmer LJ, Raitakari O, Ridker PM, Rioux JD, Rissanen A, Rivolta C, Schunkert H, Shuldiner AR, Siscovick DS, Stumvoll M, Tonjes A, Tuomilehto J, van Ommen GJ, Viikari J, Heath AC, Martin NG, Montgomery GW, Province MA, Kayser M, Arnold AM, Atwood LD, Boerwinkle E, Chanock SJ, Deloukas P, Gieger C, Gronberg H, Hall P, Hattersley AT, Hengstenberg C, Hoffman W, Lathrop GM, Salomaa V, Schreiber S, Uda M, Waterworth D, Wright AF, Assimes TL, Barroso I, Hofman A, Mohlke KL, Boomsma DI, Caulfield MJ, Cupples LA, Erdmann J, Fox CS, Gudnason V, Gyllensten U, Harris TB, Hayes RB, Jarvelin MR (2010). Hundreds of variants clustered in genomic loci and biological pathways affect human height. Nature.

[8] Galarneau G, Palmer CD, Sankaran VG, Orkin SH, Hirschhorn JN, Lettre G (2010). Fine-mapping at three loci known to affect fetal hemoglobin levels explains additional genetic variation. Nat Genet.

[9] Stahl EA, Raychaudhuri S, Remmers EF, Xie G, Eyre S, Thomson BP, Li Y, Kurreeman FA, Zhernakova A, Hinks A, Guiducci C, Chen R, Alfredsson L, Amos CI, Ardlie KG, Barton A, Bowes J, Brouwer E, Burtt NP, Catanese JJ, Coblyn J, Coenen MJ, Costenbader KH, Criswell LA, Crusius JB, Cui J, de Bakker PI, De Jager PL, Ding B, Emery P, Flynn E, Harrison P, Hocking LJ, Huizinga TW, Kastner DL, Ke X, Lee AT, Liu X, Martin P, Morgan AW, Padyukov L, Posthumus MD, Radstake TR, Reid DM, Seielstad M, Seldin MF, Shadick NA, Steer S, Tak PP, Thomson W, van der Helm-van Mil AH, van der Horst-Bruinsma IE, van der Schoot CE, van Riel PL, Weinblatt ME, Wilson AG, Wolbink GJ, Wordsworth BP, Wijmenga C, Karlson EW, Toes RE, de Vries N, Begovich AB, Worthington J, Siminovitch KA, Gregersen PK, Klareskog L, Plenge RM (2010). Genome-wide association study meta-analysis identifies seven new rheumatoid arthritis risk loci. Nat Genet.

[10] Trynka G, Hunt KA, Bockett NA, Romanos J, Mistry V, Szperl A, Bakker SF, Bardella MT, Bhaw-Rosun L, Castillejo G, de la Concha EG, de Almeida RC, Dias KR, van Diemen CC, Dubois PC, Duerr RH, Edkins S, Franke L, Fransen K, Gutierrez J, Heap GA, Hrdlickova B, Hunt S, Plaza Izurieta L, Izzo V, Joosten LA, Langford C, Mazzilli MC, Mein CA, Midah V, Mitrovic M, Mora B, Morelli M, Nutland S, Nunez C, Onengut-Gumuscu S, Pearce K, Platteel M, Polanco I, Potter S, Ribes-Koninckx C, Ricano-Ponce I, Rich SS, Rybak A, Santiago JL, Senapati S, Sood A, Szajewska H, Troncone R, Varade J, Wallace C, Wolters VM, Zhernakova A, Thelma BK, Cukrowska B, Urcelay E, Bilbao JR, Mearin ML, Barisani D, Barrett JC, Plagnol V, Deloukas P, Wijmenga C, van Heel DA (2011). Dense genotyping identifies and localizes multiple common and rare variant association signals in celiac disease. Nat Genet.

[11] Yang J, Ferreira T, Morris AP, Medland SE, Madden PA, Heath AC, Martin NG, Montgomery GW, Weedon MN, Loos RJ, Frayling TM, McCarthy MI, Hirschhorn JN, Goddard ME, Visscher PM (2012). Conditional and joint multiple-SNP analysis of GWAS summary statistics identifies additional variants influencing complex traits. Nat Genet.

[12] Flister MJ, Tsaih S-WW, O’Meara CC, Endres B, Hoffman MJ, Geurts AM, Dwinell MR, Lazar J, Jacob HJ, Moreno C (2013). Identifying multiple causative genes at a single GWAS locus. Genome Res.

[13] Hormozdiari F, Kostem E, Kang EY, Pasaniuc B, Eskin E (2014). Identifying causal variants at loci with multiple signals of association. Genetics.

[14] Kichaev G, Yang WY, Lindstrom S, Hormozdiari F, Eskin E, Price AL, Kraft P, Pasaniuc B (2014). Integrating functional data to prioritize causal variants in statistical fine-mapping studies. PLoS Genet.

[15] Hormozdiari F, Kichaev G, Yang WY, Pasaniuc B, Eskin E (2015). Identification of causal genes for complex traits. Bioinformatics.

[16] Hormozdiari F, Zhu A, Kichaev G, Ju CJ-T, Segrè AV, Joo JWJ, Won H, Sankararaman S, Pasaniuc B, Shifman S (2017). Widespread allelic heterogeneity in complex traits. Am J Hum Genet.

[17] Hormozdiari F, Zhu A, Kichaev G, Ju CJ, Segre AV, Joo JWJ, Won H, Sankararaman S, Pasaniuc B, Shifman S, Eskin E (2017). Widespread allelic heterogeneity in complex traits. Am J Hum Genet.

[18] Jansen R, Hottenga JJ, Nivard MG, Abdellaoui A, Laport B, de Geus EJ, Wright FA, Penninx BWJH, Boomsma DI (2017). Conditional eQTL analysis reveals allelic heterogeneity of gene expression. Hum Mol Genet.

[19] Consortium IH (2003). The international HapMap project. Nature.

[20] International HapMap Consortium (2007). A second generation human haplotype map of over 3.1 million SNPs. Nature.

[21] Genomes Project Consortium (2010). A map of human genome variation from population-scale sequencing. Nature.

[22] Li MX, Gui HS, Kwan JS, Sham PC (2011). GATES: a rapid and powerful gene-based association test using extended Simes procedure. Am J Hum Genet.

[23] Sul JH, Han B, Eskin E (2011). Increasing power of groupwise association test with likelihood ratio test. J Comput Biol.

[24] Ionita-Laza I, Lee S, Makarov V, Buxbaum JD, Lin X (2013). Sequence kernel association tests for the combined effect of rare and common variants. Am J Hum Genet.

[25] Bakshi A, Zhu Z, Vinkhuyzen AA, Hill WD, McRae AF, Visscher PM, Yang J (2016). Fast set-based association analysis using summary data from GWAS identifies novel gene loci for human complex traits. Sci Rep.

[26] Lee Y, Luca F, Pique-Regi R, Wen X. Bayesian multi-SNP genetic association analysis: Control of FDR and use of summary statistics. bioRxiv. 2018. https://doi.org/10.1101/316471. http://arxiv.org/abs/https://www.biorxiv.org/content/early/2018/05/08/316471.full.pdf.

[27] Wu MC, Lee S, Cai T, Li Y, Boehnke M, Lin X (2011). Rare-variant association testing for sequencing data with the sequence kernel association test. Am J Hum Genet.

[28] GTEx Consortium (2015). The Genotype-Tissue Expression (GTEx) pilot analysis: Multitissue gene regulation in humans. Science.

[29] Battle A, Brown CD, Engelhardt BE, Montgomery SB, Aguet F, Ardlie KG, Cummings BB, Gelfand ET, Getz G, Hadley K, Handsaker RE, Huang KH, Kashin S, Karczewski KJ, Lek M, Li X, MacArthur DG, Nedzel JL, Nguyen DT, Noble MS, Segre AV, Trowbridge CA, Tukiainen T, Abell NS, Balliu B, Barshir R, Basha O, Battle A, Bogu GK, Brown A, Brown CD, Castel SE, Chen LS, Chiang C, Conrad DF, Cox NJ, Damani FN, Davis JR, Delaneau O, Dermitzakis ET, Engelhardt BE, Eskin E, Ferreira PG, Fresard L, Gamazon ER, Garrido-Martin D, Gewirtz ADH, Gliner G, Gloudemans MJ, Guigo R, Hall IM, Han B, He Y, Hormozdiari F, Howald C, Kyung Im H, Jo B, Yong Kang E, Kim Y, Kim-Hellmuth S, Lappalainen T, Li G, Li X, Liu B, Mangul S, McCarthy MI, McDowell IC, Mohammadi P, Monlong J, Montgomery SB, Munoz-Aguirre M, Ndungu AW, Nicolae DL, Nobel AB, Oliva M, Ongen H, Palowitch JJ, Panousis N, Papasaikas P, Park Y, Parsana P, Payne AJ, Peterson CB, Quan J, Reverter F, Sabatti C, Saha A, Sammeth M, Scott AJ, Shabalin AA, Sodaei R, Stephens M, Stranger BE, Strober BJ, Sul JH, Tsang EK, Urbut S, van de Bunt M, Wang G, Wen X, Wright FA, Xi HS, Yeger-Lotem E, Zappala Z, Zaugg JB, Zhou YH, Akey JM, Bates D, Chan J, Chen LS, Claussnitzer M, Demanelis K, Diegel M, Doherty JA, Feinberg AP, Fernando MS, Halow J, Hansen KD, Haugen E, Hickey PF, Hou L, Jasmine F, Jian R, Jiang L, Johnson A, Kaul R, Kellis M, Kibriya MG, Lee K, Billy Li J, Li Q, Li X, Lin J, Lin S, Linder S, Linke C, Liu Y, Maurano MT, Molinie B, Montgomery SB, Nelson J, Neri FJ, Oliva M, Park Y, Pierce BL, Rinaldi NJ, Rizzardi LF, Sandstrom R, Skol A, Smith KS, Snyder MP, Stamatoyannopoulos J, Stranger BE, Tang H, Tsang EK, Wang L, Wang M, Van Wittenberghe N, Wu F, Zhang R, Nierras CR, Branton PA, Carithers LJ, Guan P, Moore HM, Rao A, Vaught JB, Gould SE, Lockart NC, Martin C, Struewing JP, Volpi S, Addington AM, Koester SE, Little AR, Brigham LE, Hasz R, Hunter M, Johns C, Johnson M, Kopen G, Leinweber WF, Lonsdale JT, McDonald A, Mestichelli B, Myer K, Roe B, Salvatore M, Shad S, Thomas JA, Walters G, Washington M, Wheeler J, Bridge J, Foster BA, Gillard BM, Karasik E, Kumar R, Miklos M, Moser MT, Jewell SD, Montroy RG, Rohrer DC, Valley DR, Davis DA, Mash DC, Undale AH, Smith AM, Tabor DE, Roche NV, McLean JA, Vatanian N, Robinson KL, Sobin L, Barcus ME, Valentino KM, Qi L, Hunter S, Hariharan P, Singh S, Um KS, Matose T, Tomaszewski MM, Barker LK, Mosavel M, Siminoff LA, Traino HM, Flicek P, Juettemann T, Ruffier M, Sheppard D, Taylor K, Trevanion SJ, Zerbino DR (2017). Genetic effects on gene expression across human tissues. Nature.

[30] Lee S, Teslovich TM, Boehnke M, Lin X (2013). General framework for meta-analysis of rare variants in sequencing association studies. Am J Hum Genet.

[31] Joehanes R, Zhang X, Huan T, Yao C, Ying SX, Nguyen QT, Demirkale CY, Feolo ML, Sharopova NR, Sturcke A, Schaffer AA, Heard-Costa N, Chen H, Liu PC, Wang R, Woodhouse KA, Tanriverdi K, Freedman JE, Raghavachari N, Dupuis J, Johnson AD, O’Donnell CJ, Levy D, Munson PJ (2017). Integrated genome-wide analysis of expression quantitative trait loci aids interpretation of genomic association studies. Genome Biol.

[32] Sawcer S, Hellenthal G, Pirinen M, Spencer CC, Patsopoulos NA, Moutsianas L, Dilthey A, Su Z, Freeman C, Hunt SE, Edkins S, Gray E, Booth DR, Potter SC, Goris A, Band G, Oturai AB, Strange A, Saarela J, Bellenguez C, Fontaine B, Gillman M, Hemmer B, Gwilliam R, Zipp F, Jayakumar A, Martin R, Leslie S, Hawkins S, Giannoulatou E, D’alfonso S, Blackburn H, Martinelli Boneschi F, Liddle J, Harbo HF, Perez ML, Spurkland A, Waller MJ, Mycko MP, Ricketts M, Comabella M, Hammond N, Kockum I, McCann OT, Ban M, Whittaker P, Kemppinen A, Weston P, Hawkins C, Widaa S, Zajicek J, Dronov S, Robertson N, Bumpstead SJ, Barcellos LF, Ravindrarajah R, Abraham R, Alfredsson L, Ardlie K, Aubin C, Baker A, Baker K, Baranzini SE, Bergamaschi L, Bergamaschi R, Bernstein A, Berthele A, Boggild M, Bradfield JP, Brassat D, Broadley SA, Buck D, Butzkueven H, Capra R, Carroll WM, Cavalla P, Celius EG, Cepok S, Chiavacci R, Clerget-Darpoux F, Clysters K, Comi G, Cossburn M, Cournu-Rebeix I, Cox MB, Cozen W, Cree BA, Cross AH, Cusi D, Daly MJ, Davis E, de Bakker PI, Debouverie M, D’hooghe MB, Dixon K, Dobosi R, Dubois B, Ellinghaus D, Elovaara I, Esposito F, Fontenille C, Foote S, Franke A, Galimberti D, Ghezzi A, Glessner J, Gomez R, Gout O, Graham C, Grant SF, Guerini FR, Hakonarson H, Hall P, Hamsten A, Hartung HP, Heard RN, Heath S, Hobart J, Hoshi M, Infante-Duarte C, Ingram G, Ingram W, Islam T, Jagodic M, Kabesch M, Kermode AG, Kilpatrick TJ, Kim C, Klopp N, Koivisto K, Larsson M, Lathrop M, Lechner-Scott JS, Leone MA, Leppa V, Liljedahl U, Bomfim IL, Lincoln RR, Link J, Liu J, Lorentzen AR, Lupoli S, Macciardi F, Mack T, Marriott M, Martinelli V, Mason D, McCauley JL, Mentch F, Mero IL, Mihalova T, Montalban X, Mottershead J, Myhr KM, Naldi P, Ollier W, Page A, Palotie A, Pelletier J, Piccio L, Pickersgill T, Piehl F, Pobywajlo S, Quach HL, Ramsay PP, Reunanen M, Reynolds R, Rioux JD, Rodegher M, Roesner S, Rubio JP, Ruckert IM, Salvetti M, Salvi E, Santaniello A, Schaefer CA, Schreiber S, Schulze C, Scott RJ, Sellebjerg F, Selmaj KW, Sexton D, Shen L, Simms-Acuna B, Skidmore S, Sleiman PM, Smestad C, Sørensen PS, Søndergaard HB, Stankovich J, Strange RC, Sulonen AM, Sundqvist E, Syvanen AC, Taddeo F, Taylor B, Blackwell JM, Tienari P, Bramon E, Tourbah A, Brown MA, Tronczynska E, Casas JP, Tubridy N, Corvin A, Vickery J, Jankowski J, Villoslada P, Markus HS, Wang K, Mathew CG, Wason J, Palmer CN, Wichmann HE, Plomin R, Willoughby E, Rautanen A, Winkelmann J, Wittig M, Trembath RC, Yaouanq J, Viswanathan AC, Zhang H, Wood NW, Zuvich R, Deloukas P, Langford C, Duncanson A, Oksenberg JR, Pericak-Vance MA, Haines JL, Olsson T, Hillert J, Ivinson AJ, De Jager PL, Peltonen L, Stewart GJ, Hafler DA, Hauser SL, McVean G, Donnelly P, Compston A (2011). Genetic risk and a primary role for cell-mediated immune mechanisms in multiple sclerosis. Nature.

[33] Speedy HE, Di Bernardo MC, Sava GP, Dyer MJ, Holroyd A, Wang Y, Sunter NJ, Mansouri L, Juliusson G, Smedby KE, Roos G, Jayne S, Majid A, Dearden C, Hall AG, Mainou-Fowler T, Jackson GH, Summerfield G, Harris RJ, Pettitt AR, Allsup DJ, Bailey JR, Pratt G, Pepper C, Fegan C, Rosenquist R, Catovsky D, Allan JM, Houlston RS (2014). A genome-wide association study identifies multiple susceptibility loci for chronic lymphocytic leukemia. Nat Genet.

[34] Sille FC, Thomas R, Smith MT, Conde L, Skibola CF (2012). Post-GWAS functional characterization of susceptibility variants for chronic lymphocytic leukemia. PLoS ONE.

[35] Matesanz F, Potenciano V, Fedetz M, Ramos-Mozo P, Abad-Grau M, Karaky M, Barrionuevo C, Izquierdo G, Ruiz-Pena JL, Garcia-Sanchez MI, Lucas M, Fernandez O, Leyva L, Otaegui D, Munoz-Culla M, Olascoaga J, Vandenbroeck K, Alloza I, Astobiza I, Antiguedad A, Villar LM, Alvarez-Cermeno JC, Malhotra S, Comabella M, Montalban X, Saiz A, Blanco Y, Arroyo R, Varade J, Urcelay E, Alcina A (2015). A functional variant that affects exon-skipping and protein expression of SP140 as genetic mechanism predisposing to multiple sclerosis. Hum Mol Genet.

[36] He P, Xia W, Wang L, Wu J, Guo YF, Zeng KQ, Wang MJ, Bing PF, Xie FF, Lu X, Zhang YH, Lei SF, Deng FY (2018). Identification of expression quantitative trait loci (eQTLs) in human peripheral blood mononuclear cells (PBMCs) and shared with liver and brain. J Cell Biochem.

[37] Sun W, Kechris K, Jacobson S, Drummond MB, Hawkins GA, Yang J, Chen TH, Quibrera PM, Anderson W, Barr RG, Basta PV, Bleecker ER, Beaty T, Casaburi R, Castaldi P, Cho MH, Comellas A, Crapo JD, Criner G, Demeo D, Christenson SA, Couper DJ, Curtis JL, Doerschuk CM, Freeman CM, Gouskova NA, Han MK, Hanania NA, Hansel NN, Hersh CP, Hoffman EA, Kaner RJ, Kanner RE, Kleerup EC, Lutz S, Martinez FJ, Meyers DA, Peters SP, Regan EA, Rennard SI, Scholand MB, Silverman EK, Woodruff PG, O’Neal WK, Bowler RP (2016). Common genetic polymorphisms influence blood biomarker measurements in COPD. PLoS Genet.

[38] Thalayasingam N, Nair N, Skelton AJ, Massey J, Anderson AE, Clark AD, Diboll J, Lendrem DW, Reynard LN, Cordell HJ, Eyre S, Isaacs JD, Barton A, Pratt AG (2018). CD4+ and B lymphocyte expression quantitative traits at rheumatoid arthritis risk loci in patients with untreated early arthritis: implications for causal gene identification. Arthritis Rheumatol.

[39] Sabatti C, Service SK, Hartikainen A-LL, Pouta A, Ripatti S, Brodsky J, Jones CG, Zaitlen NA, Varilo T, Kaakinen M, Sovio U, Ruokonen A, Laitinen J, Jakkula E, Coin L, Hoggart C, Collins A, Turunen H, Gabriel S, Elliot P, McCarthy MI, Daly MJ, Järvelin M-RR, Freimer NB, Peltonen L (2009). Genome-wide association analysis of metabolic traits in a birth cohort from a founder population. Nat Genet.

[40] Wasserman L (2004). All of Statistics: A Concise Course in Statistical Inference.

[41] MacArthur J, Bowler E, Cerezo M, Gil L, Hall P, Hastings E, Junkins H, McMahon A, Milano A, Morales J, Pendlington ZM, Welter D, Burdett T, Hindorff L, Flicek P, Cunningham F, Parkinson H (2017). The new NHGRI-EBI Catalog of published genome-wide association studies (GWAS Catalog). Nucleic Acids Res.

[42] Lu X, Huang J, Mo Z, He J, Wang L, Yang X, Tan A, Chen S, Chen J, Gu CC, Chen J, Li Y, Zhao L, Li H, Hao Y, Li J, Hixson JE, Li Y, Cheng M, Liu X, Cao J, Liu F, Huang C, Shen C, Shen J, Yu L, Xu L, Mu J, Wu X, Ji X, Guo D, Zhou Z, Yang Z, Wang R, Yang J, Yan W, Peng X, Gu D (2016). Genetic susceptibility to lipid levels and lipid change over time and risk of incident hyperlipidemia in Chinese populations. Circ Cardiovasc Genet.

[43] Coram MA, Duan Q, Hoffmann TJ, Thornton T, Knowles JW, Johnson NA, Ochs-Balcom HM, Donlon TA, Martin LW, Eaton CB, Robinson JG, Risch NJ, Zhu X, Kooperberg C, Li Y, Reiner AP, Tang H (2013). Genome-wide characterization of shared and distinct genetic components that influence blood lipid levels in ethnically diverse human populations. Am J Hum Genet.

[44] Mora S, Ridker PM (2006). Justification for the Use of Statins in Primary Prevention: an Intervention Trial Evaluating Rosuvastatin (JUPITER)?can C-reactive protein be used to target statin therapy in primary prevention?. Am J Cardiol.

[45] Kathiresan S, Melander O, Guiducci C, Surti A, Burtt NP, Rieder MJ, Cooper GM, Roos C, Voight BF, Havulinna AS, Wahlstrand B, Hedner T, Corella D, Tai ES, Ordovas JM, Berglund G, Vartiainen E, Jousilahti P, Hedblad B, Taskinen MR, Newton-Cheh C, Salomaa V, Peltonen L, Groop L, Altshuler DM, Orho-Melander M (2008). Six new loci associated with blood low-density lipoprotein cholesterol, high-density lipoprotein cholesterol or triglycerides in humans. Nat Genet.

[46] Soranzo N, Rivadeneira F, Chinappen-Horsley U, Malkina I, Richards JB, Hammond N, Stolk L, Nica A, Inouye M, Hofman A, Stephens J, Wheeler E, Arp P, Gwilliam R, Jhamai PM, Potter S, Chaney A, Ghori MJ, Ravindrarajah R, Ermakov S, Estrada K, Pols HA, Williams FM, McArdle WL, van Meurs JB, Loos RJ, Dermitzakis ET, Ahmadi KR, Hart DJ, Ouwehand WH, Wareham NJ, Barroso I, Sandhu MS, Strachan DP, Livshits G, Spector TD, Uitterlinden AG, Deloukas P (2009). Meta-analysis of genome-wide scans for human adult stature identifies novel Loci and associations with measures of skeletal frame size. PLoS Genet.

[47] Gudbjartsson DF, Walters GB, Thorleifsson G, Stefansson H, Halldorsson BV, Zusmanovich P, Sulem P, Thorlacius S, Gylfason A, Steinberg S, Helgadottir A, Ingason A, Steinthorsdottir V, Olafsdottir EJ, Olafsdottir GH, Jonsson T, Borch-Johnsen K, Hansen T, Andersen G, Jorgensen T, Pedersen O, Aben KK, Witjes JA, Swinkels DW, den Heijer M, Franke B, Verbeek AL, Becker DM, Yanek LR, Becker LC, Tryggvadottir L, Rafnar T, Gulcher J, Kiemeney LA, Kong A, Thorsteinsdottir U, Stefansson K (2008). Many sequence variants affecting diversity of adult human height. Nat Genet.

[48] Weedon MN, Lango H, Lindgren CM, Wallace C, Evans DM, Mangino M, Freathy RM, Perry JR, Stevens S, Hall AS, Samani NJ, Shields B, Prokopenko I, Farrall M, Dominiczak A, Johnson T, Bergmann S, Beckmann JS, Vollenweider P, Waterworth DM, Mooser V, Palmer CN, Morris AD, Ouwehand WH, Zhao JH, Li S, Loos RJ, Barroso I, Deloukas P, Sandhu MS, Wheeler E, Soranzo N, Inouye M, Wareham NJ, Caulfield M, Munroe PB, Hattersley AT, McCarthy MI, Frayling TM (2008). Genome-wide association analysis identifies 20 loci that influence adult height. Nat Genet.

[49] Sanna S, Jackson AU, Nagaraja R, Willer CJ, Chen WM, Bonnycastle LL, Shen H, Timpson N, Lettre G, Usala G, Chines PS, Stringham HM, Scott LJ, Dei M, Lai S, Albai G, Crisponi L, Naitza S, Doheny KF, Pugh EW, Ben-Shlomo Y, Ebrahim S, Lawlor DA, Bergman RN, Watanabe RM, Uda M, Tuomilehto J, Coresh J, Hirschhorn JN, Shuldiner AR, Schlessinger D, Collins FS, Davey Smith G, Boerwinkle E, Cao A, Boehnke M, Abecasis GR, Mohlke KL (2008). Common variants in the GDF5-UQCC region are associated with variation in human height. Nat Genet.

[50] Lettre G, Jackson AU, Gieger C, Schumacher FR, Berndt SI, Sanna S, Eyheramendy S, Voight BF, Butler JL, Guiducci C, Illig T, Hackett R, Heid IM, Jacobs KB, Lyssenko V, Uda M, Boehnke M, Chanock SJ, Groop LC, Hu FB, Isomaa B, Kraft P, Peltonen L, Salomaa V, Schlessinger D, Hunter DJ, Hayes RB, Abecasis GR, Wichmann HE, Mohlke KL, Hirschhorn JN (2008). Identification of ten loci associated with height highlights new biological pathways in human growth. Nat Genet.

[51] He M, Xu M, Zhang B, Liang J, Chen P, Lee JY, Johnson TA, Li H, Yang X, Dai J, Liang L, Gui L, Qi Q, Huang J, Li Y, Adair LS, Aung T, Cai Q, Cheng CY, Cho MC, Cho YS, Chu M, Cui B, Gao YT, Go MJ, Gu D, Gu W, Guo H, Hao Y, Hong J, Hu Z, Hu Y, Huang J, Hwang JY, Ikram MK, Jin G, Kang DH, Khor CC, Kim BJ, Kim HT, Kubo M, Lee J, Lee J, Lee NR, Li R, Li J, Liu J, Longe J, Lu W, Lu X, Miao X, Okada Y, Ong RT, Qiu G, Seielstad M, Sim X, Song H, Takeuchi F, Tanaka T, Taylor PR, Wang L, Wang W, Wang Y, Wu C, Wu Y, Xiang YB, Yamamoto K, Yang H, Liao M, Yokota M, Young T, Zhang X, Kato N, Wang QK, Zheng W, Hu FB, Lin D, Shen H, Teo YY, Mo Z, Wong TY, Lin X, Mohlke KL, Ning G, Tsunoda T, Han BG, Shu XO, Tai ES, Wu T, Qi L (2015). Meta-analysis of genome-wide association studies of adult height in East Asians identifies 17 novel loci. Hum Mol Genet.

[52] Wood AR, Esko T, Yang J, Vedantam S, Pers TH, Gustafsson S, Chu AY, Estrada K, Luan J, Kutalik Z, Amin N, Buchkovich ML, Croteau-Chonka DC, Day FR, Duan Y, Fall T, Fehrmann R, Ferreira T, Jackson AU, Karjalainen J, Lo KS, Locke AE, Magi R, Mihailov E, Porcu E, Randall JC, Scherag A, Vinkhuyzen AA, Westra HJ, Winkler TW, Workalemahu T, Zhao JH, Absher D, Albrecht E, Anderson D, Baron J, Beekman M, Demirkan A, Ehret GB, Feenstra B, Feitosa MF, Fischer K, Fraser RM, Goel A, Gong J, Justice AE, Kanoni S, Kleber ME, Kristiansson K, Lim U, Lotay V, Lui JC, Mangino M, Mateo Leach I, Medina-Gomez C, Nalls MA, Nyholt DR, Palmer CD, Pasko D, Pechlivanis S, Prokopenko I, Ried JS, Ripke S, Shungin D, Stancakova A, Strawbridge RJ, Sung YJ, Tanaka T, Teumer A, Trompet S, van der Laan SW, van Setten J, Van Vliet-Ostaptchouk JV, Wang Z, Yengo L, Zhang W, Afzal U, Arnlov J, Arscott GM, Bandinelli S, Barrett A, Bellis C, Bennett AJ, Berne C, Bluher M, Bolton JL, Bottcher Y, Boyd HA, Bruinenberg M, Buckley BM, Buyske S, Caspersen IH, Chines PS, Clarke R, Claudi-Boehm S, Cooper M, Daw EW, De Jong PA, Deelen J, Delgado G, Denny JC, Dhonukshe-Rutten R, Dimitriou M, Doney AS, Dorr M, Eklund N, Eury E, Folkersen L, Garcia ME, Geller F, Giedraitis V, Go AS, Grallert H, Grammer TB, Grassler J, Gronberg H, de Groot LC, Groves CJ, Haessler J, Hall P, Haller T, Hallmans G, Hannemann A, Hartman CA, Hassinen M, Hayward C, Heard-Costa NL, Helmer Q, Hemani G, Henders AK, Hillege HL, Hlatky MA, Hoffmann W, Hoffmann P, Holmen O, Houwing-Duistermaat JJ, Illig T, Isaacs A, James AL, Jeff J, Johansen B, Johansson A, Jolley J, Juliusdottir T, Junttila J, Kho AN, Kinnunen L, Klopp N, Kocher T, Kratzer W, Lichtner P, Lind L, Lindstrom J, Lobbens S, Lorentzon M, Lu Y, Lyssenko V, Magnusson PK, Mahajan A, Maillard M, McArdle WL, McKenzie CA, McLachlan S, McLaren PJ, Menni C, Merger S, Milani L, Moayyeri A, Monda KL, Morken MA, Muller G, Muller-Nurasyid M, Musk AW, Narisu N, Nauck M, Nolte IM, Nothen MM, Oozageer L, Pilz S, Rayner NW, Renstrom F, Robertson NR, Rose LM, Roussel R, Sanna S, Scharnagl H, Scholtens S, Schumacher FR, Schunkert H, Scott RA, Sehmi J, Seufferlein T, Shi J, Silventoinen K, Smit JH, Smith AV, Smolonska J, Stanton AV, Stirrups K, Stott DJ, Stringham HM, Sundstrom J, Swertz MA, Syvanen AC, Tayo BO, Thorleifsson G, Tyrer JP, van Dijk S, van Schoor NM, van der Velde N, van Heemst D, van Oort FV, Vermeulen SH, Verweij N, Vonk JM, Waite LL, Waldenberger M, Wennauer R, Wilkens LR, Willenborg C, Wilsgaard T, Wojczynski MK, Wong A, Wright AF, Zhang Q, Arveiler D (2014). Defining the role of common variation in the genomic and biological architecture of adult human height. Nat Genet.

[53] Berndt SI, Gustafsson S, Magi R, Ganna A, Wheeler E, Feitosa MF, Justice AE, Monda KL, Croteau-Chonka DC, Day FR, Esko T, Fall T, Ferreira T, Gentilini D, Jackson AU, Luan J, Randall JC, Vedantam S, Willer CJ, Winkler TW, Wood AR, Workalemahu T, Hu YJ, Lee SH, Liang L, Lin DY, Min JL, Neale BM, Thorleifsson G, Yang J, Albrecht E, Amin N, Bragg-Gresham JL, Cadby G, den Heijer M, Eklund N, Fischer K, Goel A, Hottenga JJ, Huffman JE, Jarick I, Johansson A, Johnson T, Kanoni S, Kleber ME, Konig IR, Kristiansson K, Kutalik Z, Lamina C, Lecoeur C, Li G, Mangino M, McArdle WL, Medina-Gomez C, Muller-Nurasyid M, Ngwa JS, Nolte IM, Paternoster L, Pechlivanis S, Perola M, Peters MJ, Preuss M, Rose LM, Shi J, Shungin D, Smith AV, Strawbridge RJ, Surakka I, Teumer A, Trip MD, Tyrer J, Van Vliet-Ostaptchouk JV, Vandenput L, Waite LL, Zhao JH, Absher D, Asselbergs FW, Atalay M, Attwood AP, Balmforth AJ, Basart H, Beilby J, Bonnycastle LL, Brambilla P, Bruinenberg M, Campbell H, Chasman DI, Chines PS, Collins FS, Connell JM, Cookson WO, de Faire U, de Vegt F, Dei M, Dimitriou M, Edkins S, Estrada K, Evans DM, Farrall M, Ferrario MM, Ferrieres J, Franke L, Frau F, Gejman PV, Grallert H, Gronberg H, Gudnason V, Hall AS, Hall P, Hartikainen AL, Hayward C, Heard-Costa NL, Heath AC, Hebebrand J, Homuth G, Hu FB, Hunt SE, Hypponen E, Iribarren C, Jacobs KB, Jansson JO, Jula A, Kahonen M, Kathiresan S, Kee F, Khaw KT, Kivimaki M, Koenig W, Kraja AT, Kumari M, Kuulasmaa K, Kuusisto J, Laitinen JH, Lakka TA, Langenberg C, Launer LJ, Lind L, Lindstrom J, Liu J, Liuzzi A, Lokki ML, Lorentzon M, Madden PA, Magnusson PK, Manunta P, Marek D, Marz W, Mateo Leach I, McKnight B, Medland SE, Mihailov E, Milani L, Montgomery GW, Mooser V, Muhleisen TW, Munroe PB, Musk AW, Narisu N, Navis G, Nicholson G, Nohr EA, Ong KK, Oostra BA, Palmer CN, Palotie A, Peden JF, Pedersen N, Peters A, Polasek O, Pouta A, Pramstaller PP, Prokopenko I, Putter C, Radhakrishnan A, Raitakari O, Rendon A, Rivadeneira F, Rudan I, Saaristo TE, Sambrook JG, Sanders AR, Sanna S, Saramies J, Schipf S, Schreiber S, Schunkert H, Shin SY, Signorini S, Sinisalo J, Skrobek B, Soranzo N, Stan?akova A, Stark K, Stephens JC, Stirrups K, Stolk RP, Stumvoll M, Swift AJ, Theodoraki EV, Thorand B, Tregouet DA, Tremoli E, Van der Klauw MM, van Meurs JB, Vermeulen SH, Viikari J, Virtamo J, Vitart V, Waeber G, Wang Z, Widen E, Wild SH, Willemsen G, Winkelmann BR, Witteman JC, Wolffenbuttel BH, Wong A, Wright AF, Zillikens MC, Amouyel P, Boehm BO, Boerwinkle E, Boomsma DI. Genome-wide meta-analysis identifies 11 new loci for anthropometric traits and provides insights into genetic architecture. Nat Genet. 2013; 45(5):501–12. 10.1038/ng.2606 PMC3973018 23563607

[54] Kanai M, Akiyama M, Takahashi A, Matoba N, Momozawa Y, Ikeda M, Iwata N, Ikegawa S, Hirata M, Matsuda K, Kubo M, Okada Y, Kamatani Y (2018). Genetic analysis of quantitative traits in the Japanese population links cell types to complex human diseases. Nat Genet.

[55] Lettre G, Palmer CD, Young T, Ejebe KG, Allayee H, Benjamin EJ, Bennett F, Bowden DW, Chakravarti A, Dreisbach A, Farlow DN, Folsom AR, Fornage M, Forrester T, Fox E, Haiman CA, Hartiala J, Harris TB, Hazen SL, Heckbert SR, Henderson BE, Hirschhorn JN, Keating BJ, Kritchevsky SB, Larkin E, Li M, Rudock ME, McKenzie CA, Meigs JB, Meng YA, Mosley TH, Newman AB, Newton-Cheh CH, Paltoo DN, Papanicolaou GJ, Patterson N, Post WS, Psaty BM, Qasim AN, Qu L, Rader DJ, Redline S, Reilly MP, Reiner AP, Rich SS, Rotter JI, Liu Y, Shrader P, Siscovick DS, Tang WH, Taylor HA, Tracy RP, Vasan RS, Waters KM, Wilks R, Wilson JG, Fabsitz RR, Gabriel SB, Kathiresan S, Boerwinkle E (2011). Genome-wide association study of coronary heart disease and its risk factors in 8,090 African Americans: the NHLBI CARe Project. PLoS Genet.

[56] Willer CJ, Sanna S, Jackson AU, Scuteri A, Bonnycastle LL, Clarke R, Heath SC, Timpson NJ, Najjar SS, Stringham HM, Strait J, Duren WL, Maschio A, Busonero F, Mulas A, Albai G, Swift AJ, Morken MA, Narisu N, Bennett D, Parish S, Shen H, Galan P, Meneton P, Hercberg S, Zelenika D, Chen WM, Li Y, Scott LJ, Scheet PA, Sundvall J, Watanabe RM, Nagaraja R, Ebrahim S, Lawlor DA, Ben-Shlomo Y, Davey-Smith G, Shuldiner AR, Collins R, Bergman RN, Uda M, Tuomilehto J, Cao A, Collins FS, Lakatta E, Lathrop GM, Boehnke M, Schlessinger D, Mohlke KL, Abecasis GR (2008). Newly identified loci that influence lipid concentrations and risk of coronary artery disease. Nat Genet.

[57] Aulchenko YS, Ripatti S, Lindqvist I, Boomsma D, Heid IM, Pramstaller PP, Penninx BW, Janssens AC, Wilson JF, Spector T, Martin NG, Pedersen NL, Kyvik KO, Kaprio J, Hofman A, Freimer NB, Jarvelin MR, Gyllensten U, Campbell H, Rudan I, Johansson A, Marroni F, Hayward C, Vitart V, Jonasson I, Pattaro C, Wright A, Hastie N, Pichler I, Hicks AA, Falchi M, Willemsen G, Hottenga JJ, de Geus EJ, Montgomery GW, Whitfield J, Magnusson P, Saharinen J, Perola M, Silander K, Isaacs A, Sijbrands EJ, Uitterlinden AG, Witteman JC, Oostra BA, Elliott P, Ruokonen A, Sabatti C, Gieger C, Meitinger T, Kronenberg F, Doring A, Wichmann HE, Smit JH, McCarthy MI, van Duijn CM, Peltonen L, Aulchenko YS, Ripatti S, Lindqvist I, Boomsma D, Heid IM, Pramstaller PP, Penninx BW, Janssens AC, Wilson JF, Spector T, Martin NG, Pedersen NL, Kyvik KO, Kaprio J, Hofman A, Freimer NB, Jarvelin MR, Gyllensten U, Campbell H, Rudan I, Johansson A, Marroni F, Hayward C, Vitart V, Jonasson I, Pattaro C, Wright A, Hastie N, Pichler I, Hicks AA, Falchi M, Willemsen G, Hottenga JJ, de Geus EJ, Montgomery GW, Whitfield J, Magnusson P, Saharinen J, Perola M, Silander K, Isaacs A, Sijbrands EJ, Uitterlinden AG, Witteman JC, Oostra BA, Elliott P, Ruokonen A, Sabatti C, Gieger C, Meitinger T, Kronenberg F, Doring A, Wichmann HE, Smit JH, McCarthy MI, van Duijn CM, Peltonen L (2009). Loci influencing lipid levels and coronary heart disease risk in 16 European population cohorts. Nat Genet.

[58] Davis JP, Huyghe JR, Locke AE, Jackson AU, Sim X, Stringham HM, Teslovich TM, Welch RP, Fuchsberger C, Narisu N, Chines PS, Kangas AJ, Soininen P, Ala-Korpela M, Kuusisto J, Collins FS, Laakso M, Boehnke M, Mohlke KL (2017). Common, low-frequency, and rare genetic variants associated with lipoprotein subclasses and triglyceride measures in Finnish men from the METSIM study. PLoS Genet.

[59] Surakka I, Horikoshi M, Magi R, Sarin AP, Mahajan A, Lagou V, Marullo L, Ferreira T, Miraglio B, Timonen S, Kettunen J, Pirinen M, Karjalainen J, Thorleifsson G, Hagg S, Hottenga JJ, Isaacs A, Ladenvall C, Beekman M, Esko T, Ried JS, Nelson CP, Willenborg C, Gustafsson S, Westra HJ, Blades M, de Craen AJ, de Geus EJ, Deelen J, Grallert H, Hamsten A, Havulinna AS, Hengstenberg C, Houwing-Duistermaat JJ, Hypponen E, Karssen LC, Lehtimaki T, Lyssenko V, Magnusson PK, Mihailov E, Muller-Nurasyid M, Mpindi JP, Pedersen NL, Penninx BW, Perola M, Pers TH, Peters A, Rung J, Smit JH, Steinthorsdottir V, Tobin MD, Tsernikova N, van Leeuwen EM, Viikari JS, Willems SM, Willemsen G, Schunkert H, Erdmann J, Samani NJ, Kaprio J, Lind L, Gieger C, Metspalu A, Slagboom PE, Groop L, van Duijn CM, Eriksson JG, Jula A, Salomaa V, Boomsma DI, Power C, Raitakari OT, Ingelsson E, Jarvelin MR, Thorsteinsdottir U, Franke L, Ikonen E, Kallioniemi O, Pietiainen V, Lindgren CM, Stefansson K, Palotie A, McCarthy MI, Morris AP, Prokopenko I, Ripatti S (2015). The impact of low-frequency and rare variants on lipid levels. Nat Genet.

[60] Kathiresan S, Willer CJ, Peloso GM, Demissie S, Musunuru K, Schadt EE, Kaplan L, Bennett D, Li Y, Tanaka T, Voight BF, Bonnycastle LL, Jackson AU, Crawford G, Surti A, Guiducci C, Burtt NP, Parish S, Clarke R, Zelenika D, Kubalanza KA, Morken MA, Scott LJ, Stringham HM, Galan P, Swift AJ, Kuusisto J, Bergman RN, Sundvall J, Laakso M, Ferrucci L, Scheet P, Sanna S, Uda M, Yang Q, Lunetta KL, Dupuis J, de Bakker PI, O’Donnell CJ, Chambers JC, Kooner JS, Hercberg S, Meneton P, Lakatta EG, Scuteri A, Schlessinger D, Tuomilehto J, Collins FS, Groop L, Altshuler D, Collins R, Lathrop GM, Melander O, Salomaa V, Peltonen L, Orho-Melander M, Ordovas JM, Boehnke M, Abecasis GR, Mohlke KL, Cupples LA (2009). Common variants at 30 loci contribute to polygenic dyslipidemia. Nat Genet.

[61] Willer CJ, Schmidt EM, Sengupta S, Peloso GM, Gustafsson S, Kanoni S, Ganna A, Chen J, Buchkovich ML, Mora S, Beckmann JS, Bragg-Gresham JL, Chang HY, Demirkan A, Den Hertog HM, Do R, Donnelly LA, Ehret GB, Esko T, Feitosa MF, Ferreira T, Fischer K, Fontanillas P, Fraser RM, Freitag DF, Gurdasani D, Heikkila K, Hypponen E, Isaacs A, Jackson AU, Johansson A, Johnson T, Kaakinen M, Kettunen J, Kleber ME, Li X, Luan J, Lyytikainen LP, Magnusson PKE, Mangino M, Mihailov E, Montasser ME, Muller-Nurasyid M, Nolte IM, O’Connell JR, Palmer CD, Perola M, Petersen AK, Sanna S, Saxena R, Service SK, Shah S, Shungin D, Sidore C, Song C, Strawbridge RJ, Surakka I, Tanaka T, Teslovich TM, Thorleifsson G, Van den Herik EG, Voight BF, Volcik KA, Waite LL, Wong A, Wu Y, Zhang W, Absher D, Asiki G, Barroso I, Been LF, Bolton JL, Bonnycastle LL, Brambilla P, Burnett MS, Cesana G, Dimitriou M, Doney ASF, Doring A, Elliott P, Epstein SE, Ingi Eyjolfsson G, Gigante B, Goodarzi MO, Grallert H, Gravito ML, Groves CJ, Hallmans G, Hartikainen AL, Hayward C, Hernandez D, Hicks AA, Holm H, Hung YJ, Illig T, Jones MR, Kaleebu P, Kastelein JJP, Khaw KT, Kim E, Klopp N, Komulainen P, Kumari M, Langenberg C, Lehtimaki T, Lin SY, Lindstrom J, Loos RJF, Mach F, McArdle WL, Meisinger C, Mitchell BD, Muller G, Nagaraja R, Narisu N, Nieminen TVM, Nsubuga RN, Olafsson I, Ong KK, Palotie A, Papamarkou T, Pomilla C, Pouta A, Rader DJ, Reilly MP, Ridker PM, Rivadeneira F, Rudan I, Ruokonen A, Samani N, Scharnagl H, Seeley J, Silander K, Stan?akova A, Stirrups K, Swift AJ, Tiret L, Uitterlinden AG, van Pelt LJ, Vedantam S, Wainwright N, Wijmenga C, Wild SH, Willemsen G, Wilsgaard T, Wilson JF, Young EH, Zhao JH, Adair LS, Arveiler D, Assimes TL, Bandinelli S, Bennett F, Bochud M, Boehm BO, Boomsma DI, Borecki IB, Bornstein SR, Bovet P, Burnier M, Campbell H, Chakravarti A, Chambers JC, Chen YI, Collins FS, Cooper RS, Danesh J, Dedoussis G, de Faire U, Feranil AB, Ferrieres J, Ferrucci L, Freimer NB, Gieger C, Groop LC, Gudnason V, Gyllensten U, Hamsten A, Harris TB, Hingorani A, Hirschhorn JN, Hofman A, Hovingh GK, Hsiung CA, Humphries SE, Hunt SC, Hveem K, Iribarren C, Jarvelin MR, Jula A, Kahonen M, Kaprio J, Kesaniemi A, Kivimaki M, Kooner JS, Koudstaal PJ, Krauss RM, Kuh D, Kuusisto J, Kyvik KO, Laakso M, Lakka TA, Lind L, Lindgren CM, Martin NG, Marz W, McCarthy MI, McKenzie CA, Meneton P, Metspalu A, Moilanen L, Morris AD, Munroe PB, Nj?lstad I, Pedersen NL, Power C. Discovery and refinement of loci associated with lipid levels. Nat Genet. 2013; 45(11):1274–83. 10.1038/ng.2797 PMC3838666 24097068

[62] Nagy R, Boutin TS, Marten J, Huffman JE, Kerr SM, Campbell A, Evenden L, Gibson J, Amador C, Howard DM, Navarro P, Morris A, Deary IJ, Hocking LJ, Padmanabhan S, Smith BH, Joshi P, Wilson JF, Hastie ND, Wright AF, McIntosh AM, Porteous DJ, Haley CS, Vitart V, Hayward C (2017). Exploration of haplotype research consortium imputation for genome-wide association studies in 20,032 Generation Scotland participants. Genome Med.

[63] Waterworth DM, Ricketts SL, Song K, Chen L, Zhao JH, Ripatti S, Aulchenko YS, Zhang W, Yuan X, Lim N, Luan J, Ashford S, Wheeler E, Young EH, Hadley D, Thompson JR, Braund PS, Johnson T, Struchalin M, Surakka I, Luben R, Khaw KT, Rodwell SA, Loos RJ, Boekholdt SM, Inouye M, Deloukas P, Elliott P, Schlessinger D, Sanna S, Scuteri A, Jackson A, Mohlke KL, Tuomilehto J, Roberts R, Stewart A, Kesaniemi YA, Mahley RW, Grundy SM, McArdle W, Cardon L, Waeber G, Vollenweider P, Chambers JC, Boehnke M, Abecasis GR, Salomaa V, Jarvelin MR, Ruokonen A, Barroso I, Epstein SE, Hakonarson HH, Rader DJ, Reilly MP, Witteman JC, Hall AS, Samani NJ, Strachan DP, Barter P, van Duijn CM, Kooner JS, Peltonen L, Wareham NJ, McPherson R, Mooser V, Sandhu MS (2010). Genetic variants influencing circulating lipid levels and risk of coronary artery disease. Arterioscler Thromb Vasc Biol.

[64] Spracklen CN, Chen P, Kim YJ, Wang X, Cai H, Li S, Long J, Wu Y, Wang YX, Takeuchi F, Wu JY, Jung KJ, Hu C, Akiyama K, Zhang Y, Moon S, Johnson TA, Li H, Dorajoo R, He M, Cannon ME, Roman TS, Salfati E, Lin KH, Guo X, Sheu WHH, Absher D, Adair LS, Assimes TL, Aung T, Cai Q, Chang LC, Chen CH, Chien LH, Chuang LM, Chuang SC, Du S, Fan Q, Fann CSJ, Feranil AB, Friedlander Y, Gordon-Larsen P, Gu D, Gui L, Guo Z, Heng CK, Hixson J, Hou X, Hsiung CA, Hu Y, Hwang MY, Hwu CM, Isono M, Juang JJ, Khor CC, Kim YK, Koh WP, Kubo M, Lee IT, Lee SJ, Lee WJ, Liang KW, Lim B, Lim SH, Liu J, Nabika T, Pan WH, Peng H, Quertermous T, Sabanayagam C, Sandow K, Shi J, Sun L, Tan PC, Tan SP, Taylor KD, Teo YY, Toh SA, Tsunoda T, van Dam RM, Wang A, Wang F, Wang J, Wei WB, Xiang YB, Yao J, Yuan JM, Zhang R, Zhao W, Chen YI, Rich SS, Rotter JI, Wang TD, Wu T, Lin X, Han BG, Tanaka T, Cho YS, Katsuya T, Jia W, Jee SH, Chen YT, Kato N, Jonas JB, Cheng CY, Shu XO, He J, Zheng W, Wong TY, Huang W, Kim BJ, Tai ES, Mohlke KL, Sim X. Association analyses of East Asian individuals and trans-ancestry analyses with European individuals reveal new loci associated with cholesterol and triglyceride levels. Hum Mol Genet. 2017; 26(9):1770–84. 10.1093/hmg/ddx062 PMC6075203 28334899

[65] Teslovich TM, Musunuru K, Smith AV, Edmondson AC, Stylianou IM, Koseki M, Pirruccello JP, Ripatti S, Chasman DI, Willer CJ, Johansen CT, Fouchier SW, Isaacs A, Peloso GM, Barbalic M, Ricketts SL, Bis JC, Aulchenko YS, Thorleifsson G, Feitosa MF, Chambers J, Orho-Melander M, Melander O, Johnson T, Li X, Guo X, Li M, Shin Cho Y, Jin Go M, Jin Kim Y, Lee JY, Park T, Kim K, Sim X, Twee-Hee Ong R, Croteau-Chonka DC, Lange LA, Smith JD, Song K, Hua Zhao J, Yuan X, Luan J, Lamina C, Ziegler A, Zhang W, Zee RY, Wright AF, Witteman JC, Wilson JF, Willemsen G, Wichmann HE, Whitfield JB, Waterworth DM, Wareham NJ, Waeber G, Vollenweider P, Voight BF, Vitart V, Uitterlinden AG, Uda M, Tuomilehto J, Thompson JR, Tanaka T, Surakka I, Stringham HM, Spector TD, Soranzo N, Smit JH, Sinisalo J, Silander K, Sijbrands EJ, Scuteri A, Scott J, Schlessinger D, Sanna S, Salomaa V, Saharinen J, Sabatti C, Ruokonen A, Rudan I, Rose LM, Roberts R, Rieder M, Psaty BM, Pramstaller PP, Pichler I, Perola M, Penninx BW, Pedersen NL, Pattaro C, Parker AN, Pare G, Oostra BA, O’Donnell CJ, Nieminen MS, Nickerson DA, Montgomery GW, Meitinger T, McPherson R, McCarthy MI, McArdle W, Masson D, Martin NG, Marroni F, Mangino M, Magnusson PK, Lucas G, Luben R, Loos RJ, Lokki ML, Lettre G, Langenberg C, Launer LJ, Lakatta EG, Laaksonen R, Kyvik KO, Kronenberg F, Konig IR, Khaw KT, Kaprio J, Kaplan LM, Johansson A, Jarvelin MR, Janssens AC, Ingelsson E, Igl W, Kees Hovingh G, Hottenga JJ, Hofman A, Hicks AA, Hengstenberg C, Heid IM, Hayward C, Havulinna AS, Hastie ND, Harris TB, Haritunians T, Hall AS, Gyllensten U, Guiducci C, Groop LC, Gonzalez E, Gieger C, Freimer NB, Ferrucci L, Erdmann J, Elliott P, Ejebe KG, Doring A, Dominiczak AF, Demissie S, Deloukas P, de Geus EJ, de Faire U, Crawford G, Collins FS, Chen YD, Caulfield MJ, Campbell H, Burtt NP, Bonnycastle LL, Boomsma DI, Boekholdt SM, Bergman RN, Barroso I, Bandinelli S, Ballantyne CM, Assimes TL, Quertermous T, Altshuler D, Seielstad M, Wong TY, Tai ES, Feranil AB, Kuzawa CW, Adair LS, Taylor HA, Borecki IB, Gabriel SB, Wilson JG, Holm H, Thorsteinsdottir U, Gudnason V, Krauss RM, Mohlke KL, Ordovas JM, Munroe PB, Kooner JS, Tall AR, Hegele RA, Kastelein JJ, Schadt EE, Rotter JI, Boerwinkle E, Strachan DP, Mooser V, Stefansson K, Reilly MP, Samani NJ, Schunkert H, Cupples LA, Sandhu MS, Ridker PM, Rader DJ, van Duijn CM, Peltonen L, Abecasis GR, Boehnke M, Kathiresan S (2010). Biological, clinical and population relevance of 95 loci for blood lipids. Nature.

[66] Ligthart S, Vaez A, Hsu YH, Stolk R, Uitterlinden AG, Hofman A, Alizadeh BZ, Franco OH, Dehghan A (2016). Bivariate genome-wide association study identifies novel pleiotropic loci for lipids and inflammation. BMC Genomics.

[67] Lango Allen H, Estrada K, Lettre G, Berndt SI, Weedon MN, Rivadeneira F, Willer CJ, Jackson AU, Vedantam S, Raychaudhuri S, Ferreira T, Wood AR, Weyant RJ, Segr? AV, Speliotes EK, Wheeler E, Soranzo N, Park JH, Yang J, Gudbjartsson D, Heard-Costa NL, Randall JC, Qi L, Vernon Smith A, Mogi R, Pastinen T, Liang L, Heid IM, Luan J, Thorleifsson G, Winkler TW, Goddard ME, Sin Lo K, Palmer C, Workalemahu T, Aulchenko YS, Johansson A, Zillikens MC, Feitosa MF, Esko T, Johnson T, Ketkar S, Kraft P, Mangino M, Prokopenko I, Absher D, Albrecht E, Ernst F, Glazer NL, Hayward C, Hottenga JJ, Jacobs KB, Knowles JW, Kutalik Z, Monda KL, Polasek O, Preuss M, Rayner NW, Robertson NR, Steinthorsdottir V, Tyrer JP, Voight BF, Wiklund F, Xu J, Zhao JH, Nyholt DR, Pellikka N, Perola M, Perry JR, Surakka I, Tammesoo ML, Altmaier EL, Amin N, Aspelund T, Bhangale T, Boucher G, Chasman DI, Chen C, Coin L, Cooper MN, Dixon AL, Gibson Q, Grundberg E, Hao K, Juhani Junttila M, Kaplan LM, Kettunen J, K?nig IR, Kwan T, Lawrence RW, Levinson DF, Lorentzon M, McKnight B, Morris AP, Mller M, Suh Ngwa J, Purcell S, Rafelt S, Salem RM, Salvi E, Sanna S, Shi J, Sovio U, Thompson JR, Turchin MC, Vandenput L, Verlaan DJ, Vitart V, White CC, Ziegler A, Almgren P, Balmforth AJ, Campbell H, Citterio L, De Grandi A, Dominiczak A, Duan J, Elliott P, Elosua R, Eriksson JG, Freimer NB, Geus EJ, Glorioso N, Haiqing S, Hartikainen AL, Havulinna AS, Hicks AA, Hui J, Igl W, Illig T, Jula A, Kajantie E, Kilpel?inen TO, Koiranen M, Kolcic I, Koskinen S, Kovacs P, Laitinen J, Liu J, Lokki ML, Marusic A, Maschio A, Meitinger T, Mulas A, Par G, Parker AN, Peden JF, Petersmann A, Pichler I, Pietilinen KH, Pouta A, Ridderstrle M, Rotter JI, Sambrook JG, Sanders AR, Schmidt CO, Sinisalo J, Smit JH, Stringham HM, Bragi Walters G, Widen E, Wild SH, Willemsen G, Zagato L, Zgaga L, Zitting P, Alavere H, Farrall M, McArdle WL, Nelis M, Peters MJ, Ripatti S, van Meurs JB, Aben KK, Ardlie KG, Beckmann JS, Beilby JP, Bergman RN, Bergmann S, Collins FS, Cusi D, den Heijer M, Eiriksdottir G, Gejman PV, Hall AS, Hamsten A, Huikuri HV, Iribarren C, K?h?nen M, Kaprio J, Kathiresan S, Kiemeney L, Kocher T, Launer LJ, Lehtim?ki T, Melander O, Mosley TH, Musk AW, Nieminen MS, O’Donnell CJ, Ohlsson C, Oostra B, Palmer LJ, Raitakari O, Ridker PM, Rioux JD, Rissanen A, Rivolta C, Schunkert H, Shuldiner AR, Siscovick DS, Stumvoll M, T?njes A, Tuomilehto J, van Ommen GJ, Viikari J, Heath AC, Martin NG, Montgomery GW, Province MA, Kayser M, Arnold AM, Atwood LD. Hundreds of variants clustered in genomic loci and biological pathways affect human height. Nature. 2010; 467(7317):832–8. 10.1038/nature09410 PMC2955183 20881960

[68] Botstein D, Risch N (2003). Discovering genotypes underlying human phenotypes: past successes for mendelian disease, future approaches for complex disease. Nat Genet.

[69] Kann MG (2010). Advances in translational bioinformatics: computational approaches for the hunting of disease genes. Brief Bioinform.

[70] Liu JZ, McRae AF, Nyholt DR, Medland SE, Wray NR, Brown KM, Hayward NK, Montgomery GW, Visscher PM, Martin NG, Macgregor S, Mann GJ, Kefford RF, Hopper JL, Aitken JF, Giles GG, Armstrong BK (2010). A versatile gene-based test for genome-wide association studies. Am J Hum Genet.

[71] Li MX, Kwan JS, Sham PC (2012). HYST: a hybrid set-based test for genome-wide association studies, with application to protein-protein interaction-based association analysis. Am J Hum Genet.

[72] Hormozdiari F, van de Bunt M, Segre AV, Li X, Joo JWJ, Bilow M, Sul JH, Sankararaman S, Pasaniuc B, Eskin E (2016). Colocalization of GWAS and eQTL signals detects target genes. Am J Hum Genet.

[73] Consortium GP (2015). A global reference for human genetic variation. Nature.

[74] Bauer DC, Zadoorian A, Wilson LO, Alliance MGH, Thorne NP (2018). Evaluation of computational programs to predict HLA genotypes from genomic sequencing data. Brief Bioinform.

[75] Eskin E (2008). Increasing power in association studies by using linkage disequilibrium structure and molecular function as prior information. Genome Res.

[76] Wen X (2016). Molecular QTL discovery incorporating genomic annotations using Bayesian false discovery rate control. Ann Appl Stat.

[77] Duong D, Zou J, Hormozdiari F, Sul JH, Ernst J, Han B, Eskin E (2016). Using genomic annotations increases statistical power to detect eGenes. Bioinformatics.

[78] Hormozdiari F, Van De Bunt M, Segre AV, Li X, Joo JWJ, Bilow M, Sul JH, Sankararaman S, Pasaniuc B, Eskin E (2016). Colocalization of GWAS and eQTL signals detects target genes. Am J Hum Genet.

[79] Han B, Kang HM, Eskin E (2009). Rapid and accurate multiple testing correction and power estimation for millions of correlated markers. PLoS Genet.

[80] Hormozdiari F, Kichaev G, Yang W-Y, Pasaniuc B, Eskin E (2015). Identification of causal genes for complex traits. Bioinformatics.

[81] Eskin E (2008). Increasing power in association studies by using linkage disequilibrium structure and molecular function as prior information. Genome Res.

[82] Darnell G, Duong D, Han B, Eskin E (2012). Incorporating prior information into association studies. Bioinformatics.

[83] Sul JH, Han B, He D, Eskin E (2011). An optimal weighted aggregated association test for identification of rare variants involved in common diseases. Genetics.

[84] Hajivassiliou V, McFadden D, Ruud P (1996). Simulation of multivariate normal rectangle probabilities and their derivatives theoretical and computational results. J Econ.

[85] Yang J, Lee SH, Goddard ME, Visscher PM (2011). GCTA: a tool for genome-wide complex trait analysis. Am J Hum Genet.

[86] Hormozdiari F, Jung J, Eskin E, Joo JWJ. MARS: Leveraging allelic heterogeneity to increase power of association testing. GitHub. 2021. https://github.com/junghyunJJ/marsR. 10.1186/s13059-021-02353-8 PMC8086090 33931127

[87] Hormozdiari F, Jung J, Eskin E, Joo JWJ. MARS: Leveraging allelic heterogeneity to increase power of association testing. 2021. 10.5281/zenodo.4679205. 10.1186/s13059-021-02353-8 PMC8086090 33931127

