# Supplementary material for: MARS: leveraging allelic heterogeneity to increase power of association testing
Source: Genome Biol. 2021 Apr 30;22:128. doi: 10.1186/s13059-021-02353-8 (PMC8086090; doi:10.1186/s13059-021-02353-8)
Supplement: Supplementary file 1 — Additional file 1 Fig. S2— MARS detects more eGenes in GTEx Whole Blood data. Fig. S3— Venn diagrams comparing eGenes identified by MARS using GTEx v6, eGenes reported by GTEx v6, and eGenes reported by GTEx v7 for all the tissues. Fig. S4— Compare p-values estimated from the standard sampling and those estimated from the importance sampling. Fig. S5— Venn diagrams comparing set-based association identified by MARS and the univariate test for traits in NFBC data. Fig. S6— Number of loci found by previous studies among the loci identified by MARS but not by the univariate test. Fig. S7—Power of MARS on the choice of priors on gamma. Fig. S8— Using only top 50 SNPs with bigger summary statistics well approximates the result using all the SNPs in MARS. [file 13059_2021_2353_MOESM1_ESM.pdf]

MARS: Leveraging allelic heterogeneity to increase power of association testing

*Farhad Hormozdiari†, Junghyun Jung†, Eleazar Eskin, Jong Wha J. Joo**

**Corresponding author: jwjjoo@dongguk.edu*
†They contributed equally to this work.

Additional Files

Fig. S1. Power of MARS compared to SKAT, SKAT-O, and Meta-SKAT in simulated studies.

Fig. S2. MARS detects more eGenes in GTEx Whole Blood data.

Fig. S3. Venn diagrams comparing eGenes identified by MARS using GTEx v6, eGenes reported by GTEx v6, and eGenes reported by GTEx v7 for all the tissues.

Fig. S4. Compare *p*-values estimated from the standard sampling and those estimated from the importance sampling.

Fig. S5. Venn diagrams comparing set-based association identified by MARS and the univariate test for traits in NFBC data.

Fig. S6. Number of loci found by previous studies among the loci identified by MARS but not by the univariate test.

Fig. S7. Power of MARS on the choice of priors on gamma.

Fig. S8. Using only top 50 SNPs with bigger summary statistics well approximates the result using all the SNPs in MARS.

Fig. S1. Power of MARS compared to SKAT, SKAT-O, and Meta-SKAT in simulated studies

As SKAT does not allow summary statistics as their input, to compare powers of MARS with those to SKAT, SKAT-O, and Meta-SKAT [1], we simulated phenotype values with a range of effect sizes. We examined the power for cases with 2 causal SNPs implanted in the simulated data, where the 2 causal SNPs are randomly selected for each simulation. We ordered the SNPs by value of its summary statistics and used only top 50 SNPs for the analysis to reduce running time and space. For each simulation study, we generated 10^3 simulations and a threshold of 10^{-3} has been applied.

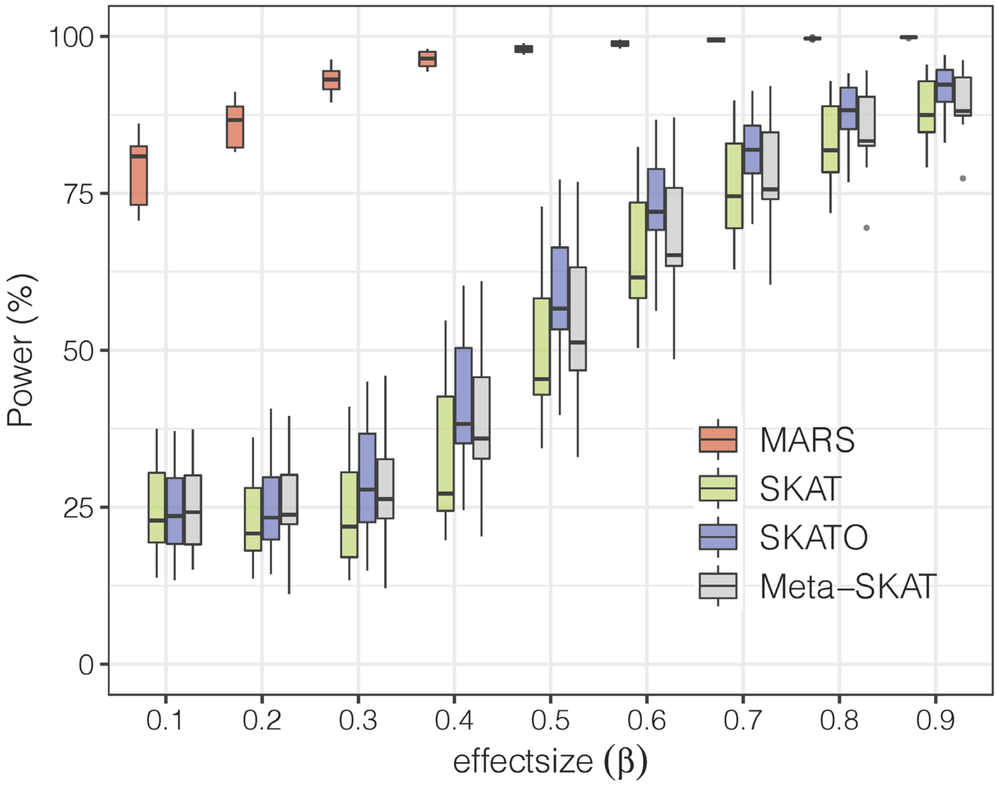

Fig. S1. Box plot showing the power of MARS, to SKAT, SKAT-O, and Meta-SKAT. The X-axis shows effect sizes of **β** in the simulated data set and the y-axis shows the power in percentage. The red bars, green bars, blue bars, and grey bars show power of MARS, SKAT, SKAT-O, and Meta-SKAT, respectively.

Fig. S2. MARS detects more eGenes in GTEx Whole Blood data

We applied MARS to genes from the Whole Blood data obtained from the GTEx consortium to show that MARS can detect more eGenes than the ones reported by the GTEx consortium. The Whole Blood data is used for the evaluation as this tissue contains the largest number of samples among the tissues provided by the GTEx consortium. To compare our results with GTEx's results, we use 10000 number of simulations because it is the number of simulations used by GTEx consortium to compute the empirical *p*-value to identify eGenes. For detecting eGenes, the threshold is defined as the border of empirical *p*-value between eGenes and genes other than those eGenes, non-eGenes, reported by the GTEx consortium.

We randomly selected 100 genes, where the half of the genes are eGenes and the rest half of the genes are non-eGenes according to GTEx consortium. We call these genes as eGenes_{gtex} and non-eGenes_{gtex}, respectively. We applied MARS and the univariate test to the genes and compared their *p*-values with those reported by the GTEx consortium. See the Materials and Methods for the detail. Fig. S2 (a) shows that for most of the non-eGenes_{gtex}, *p*-values of MARS are more significant than those of the univariate test as well as those reported by the GTEx consortium. Especially, MARS identifies six extra eGenes out of 50 non-eGenes_{gtex} that are missing from the GTEx consortium. The univariate test and GTEx show similar *p*-values in most of the genes as expected. Fig. S2 (b) shows that not only MARS identifies all the eGenes_{gtex} but also *p*-values of MARS for most of those genes are more significant than those of the univariate test as well as those reported by the GTEx consortium.

(a)

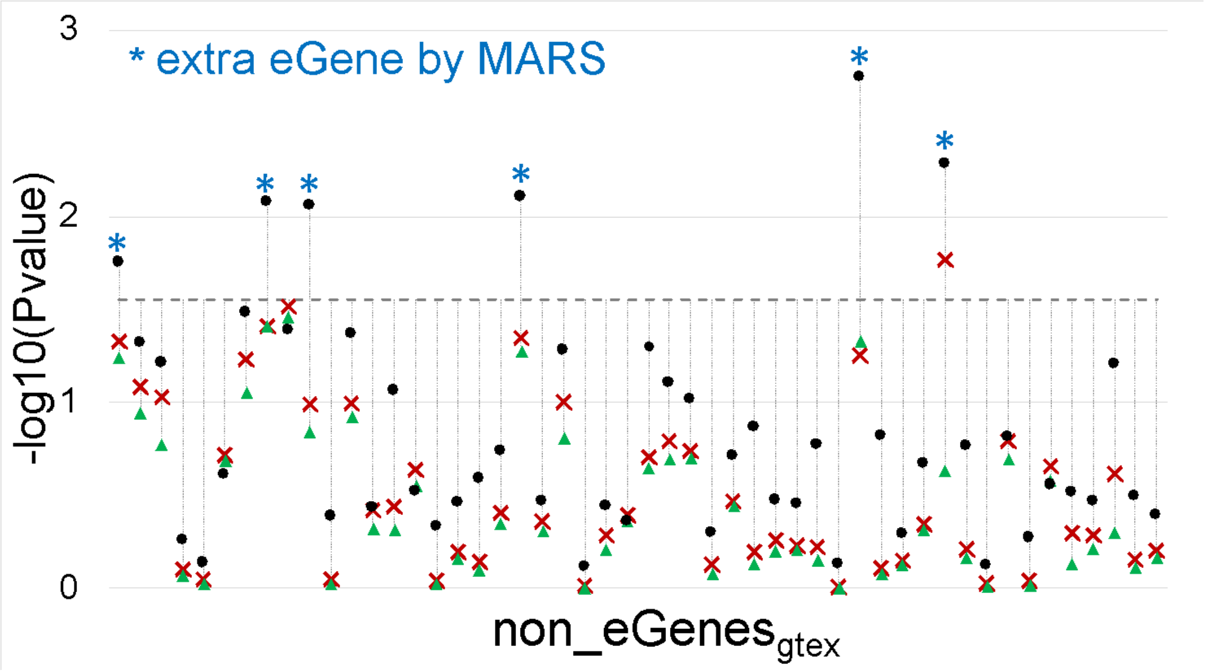

(b)

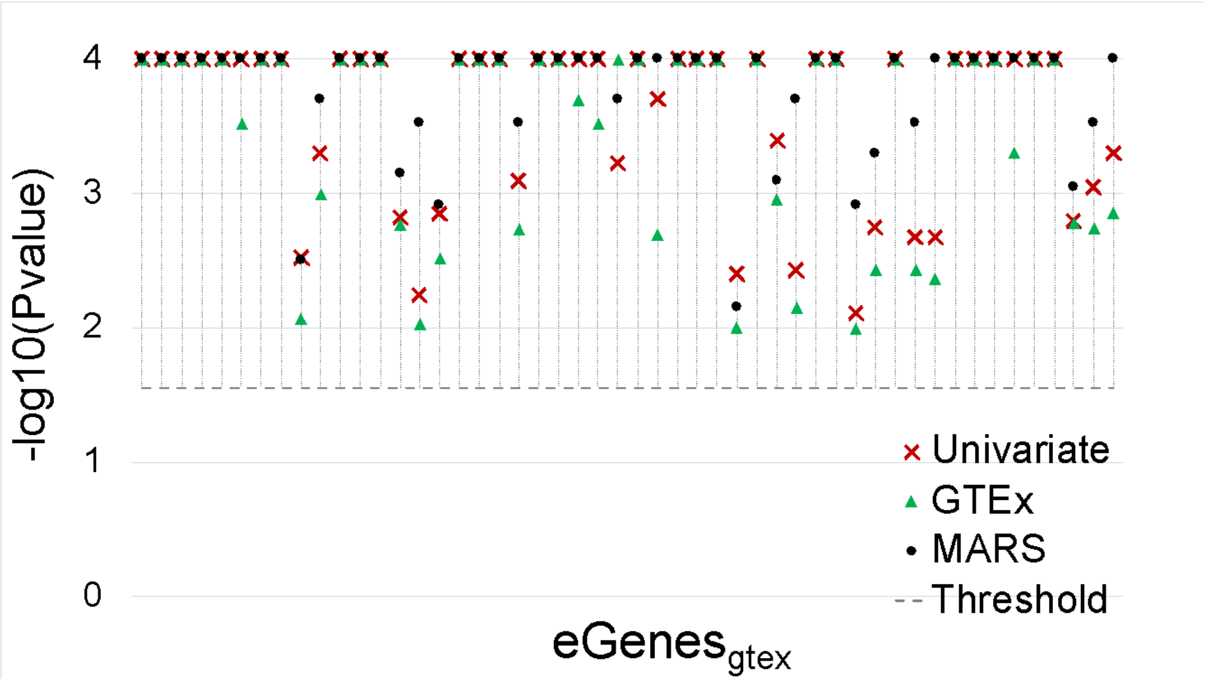

Fig. S2. MARS detects more eGenes in GTEx. The X-axis shows 50 non-eGenes$_{gtex}$ (a) and 50 eGenes$_{gtex}$ (b). The Y-axis shows the -log$_{10}$*p*-values of the genes. The black circle, red cross, and orange square shows -log$_{10}$*p*-values of MARS, the univariate test, and GTEx consortium, respectively. The grey dotted line shows the threshold of -log$_{10}$*p*-values estimated from empirical *p*-value reported by GTEx consortium.

Fig. S3. Venn diagrams comparing eGenes identified by MARS using GTEx v6, eGenes reported by GTEx v6, and eGenes reported by GTEx v7 for all the tissues.

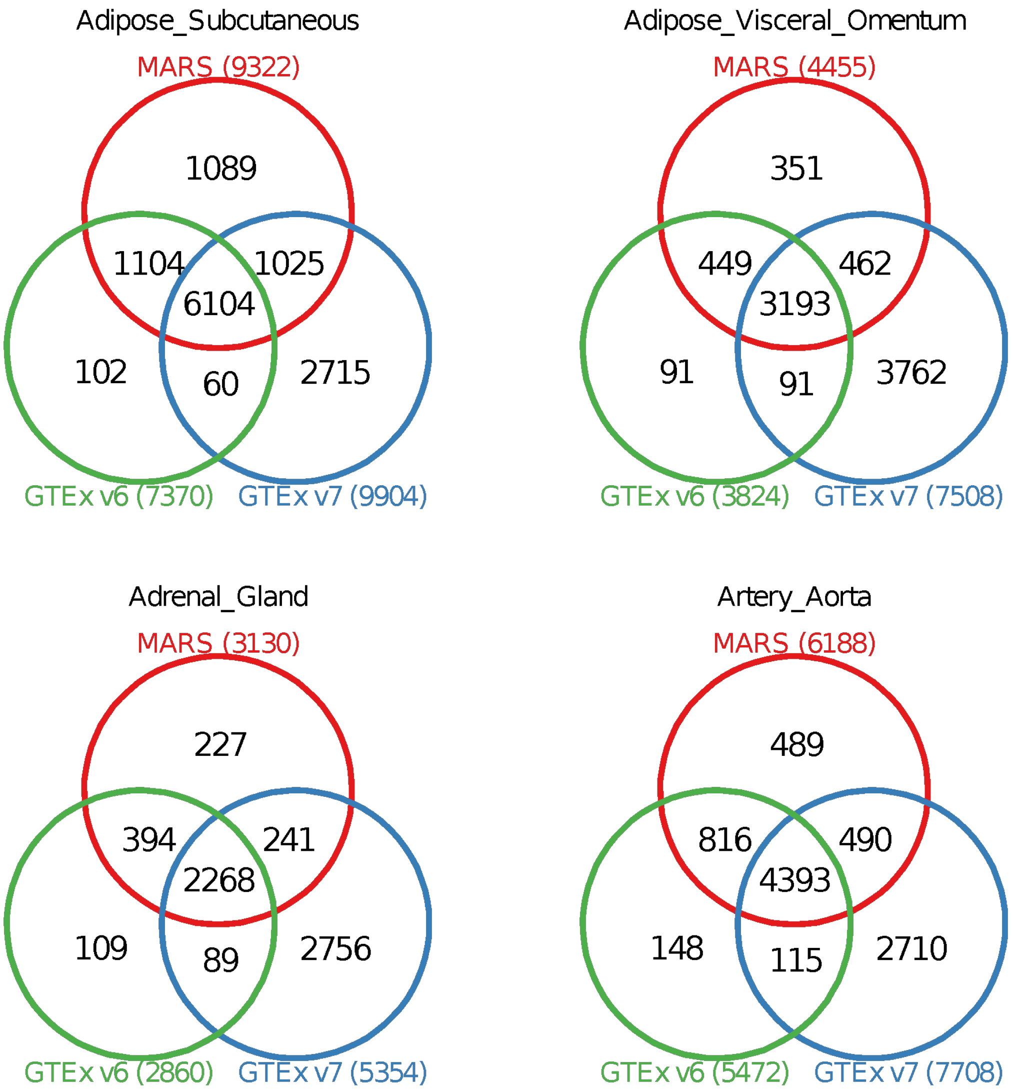

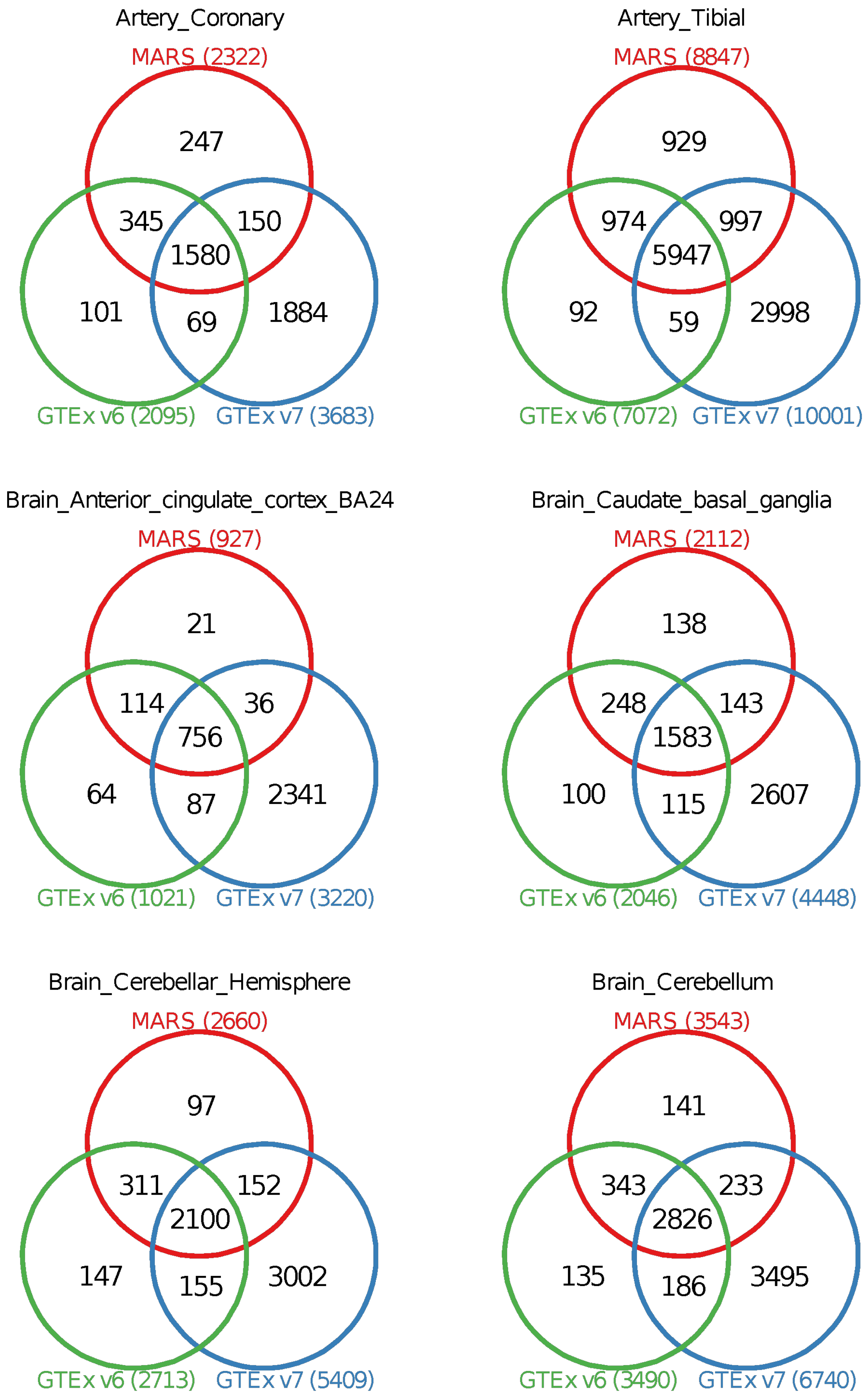

Artery_Coronary
MARS (2322)
247
345
150
1580
101
69
1884
GTEx v6 (2095)
GTEx v7 (3683)
Artery_Tibial
MARS (8847)
929
974
997
5947
92
59
2998
GTEx v6 (7072)
GTEx v7 (10001)
Brain_Anterior_cingulate_cortex_BA24
MARS (927)
21
114
36
756
64
87
2341
GTEx v6 (1021)
GTEx v7 (3220)
Brain_Caudate_basal_ganglia
MARS (2112)
138
248
143
1583
100
115
2607
GTEx v6 (2046)
GTEx v7 (4448)
Brain_Cerebellar_Hemisphere
MARS (2660)
97
311
152
2100
147
155
3002
GTEx v6 (2713)
GTEx v7 (5409)
Brain_Cerebellum
MARS (3543)
141
343
233
2826
135
186
3495
GTEx v6 (3490)
GTEx v7 (6740)

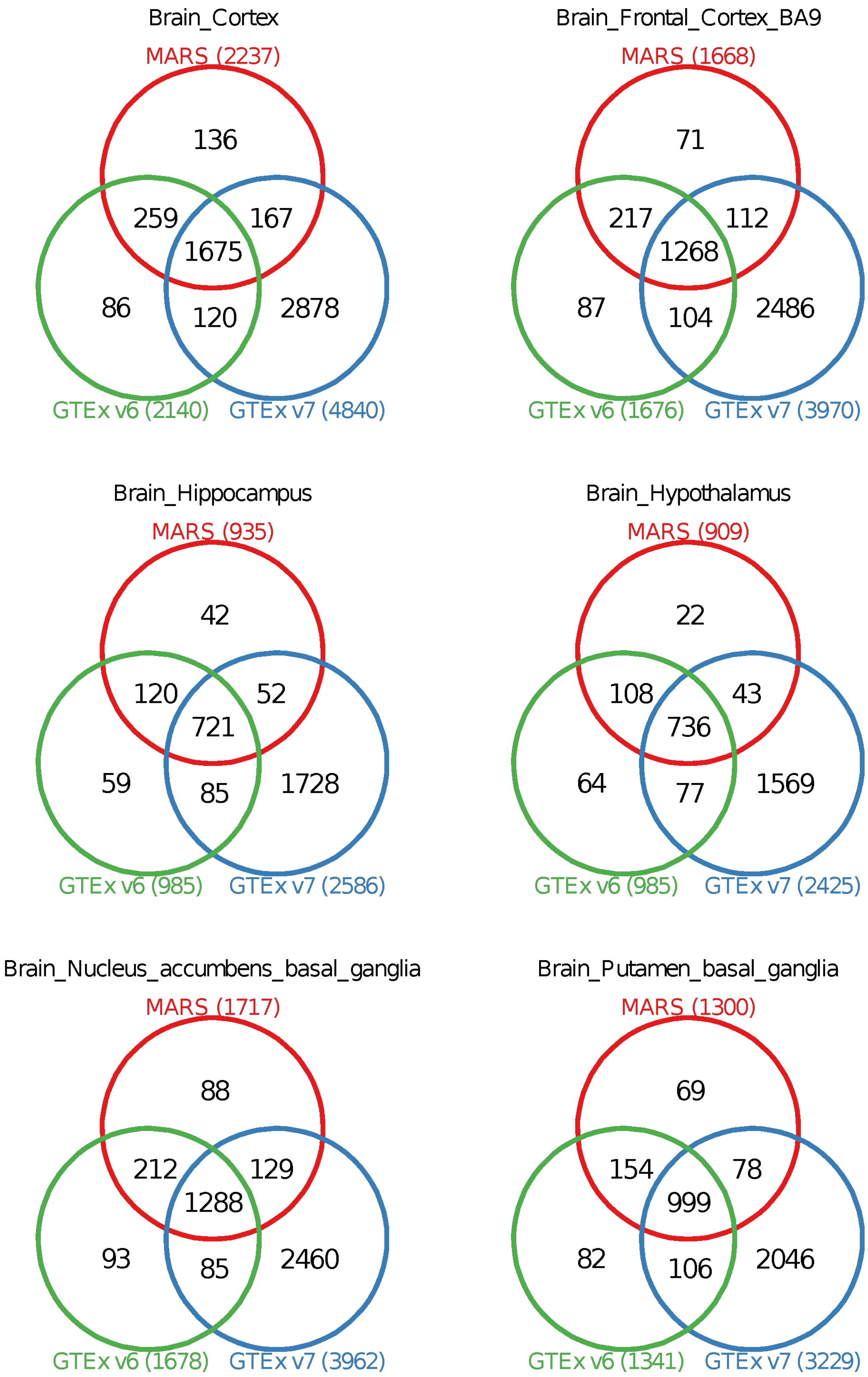
Brain_Cortex
MARS (2237)
136
259
167
1675
86
120
2878
GTEx v6 (2140)
GTEx v7 (4840)
Brain_Frontal_Cortex_BA9
MARS (1668)
71
217
112
1268
87
104
2486
GTEx v6 (1676)
GTEx v7 (3970)
Brain_Hippocampus
MARS (935)
42
120
52
721
59
85
1728
GTEx v6 (985)
GTEx v7 (2586)
Brain_Hypothalamus
MARS (909)
22
108
43
736
64
77
1569
GTEx v6 (985)
GTEx v7 (2425)
Brain_Nucleus_accumbens_basal_ganglia
MARS (1717)
88
212
129
1288
93
85
2460
GTEx v6 (1678)
GTEx v7 (3962)
Brain_Putamen_basal_ganglia
MARS (1300)
69
154
78
999
82
106
2046
GTEx v6 (1341)
GTEx v7 (3229)

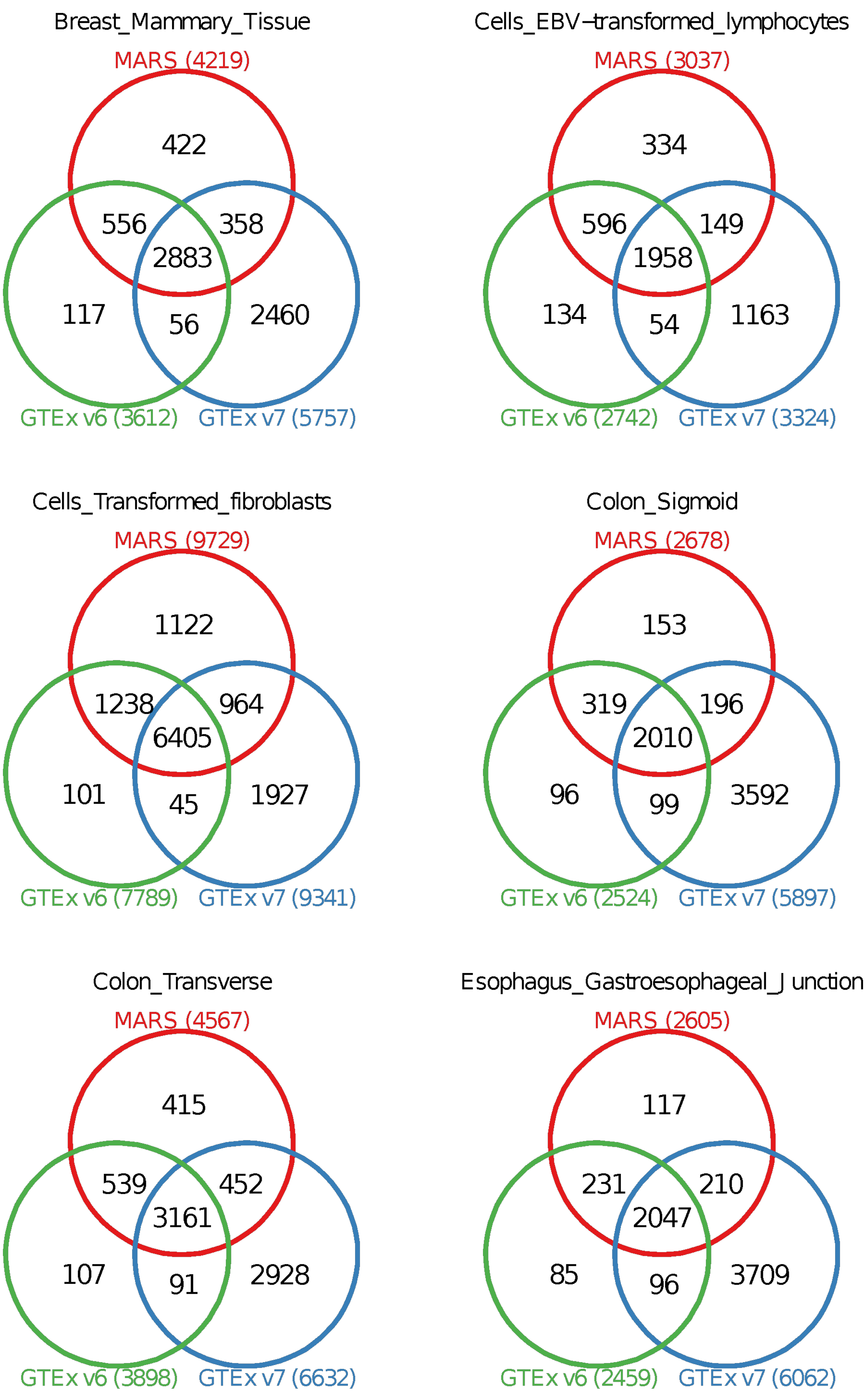

Breast_Mammary_Tissue
MARS (4219)
422
556
358
2883
117
56
2460
GTEx v6 (3612)
GTEx v7 (5757)
Cells_EBV−transformed_lymphocytes
MARS (3037)
334
596
149
1958
134
54
1163
GTEx v6 (2742)
GTEx v7 (3324)
Cells_Transformed_fibroblasts
MARS (9729)
1122
1238
964
6405
101
45
1927
GTEx v6 (7789)
GTEx v7 (9341)
Colon_Sigmoid
MARS (2678)
153
319
196
2010
96
99
3592
GTEx v6 (2524)
GTEx v7 (5897)
Colon_Transverse
MARS (4567)
415
539
452
3161
107
91
2928
GTEx v6 (3898)
GTEx v7 (6632)
Esophagus_Gastroesophageal_Junction
MARS (2605)
117
231
210
2047
85
96
3709
GTEx v6 (2459)
GTEx v7 (6062)

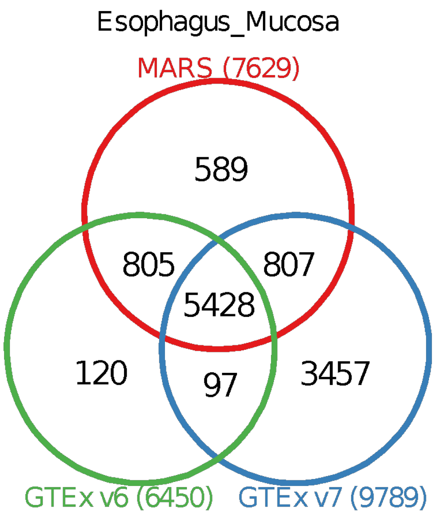
Esophagus_Mucosa
MARS (7629)
589
805
807
5428
120
97
3457
GTEx v6 (6450)
GTEx v7 (9789)

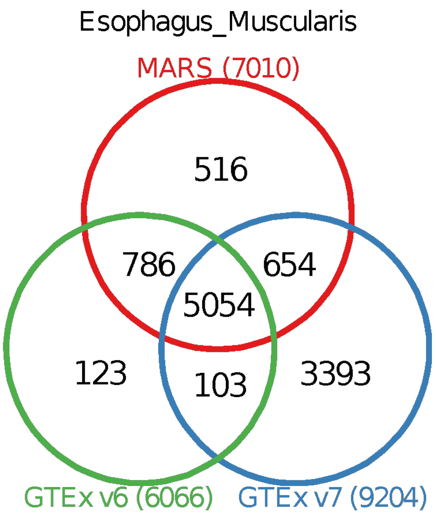
Esophagus_Muscularis
MARS (7010)
516
786
654
5054
123
103
3393
GTEx v6 (6066)
GTEx v7 (9204)

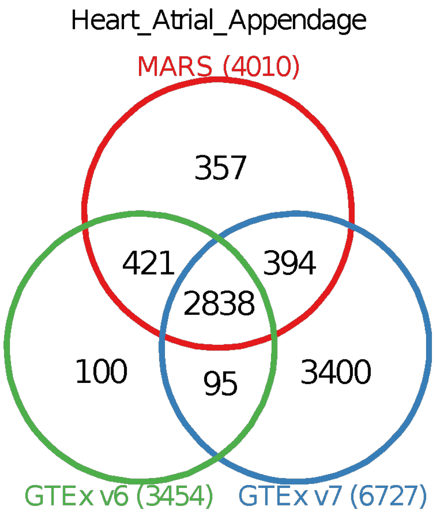
Heart_Atrial_Appendage
MARS (4010)
357
421
394
2838
100
95
3400
GTEx v6 (3454)
GTEx v7 (6727)

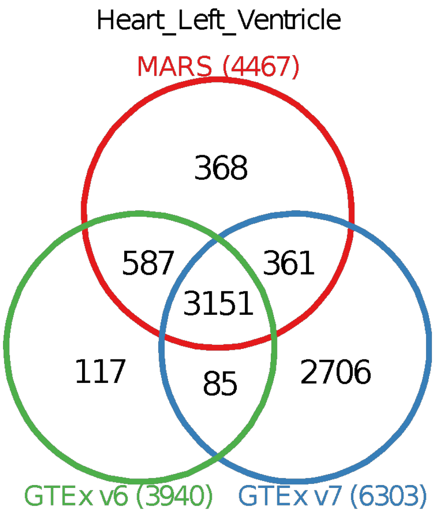
Heart_Left_Ventricle
MARS (4467)
368
587
361
3151
117
85
2706
GTEx v6 (3940)
GTEx v7 (6303)

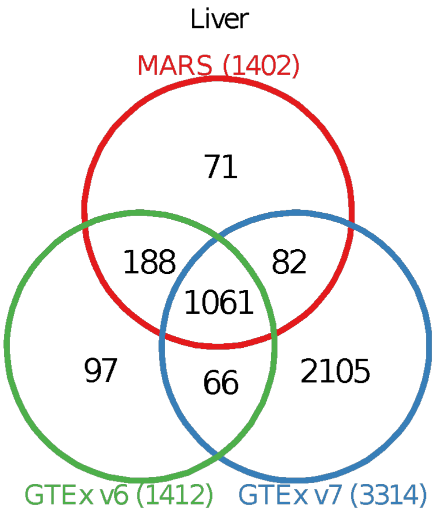
Liver
MARS (1402)
71
188
82
1061
97
66
2105
GTEx v6 (1412)
GTEx v7 (3314)

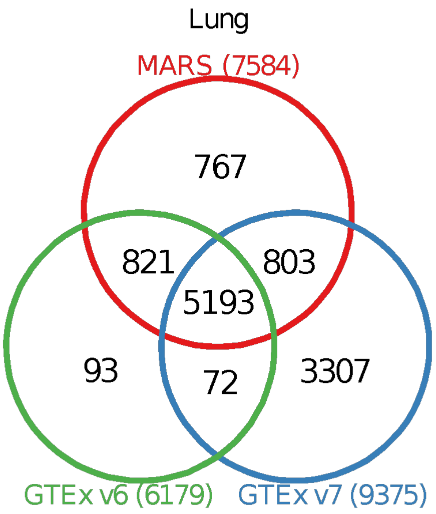
Lung
MARS (7584)
767
821
803
5193
93
72
3307
GTEx v6 (6179)
GTEx v7 (9375)

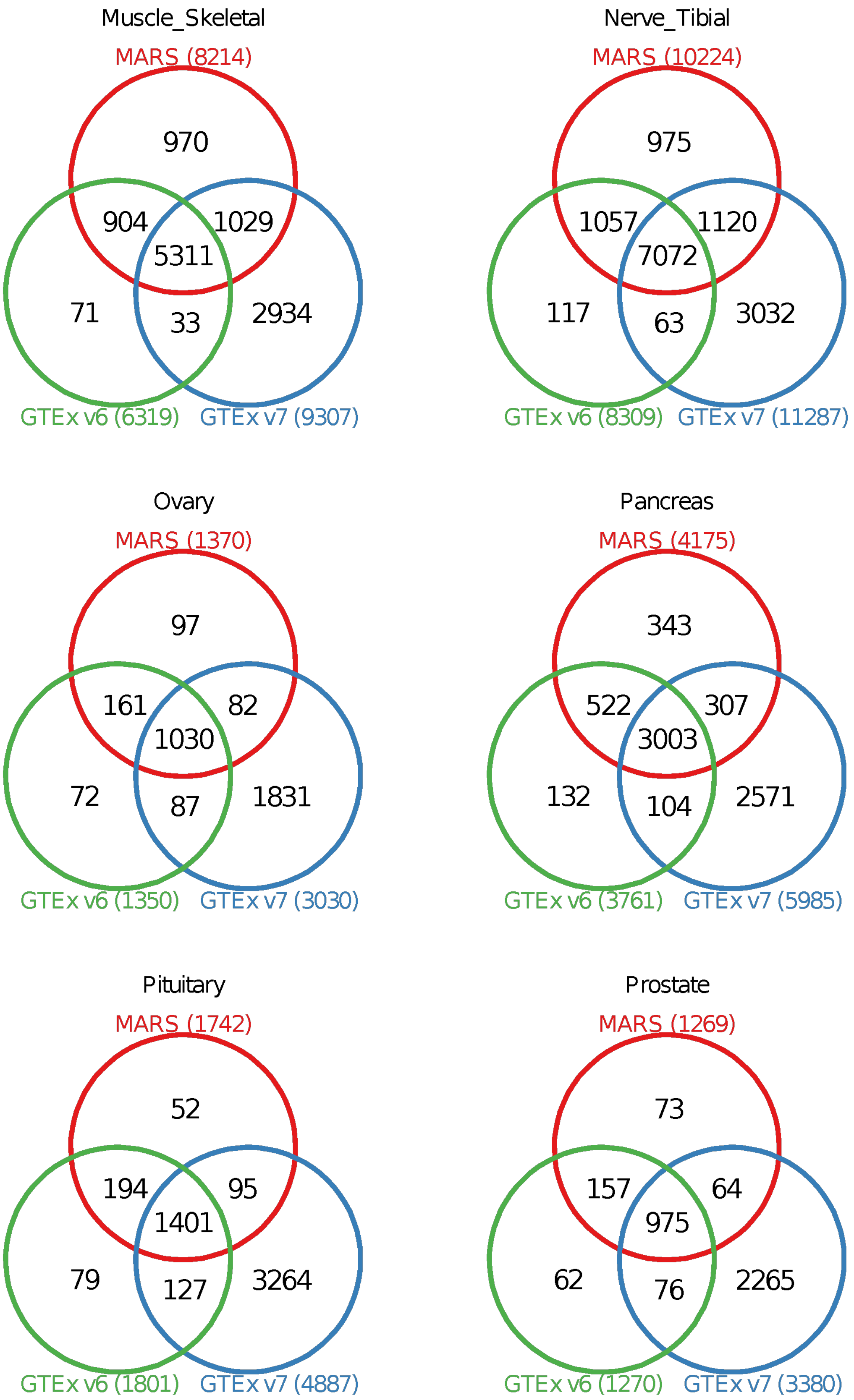
Muscle_Skeletal
MARS (8214)
970
904
1029
5311
71
33
2934
GTEx v6 (6319)
GTEx v7 (9307)
Nerve_Tibial
MARS (10224)
975
1057
1120
7072
117
63
3032
GTEx v6 (8309)
GTEx v7 (11287)
Ovary
MARS (1370)
97
161
82
1030
72
87
1831
GTEx v6 (1350)
GTEx v7 (3030)
Pancreas
MARS (4175)
343
522
307
3003
132
104
2571
GTEx v6 (3761)
GTEx v7 (5985)
Pituitary
MARS (1742)
52
194
95
1401
79
127
3264
GTEx v6 (1801)
GTEx v7 (4887)
Prostate
MARS (1269)
73
157
64
975
62
76
2265
GTEx v6 (1270)
GTEx v7 (3380)

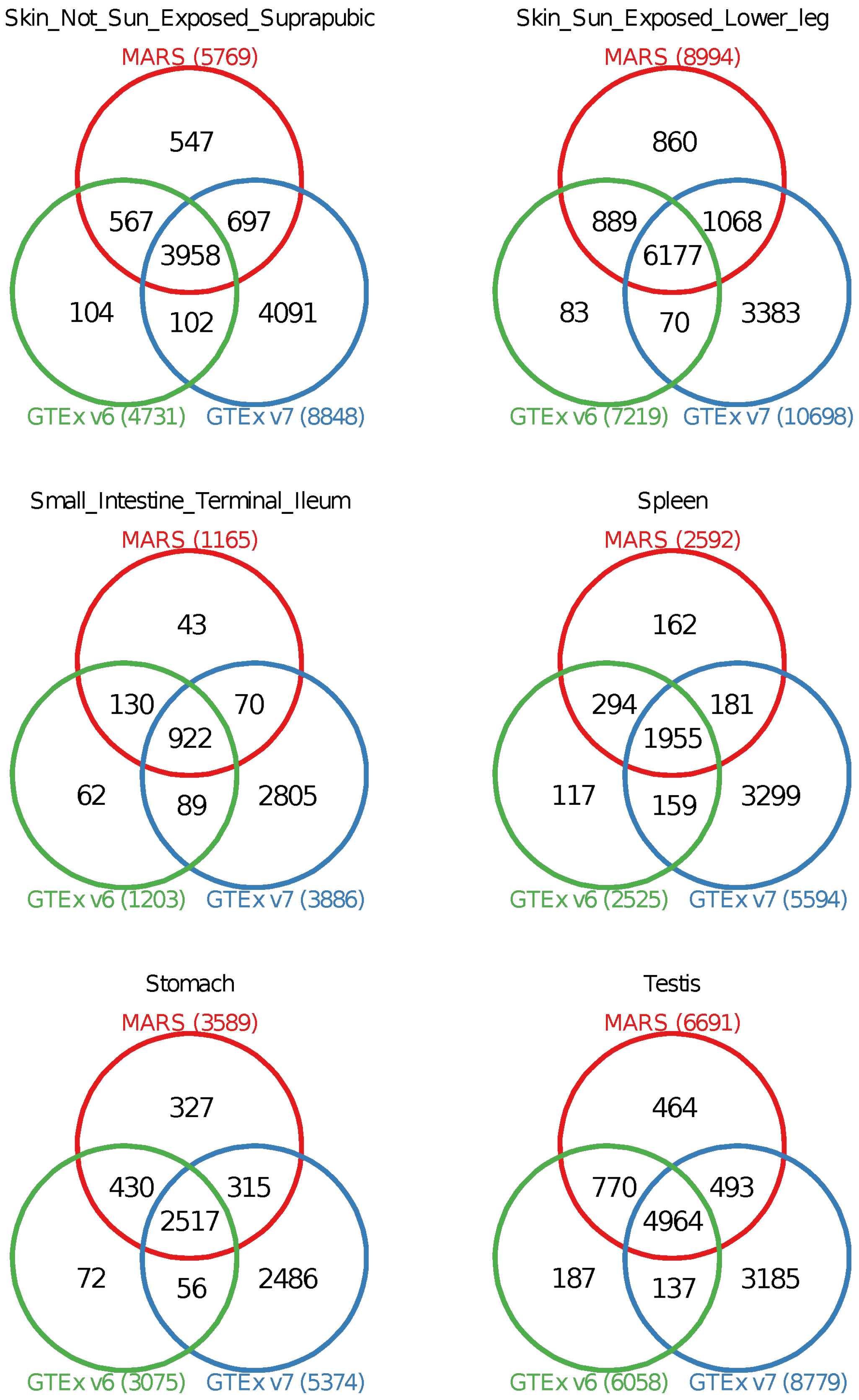
Skin_Not_Sun_Exposed_Suprapubic
MARS (5769)
547
567
697
3958
104
102
4091
GTEx v6 (4731)
GTEx v7 (8848)
Skin_Sun_Exposed_Lower_leg
MARS (8994)
860
889
1068
6177
83
70
3383
GTEx v6 (7219)
GTEx v7 (10698)
Small_Intestine_Terminal_Ileum
MARS (1165)
43
130
70
922
62
89
2805
GTEx v6 (1203)
GTEx v7 (3886)
Spleen
MARS (2592)
162
294
181
1955
117
159
3299
GTEx v6 (2525)
GTEx v7 (5594)
Stomach
MARS (3589)
327
430
315
2517
72
56
2486
GTEx v6 (3075)
GTEx v7 (5374)
Testis
MARS (6691)
464
770
493
4964
187
137
3185
GTEx v6 (6058)
GTEx v7 (8779)

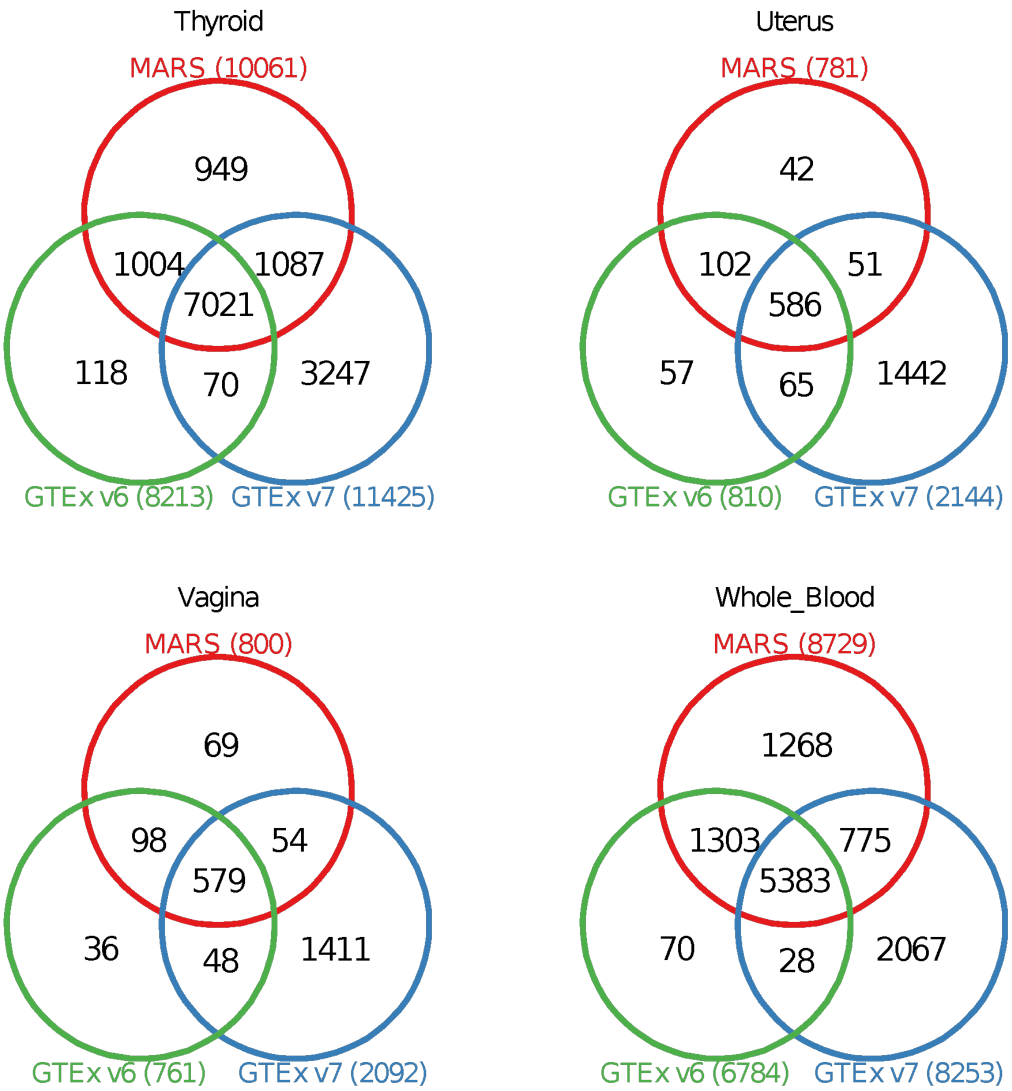
Thyroid
MARS (10061)
949
1004
1087
7021
118
70
3247
GTEx v6 (8213)
GTEx v7 (11425)
Uterus
MARS (781)
42
102
51
586
57
65
1442
GTEx v6 (810)
GTEx v7 (2144)
Vagina
MARS (800)
69
98
54
579
36
48
1411
GTEx v6 (761)
GTEx v7 (2092)
Whole_Blood
MARS (8729)
1268
1303
775
5383
70
28
2067
GTEx v6 (6784)
GTEx v7 (8253)

Fig. S4. Compare *p*-values estimated from the standard sampling and those estimated from the importance sampling.

To examine the performance of importance sampling that we proposed in the paper, we compared the *p*-values estimated from 10^8 number of standard sampling and those estimated from 10^4 and 10^5 number of importance sampling. 10 randomly selected genes in GTEx data were used for the experiments. For the case of pvalue =0, we set the $-\log_{10}$(*p*-value) as 8.

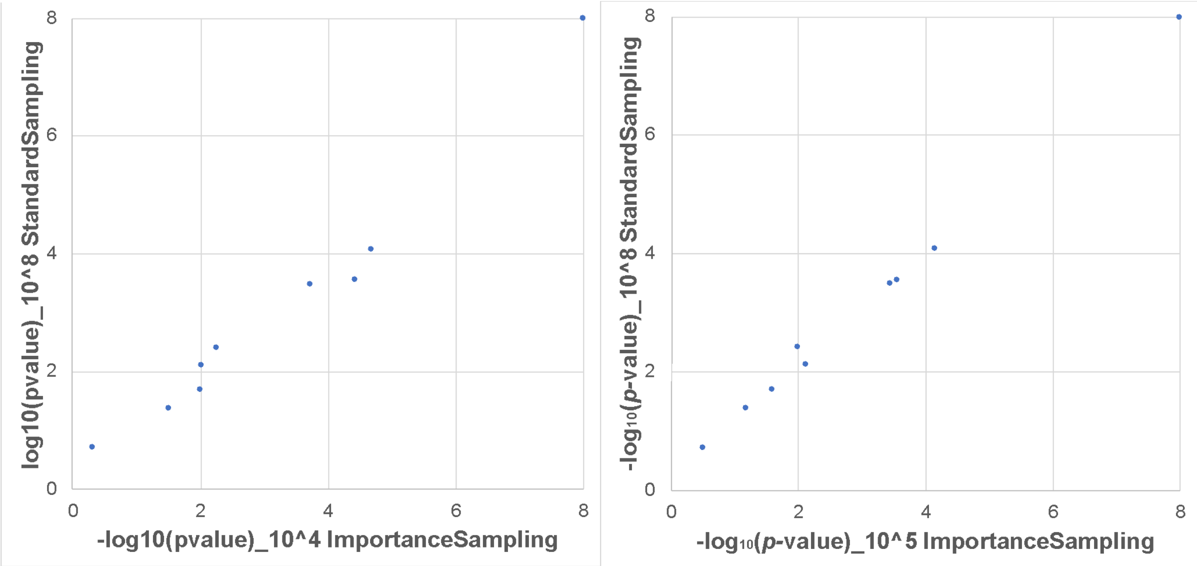

Fig. S4. The x-axis shows the $-\log_{10}$(*p*-value) estimated from 10^4 (the left panel) and 10^5 (the right panel) number of importance sampling. The y-axis shows the $-\log_{10}$(*p*-value) estimated from 10^8 number of standard sampling.

Fig. S5. Venn diagrams comparing set-based association identified by MARS and the univariate test for traits in NFBC data.

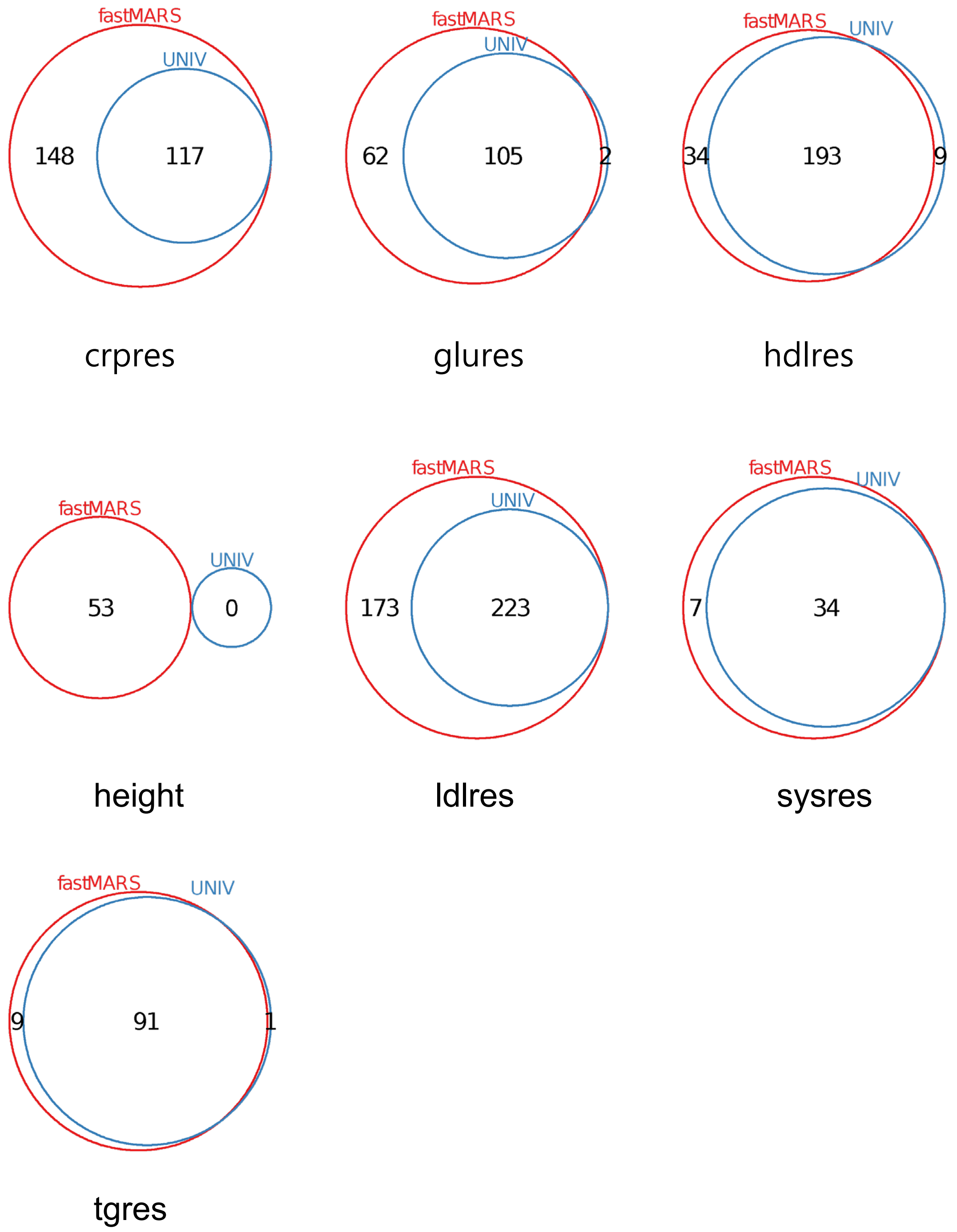

Fig. S5. The red circles show the gene-based associations identified by MARS and the blue circles show those identified by the univariate test for traits, crpres, glures, hdlres, height, ldlres, sysres, and tgres.

Fig. S6. Number of loci found by previous studies among the loci identified by MARS but not by the univariate test.

In 5 NFBC traits including C-reactive protein (CRP), Height, High-density lipoprotein (HDL), Low-density lipoprotein (LDL), and Triglycerides (TG), 471 loci were identified only by MARS. To verify those extra loci, we searched the loci from other GWAS utilizing GWAS catalog and found several variants associated with 311 loci among those loci [2–29]. For example, rs6060369 locus associated with height was identified previous large GWAS [7,22,25]. In HDL traits, rs1800961 locus was found previous large or meta-analysis GWAS studies [4–6,10,29] . rs6511720 locus related to LDL was discovered previous several studies [4,6,10,18,27,28]. The table below show the 331 overlapped loci. For example, in the case of Height, 53 loci are identified only by MARS but not by the univariate test and for those 53 loci, 12 SNPs were reported by GWAS catalog. Note that loci are defined based on the gene map of GTEx (+/- 1Mb of TSS), thus some loci may overlap and in the case one SNP may match to several loci.

Trait	Number of loci identified only by MARS but not by the univariate test	Number of associated variants reported by GWAS catalog for the corresponding loci
C-reactive protein (CRP)	99 loci	1 SNPs
Height	53 loci	12 SNPs
High-density lipoprotein (HDL)	33 loci	28 SNPs
Low-density lipoprotein (LDL)	125 loci	19 SNPs
Triglycerides (TG)	1 locus	6 SNPs
total	311 loci	66 SNPs

Overlapped loci between GWAS catalog and 471 extra loci that are identified only by MARS but not by the univariate test.

Fig. S7. Power of MARS on the choice of priors on gamma.

To clarify the sensitivity of the gamma on the result, we have performed an experiment using 10^5 simulations and a threshold of 10^{-5}. We compared the power of MARS with a range of gamma priors (γ = 0.0001,0.001, 0.01, 0.1, 0.5) for different effect sizes (λ = 2, 2.5, 3, 3.5, 4, 4.5, 5, 5.5, 6). For all the effect sizes, the choice of gamma did not show big differences on the results.

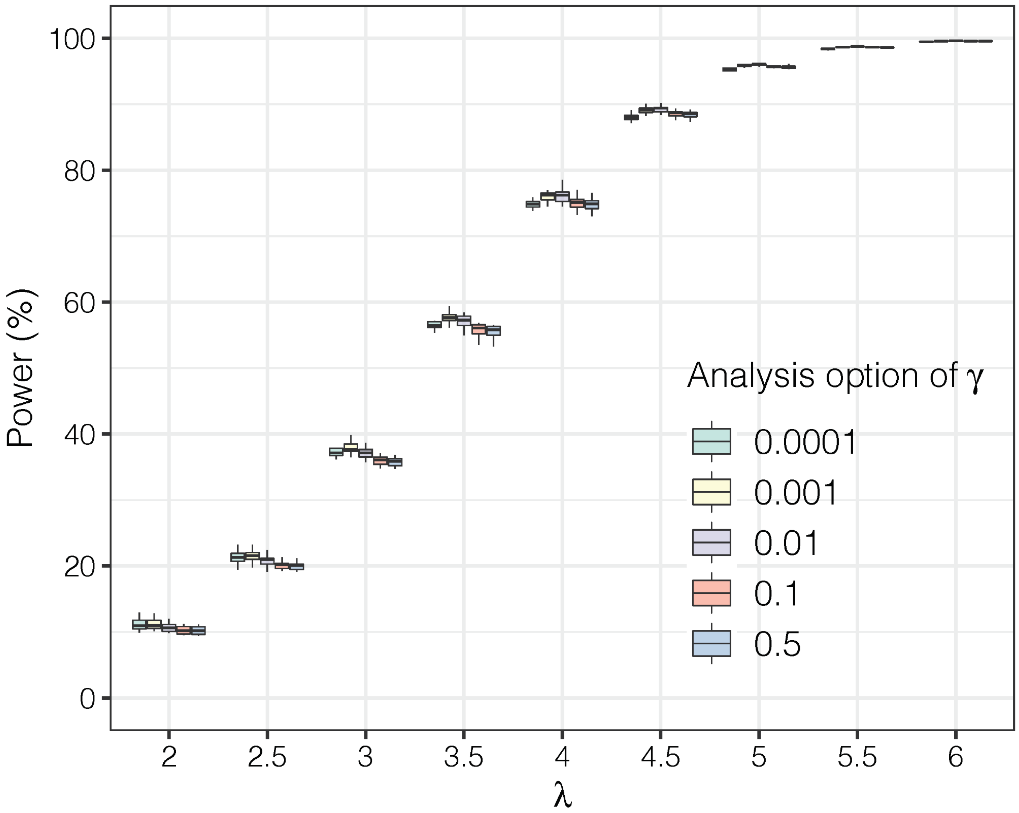

Fig. S7. Box plot showing the power of MARS based on gamma prior. The X-axis shows the effect sizes of λ = 2, 2.5, 3, 3.5, 4, 4.5, 5, 5.5, and 6 used for the test. The Y-axis shows the power in percentages. The boxplots show the power of MARS based on gamma prior (γ = 0.0001, 0.001, 0.01, 0.1, 0.5).

Fig. S8. Using only top 50 SNPs with bigger summary statistics well approximates the result using all the SNPs in MARS.

To estimate the LRT stats, we need to examine 2^m causal statuses for a locus with m SNPs. As this is computationally expensive, we order the SNPs by their values of summary statistics and use only top 50 SNPs with bigger summary statistics because we expect the causal SNPs will be included in the top 50 SNPs. To validate this approximation works in practice, we examined 10 genes of different sizes (< 500 SNPs) and computed *p*-values using MARS. We performed 10000 simulations by sampling null summary statistics from MVN distribution to access the significance of the genes. We compare *p*-values estimated from all the SNPs and those estimated from only top 50 SNPs. The figure below shows that for some cases using all the SNPs results slightly better *p*-value but in general *p*-values from all the SNPs and those from top 50 are close enough that using only top 50 SNPs can well approximate the results using all the SNPs, while reduce the time complexity dramatically.

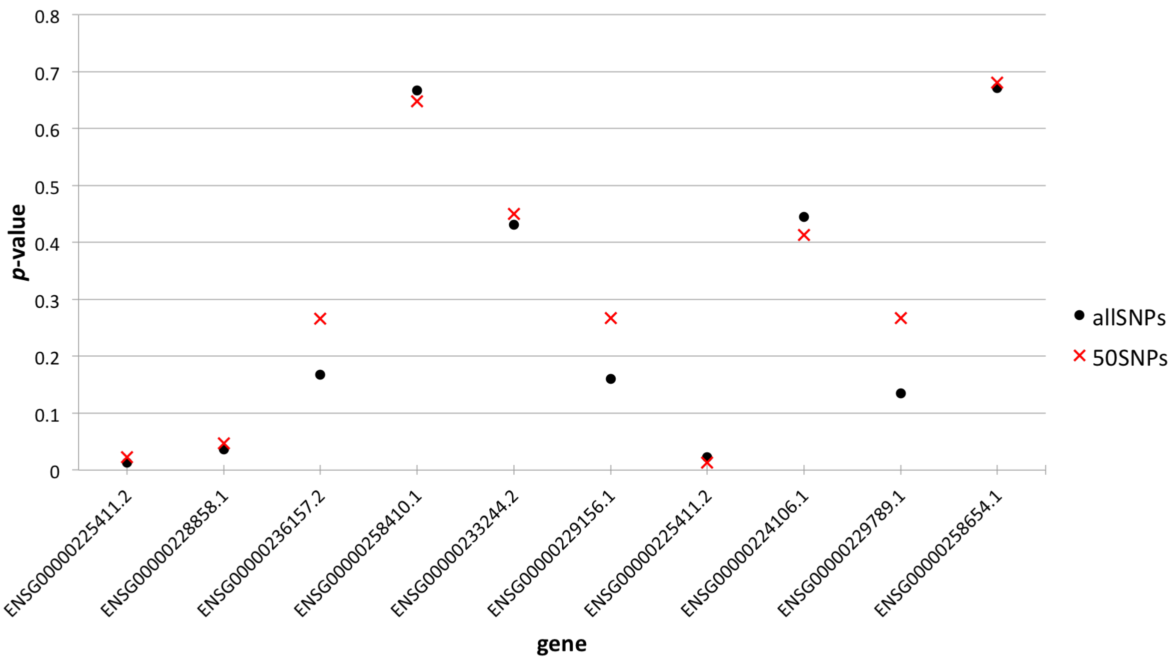

Fig. S8. Comparison of *p*-values estimated from all the SNPs and *p*-values estimated from top 50 SNPs for different genes. The x-axis shows the genes and the y-axis shows the *p*-values. Black circles show the *p*-values estimated from all the SNPs and red crosses show *p*-values estimated from top 50 SNPs.

Reference

1. Lee S, Teslovich TM, Boehnke M, Lin X. General framework for meta-analysis of rare variants in sequencing association studies. Am J Hum Genet. 2013;93:42–53.

2. Okada Y, Kamatani Y, Takahashi A, Matsuda K, Hosono N, Ohmiya H, et al. A genome-wide association study in 19 633 Japanese subjects identified LHX3-QSOX2 and IGF1 as adult height loci. Hum Mol Genet. 2010;19:2303–12.

3. Spracklen CN, Chen P, Kim YJ, Wang X, Cai H, Li S, et al. Association analyses of East Asian individuals and trans-ancestry analyses with European individuals reveal new loci associated with cholesterol and triglyceride levels. Hum Mol Genet. 2017;26:1770–84.

4. Teslovich TM, Musunuru K, Smith AV, Edmondson AC, Stylianou IM, Koseki M, et al. Biological, clinical and population relevance of 95 loci for blood lipids. Nature. 2010;466:707–13.

5. Ligthart S, Vaez A, Hsu Y-H, Inflammation Working Group of the CHARGE Consortium, PMI-WG-XCP, LifeLines Cohort Study, et al. Bivariate genome-wide association study identifies novel pleiotropic loci for lipids and inflammation. BMC Genomics. 2016;17:443.

6. Kathiresan S, Willer CJ, Peloso GM, Demissie S, Musunuru K, Schadt EE, et al. Common variants at 30 loci contribute to polygenic dyslipidemia. Nat Genet. 2009;41:56–65.

7. Sanna S, Jackson AU, Nagaraja R, Willer CJ, Chen W-M, Bonnycastle LL, et al. Common variants in the GDF5-UQCC region are associated with variation in human height. Nat Genet. 2008;40:198–203.

8. Davis JP, Huyghe JR, Locke AE, Jackson AU, Sim X, Stringham HM, et al. Common, low-frequency, and rare genetic variants associated with lipoprotein subclasses and triglyceride measures in Finnish men from the METSIM study. PLoS Genet. 2017;13:e1007079.

9. Wood AR, Esko T, Yang J, Vedantam S, Pers TH, Gustafsson S, et al. Defining the role of common variation in the genomic and biological architecture of adult human height. Nat Genet. 2014;46:1173–86.

10. Willer CJ, Schmidt EM, Sengupta S, Peloso GM, Gustafsson S, Kanoni S, et al. Discovery and refinement of loci associated with lipid levels. Nat Genet. 2013;45:1274–83.

11. Nagy R, Boutin TS, Marten J, Huffman JE, Kerr SM, Campbell A, et al. Exploration of haplotype research consortium imputation for genome-wide association studies in 20,032 Generation Scotland participants. Genome Med. 2017;9:23.

12. Kanai M, Akiyama M, Takahashi A, Matoba N, Momozawa Y, Ikeda M, et al. Genetic analysis of quantitative traits in the Japanese population links cell types to complex human diseases. Nat Genet. 2018;50:390–400.

13. Chasman DI, Giulianini F, MacFadyen J, Barratt BJ, Nyberg F, Ridker PM. Genetic determinants of statin-induced low-density lipoprotein cholesterol reduction: the Justification for the Use of Statins in Prevention: an Intervention Trial Evaluating Rosuvastatin (JUPITER) trial. Circ Cardiovasc Genet. 2012;5:257–64.

14. Lu X, Huang J, Mo Z, He J, Wang L, Yang X, et al. Genetic Susceptibility to Lipid Levels and Lipid Change Over Time and Risk of Incident Hyperlipidemia in Chinese Populations. Circ Cardiovasc Genet. 2016;9:37–44.

15. Waterworth DM, Ricketts SL, Song K, Chen L, Zhao JH, Ripatti S, et al. Genetic variants influencing circulating lipid levels and risk of coronary artery disease. Arterioscler Thromb Vasc Biol. 2010;30:2264–76.

16. Weedon MN, Lango H, Lindgren CM, Wallace C, Evans DM, Mangino M, et al. Genome-wide association analysis identifies 20 loci that influence adult height. Nat Genet. 2008;40:575–83.

17. Sabatti C, Service SK, Hartikainen A-L, Pouta A, Ripatti S, Brodsky J, et al. Genome-wide association analysis of metabolic traits in a birth cohort from a founder population. Nat Genet. 2009;41:35–46.

18. Lettre G, Palmer CD, Young T, Ejebe KG, Allayee H, Benjamin EJ, et al. Genome-wide association study of coronary heart disease and its risk factors in 8,090 African Americans: the NHLBI CARe Project. PLoS Genet. 2011;7:e1001300.

19. Coram MA, Duan Q, Hoffmann TJ, Thornton T, Knowles JW, Johnson NA, et al. Genome-wide characterization of shared and distinct genetic components that influence blood lipid levels in ethnically diverse human populations. Am J Hum Genet. 2013;92:904–16.

20. Berndt SI, Gustafsson S, Mägi R, Ganna A, Wheeler E, Feitosa MF, et al. Genome-wide meta-analysis identifies 11 new loci for anthropometric traits and provides insights into genetic architecture. Nat Genet. 2013;45:501–12.

21. Lango Allen H, Estrada K, Lettre G, Berndt SI, Weedon MN, Rivadeneira F, et al. Hundreds of variants clustered in genomic loci and biological pathways affect human height. Nature. 2010;467:832–8.

22. Lettre G, Jackson AU, Gieger C, Schumacher FR, Berndt SI, Sanna S, et al. Identification of ten loci associated with height highlights new biological pathways in human growth. Nat Genet. 2008;40:584–91.

23. Aulchenko YS, Ripatti S, Lindqvist I, Boomsma D, Heid IM, Pramstaller PP, et al. Loci influencing lipid levels and coronary heart disease risk in 16 European population cohorts. Nat Genet. 2009;41:47–55.

24. Gudbjartsson DF, Walters GB, Thorleifsson G, Stefansson H, Halldorsson BV, Zusmanovich P, et al. Many sequence variants affecting diversity of adult human height. Nat Genet. 2008;40:609–15.

25. He M, Xu M, Zhang B, Liang J, Chen P, Lee J-Y, et al. Meta-analysis of genome-wide association studies of adult height in East Asians identifies 17 novel loci. Hum Mol Genet. 2015;24:1791–800.

26. Soranzo N, Rivadeneira F, Chinappen-Horsley U, Malkina I, Richards JB, Hammond N, et al. Meta-analysis of genome-wide scans for human adult stature identifies novel Loci and associations with measures of skeletal frame size. PLoS Genet. 2009;5:e1000445.

27. Willer CJ, Sanna S, Jackson AU, Scuteri A, Bonnycastle LL, Clarke R, et al. Newly identified loci that influence lipid concentrations and risk of coronary artery disease. Nat Genet. 2008;40:161–9.

28. Kathiresan S, Melander O, Guiducci C, Surti A, Burtt NP, Rieder MJ, et al. Six new loci associated with blood low-density lipoprotein cholesterol, high-density lipoprotein cholesterol or triglycerides in humans. Nat Genet. 2008;40:189–97.

29. Surakka I, Horikoshi M, Mägi R, Sarin A-P, Mahajan A, Lagou V, et al. The impact of low-frequency and rare variants on lipid levels. Nat Genet. 2015;47:589–97.
