# Supplementary material for: MARS: leveraging allelic heterogeneity to increase power of association testing
Source: Genome Biol. 2021 Apr 30;22:128. doi: 10.1186/s13059-021-02353-8 (PMC8086090; doi:10.1186/s13059-021-02353-8)
Supplement: Supplementary file 6 — Additional file 6 Review history [file 13059_2021_2353_MOESM6_ESM.docx]

Review History

**First round of review**

**Reviewer 1**

**Were you able to assess all statistics in the manuscript, including the appropriateness of statistical tests used?**

Yes: This paper is about statistical methodology, and I mainly assessed the statistical procedures that are proposed.

**Were you able to directly test the methods?**

No

**Comments to author:**

This paper presents a new approach for quantifying the association between a set of SNPs and a phenotype utilizing summary level statistics. The model is built on the CAVIAR method which tries to identify SNPs with high causal probability. Improvements on power has been shown in both simulations and in GTEx dataset. My major and minor concerns are as follows:

Major concerns:

The approach how statistical significance is assessed is unclear to me. What does it men by "we find a LRT threshold that corresponds to the p-value threshold of 5 ×〖10〗^(-8)"? , also, "The p-value threshold to identify eGenes is defined as the border of empirical p-values of eGene and non-eGene reported by GTEx consortium"? Are they the same threshold?

Further, it seems that LRT^Data is calculated from the top 50 SNPs due to consideration of computational capacity, but the LRT^NULL is calculated from all SNPs? (page 20 and 21). Would not this cause any problem?

The type I errors were assessed only at alpha = 0.05, 0.01 and 0.1 levels. In GWAS studies, we should always assess the significance at much smaller alpha levels.

From Figure 2, it seems that the power from the univariate test approaches the MARS when the underlying causal SNP has a large effect size, which is totally understandable and expected. The DAP-G and fastBAT methods approaches MARS when the causal effect is small. I would envision that DAP-G and fastBAT could become more advantageous when there are even more % of causal SNPs.

Some discussions on this would be helpful. This doesn't support the claim that "we show that when there are many causal variants with small effect sizes".

SKAT does have a meta-analysis version, which could also be compared with in the paper in simulations.

Most GWAS are case-control studies. It seems from the method section that the model relies on linear regressions. Would the model be applicable when the outcome is binary? Is there any modification needed?

Minor issues:

Although known to most readers, allelic heterogeneity is not defined, and why it is important is not apparent.

In Figure 4, MARS seems to detect a large number of new eGenes in the tissues that already reported a large number of eGenes (maybe because of sample sizes or the tissue specific effect). But in tissues there were small number of eGenes discovered in GTEx, the improvement is small. What does this indicate? Any suggestions on further analysis?

Technically speaking, the test statistics is not a likelihood ratio test because LRT = Likelihood (full model)/ Likelihood (null model). However, the statistic that the paper uses is Likelihood (alternative model)/ Likelihood (null model). Although this would not impact the validity of the model as a resampling method is used for hypothesis testing. But it would be nice to notice this. Further, it is seldomly denoted as LRT_score because usually score test is another big school of test in statistics, and such a notation would confuse readers.

How is the gamma prior chosen (page 19)? Is the model sensitive to the choice of this?

**Reviewer 2**

**Were you able to assess all statistics in the manuscript, including the appropriateness of statistical tests used?**

Yes: I almost answered no given the poor writing.

**Were you able to directly test the methods?**

This is a potentially interesting paper that might result in more powerful GWAS. Unfortunately, the writing is so unclear to consistently distract the reader from its message and at times to make that message unclear. I gave up on documenting the myriad examples of poor writing and instead focus only on truly important ambiguities along with relevant issues of science. I urge the authors to do a careful review of all aspects of the manuscript (well beyond my comments here), engaging an editor if necessary.

1. Please state early on:

a. Which summary statistics are required for the methods

b. That you routinely assume <4 causal variants in a region and that computation becomes (challenging? Impractical?) for larger numbers of causal variants

c. That the method is limited to common variants

d. Whether inverse normalization of the phenotype data is required

e. Whether the method can deal with related individuals

f. The method is limited to genes (if this is true)

2. The set of methods to which you compare MARS is inconsistent, for example, between the GTEx and NFBC data. Please be consistent or explain why you are not.

3. The authors assume without discussion that a reference dataset is available to allow accurate estimation of covariances. Attention needs to be paid to this assumption, either justifying it or admitting that it is a limitation that requires further investigation.

4. The authors state that computation is substantially simplified by being able to re-use determinations of significance thresholds. What assumptions are made here? In particular, what must remain exactly or approximately the same? How does MARS deal with missing genotype data in this context and more generally?

5. Since "the model of CAVIAR" is an important component of the method, that model should be defined or at least outlined in the main text.

6. I do not understand the importance sampling procedure that allows pvalues of 5x10-8 to be accurately estimated by 10000 simulation replicates. Also unclear is how limiting attention to 50 variants in a large region is acceptable.

7. In what sense are alpha values of .10, .05, and .01 interesting? (p. 6)

8. How large are effect sizes lambda of 4 to 6? And is lambda being used to represent three different variables over the course of the paper?

9. Showing something true for 2 or 3 causal variants does not seem like a completely general proof. (p. 7)

10. The use of GTEx v6 and v7 makes sense. The inclusion of FHS is confusing. It would be helpful to give numbers of "additional loci" actually do look correct rather than characterizing this as "some".

**Comments to author:**

**Authors Response**

**Point-by-point responses to the reviewers’ comments:**

Reviewer 1 Remarks to the Author:

This paper presents a new approach for quantifying the association between a set of SNPs and a phenotype utilizing summary level statistics. The model is built on the CAVIAR method which tries to identify SNPs with high causal probability. Improvements on power has been shown in both simulations and in GTEx dataset. My major and minor concerns are as follows:

Reviewer 1 Major Comment #1:

The approach how statistical significance is assessed is unclear to me. What does it mean by "we find a LRT threshold that corresponds to the p-value threshold of 5e-08"?, also, "The p-value threshold to identify eGenes is defined as the border of empirical p-values of eGene and non-eGene reported by GTEx consortium"? Are they the same threshold?

*Author Response:*

*Thank you very much for the comment, and we totally agree that the ways we set the thresholds are very confusing for the readers. We made changes in the manuscript clarifying this.*

*For the first comment "we find a LRT threshold that corresponds to the p-value threshold of 5e-08"*

*To compare MARS with a standard approach of set-based association test, we define a univariate test that uses a maximum summary statistic among the SNPs in the analysis locus. Let’s say we are testing for a region with m SNPs. For the GWAS, we generated a 10⁸ null panel of m statistics, which we sample from MVN with no effect. For the univariate test, we used the maximum p-value, Pmax, as the set-based score, and for MARS, we computed LRT stat, LRTstat, for the set-based score. Thus, we have 10⁸ Pmax scores and 10⁸ LRTstat. Now we ordered the Pmax scores and found the quantile, q, where the Pmax equals (approximates) 5e-08. Then we ordered the LRTstat and find the specific LRTstat, LRTstatq, that corresponds to the quantile q. That LRTstatq becomes the threshold for the MARS. We have described this process in detail in the Materials and Method, “Power estimation” and “The standard univariate test, fastBAT, and DAP-G”. However, we completely agree this was very confusing for readers reading the Results section; thus, we clarified the sentence in the Results section, “MARS controls type I error while improving power in simulation studies” as follows (page 7, ln 155-162).*

*“To set a threshold for computing the power, we utilized the concept of the univariate test (see Materials and Methods, The standard univariate test, fastBAT, and DAP-G). For each null statistic, we selected the maximum p-value to get 10⁸ maximum p-values from the null panel. Then we ordered the maximum p-values to get the quantile, q, where the maximum p-values corresponds to 5e-08; q is used for setting the LRT thresholds. Then the power is estimated as a quantile of alternative cases that show LRTstat greater than the LRT threshold. The details of the whole processes of computing the threshold and estimated the power is described in Materials and Method, Power estimation.”*

*For the second comment, "The p-value threshold to identify eGenes is defined as the border of empirical p-values of eGene and non-eGene reported by GTEx consortium."*

*To compare the MARS results with GTEx's results, we used 10⁴ number of simulations unlike the GWAS or simulations studies. This is because it is the number of simulations used by GTEx consortium to compute `empirical p-values' to select eGenes. For the fair comparison, we set the threshold as the border of empirical p-value between eGenes and genes other than those eGenes, referred to as non-eGenes, to identify eGenes for MARS. We clarified the sentence in the Results section, “MARS detects novel eGenes in GTEx data” as below (page 10, ln 195-201).*

*“For simulation studies and GWAS, we applied a threshold that corresponds to the p-values threshold of 5e-08 utilizing the univariate test. However, for a fair comparison of MARS results with those reported by the GTEx consortium, a different strategy has been used. We used 10,000 simulations, which is the number of simulations used by the GTEx consortium to compute their “empirical p-values” and select eGenes. To identify eGenes for MARS, we set the threshold as the border of empirical p-values between eGenes and other genes, referred to as non-eGenes, reported by the GTEx consortium.”*

*In addition, we clarify this in the section in Materials and Methods, “eGene detection in GTEx data” (page 21-22, ln 428-432).*

*“To compare the MARS results with GTEx's results, we use 10⁸ simulations, the number used by GTEx Consortium to compute their `empirical p-values' to select eGenes. To identify eGenes for MARS, we set the threshold as the border of empirical p-value between eGenes and genes other than those eGenes, referred to as non-eGenes, reported by the GTEx consortium, which is differ by tissues as GTEx used the FDR approach to find their eGenes.”*

Reviewer 1 Major Comment #2:

Further, it seems that LRT^Data is calculated from the top 50 SNPs due to consideration of computational capacity, but the LRT^NULL is calculated from all SNPs? (page 20 and 21). Would not this cause any problem?

*Author Response:*

*Thank you very much for pointing this out. Both LRT^DATA and LRT^NULL use the same strategy and calculated from the top 50 SNP. We clarified the sentence by adding and revising page 21, ln 419-423.*

*“Based on the simulation data, we order the SNPs by values of their summary statistics and use only top 50 SNPs for computing the LRTstat; LRTstat^NULL, using Equation (6). Then, we also select the top 50 SNPs of summary statistics to compute the LRTstat of the gene; LRTstat^DATA, using Equation (6). The p-value of the gene is estimated as the quantile of LRTstat^DATA among LRTstat^NULL.”*

Reviewer 1 Major Comment #3:

The type I errors were assessed only at alpha = 0.05, 0.01 and 0.1 levels. In GWAS studies, we should always assess the significance at much smaller alpha levels.

*Author Response:*

*We agree that smaller alpha levels are appropriate and realistic to show the type I errors for genetic analysis. We replace the experiment showing smaller alpha in Figure 2 (a). Due to the time cost, we generated seven orders of simulations and showed alpha as small as 5e-06. We expect to have accurate results in smaller alpha as well with more simulations. We clarified the sentence by adding page 7, ln 143-145.*

*“Figure 2 (a) shows that MARS robustly controls type I error for all examined gene loci as the false-positive rates for different gene loci are very close to the corresponding 5e-06 to 5e-02, accordingly.”*

Reviewer 1 Major Comment #4:

From Figure 2, it seems that the power from the univariate test approaches the MARS when the underlying causal SNP has a large effect size, which is totally understandable and expected. The DAP-G and fastBAT methods approaches MARS when the causal effect is small. I would envision that DAP-G and fastBAT could become more advantageous when there are even more % of causal SNPs. Some discussions on this would be helpful. This doesn't support the claim that "we show that when there are many causal variants with small effect sizes".

*Author Response:*

*Thank you very much for the comment. We limited the number of causal variants in a locus up to three. We believe this is a reasonable assumption, as it has been reported that a relatively small number of variants exist in a region; however, this may not be a general assumption, depending on the data. We totally agree that more % of causal SNPs, DAP-G and fastBAT could show better power, which needs more investigation. We clarified the sentence in Discussion section by adding and revising the page 17, ln 330-336.*

*“Second, we limited the number of causal variants in a locus up to three in the simulated studies to reduce the running time in experiments. We believe this is a reasonable assumption, as it has been reported that a relatively small number of variants exist in a region [13] and MARS showed better results in practice, utilizing real eQTL and GWAS datasets; GTEx data and NFBC data. However, this may not be a general assumption and further investigations may require for the cases with larger number of causal variants (>3), comparing with previous methods such as DAP-G and fastBAT, which may give better results for the cases.”*

Reviewer 1 Major Comment #5:

SKAT does have a meta-analysis version, which could also be compared with in the paper in simulations.

*Author Response:*

*Thank you for this comment. We have looked at the MetaSKAT, which seems very interesting for the meta-analysis of set-based tests. However, the MetaSKAT is for combining multiple set-based tests beyond the scope of our paper as we are only aiming to analyze a single data set. We believe that MetaSKAT could be used as a post-analysis for not only SKAT, SKAT-O, and burden test, but also for MARS, which could be very interesting for our further studies. Thank you again for bringing this to our attention.*

Reviewer 1 Major Comment #6:

Most GWAS are case-control studies. It seems from the method section that the model relies on linear regressions. Would the model be applicable when the outcome is binary? Is there any modification needed?

*Author Response:*

*Thank you for the comment. We understand a large portion of GWAS are case-control studies, and our method does not require any modification for the binary data.*

Reviewer 1 Minor Comment #1:

Although known to most readers, allelic heterogeneity is not defined, and why it is important is not apparent.

*Author Response:*

*Thank you for the comment. We clarified the term of allelic heterogeneity in the Introduction section (page 3, ln 45-47).*

*“The phenomenon is known as allelic heterogeneity, which is very common in Mendelian traits [16] and recent works have demonstrated widespread allelic heterogeneity in expression quantitative trait loci (eQTLs) and complex traits [17, 18]”*

Reviewer 1 Minor Comment #2:

In Figure 4, MARS seems to detect a large number of new eGenes in the tissues that already reported a large number of eGenes (maybe because of sample sizes or the tissue specific effect). But in tissues there were small number of eGenes discovered in GTEx, the improvement is small. What does this indicate? Any suggestions on further analysis?

*Author Response:*

*MARS tries to increase the statistical power of detecting new signals of a region considering multiple variants simultaneously. We expect MARS will be advantageous over the univariate test when there are more than one causal SNPs with small effect sizes, which the univariate test cannot detect. We agree that in tissues, where the GTEx consortium reports a small number of eGenes, MARS' improvement is small. Thank you very much for pointing this out, and we find it very interesting. One of the possible scenarios for this phenomenon is that if the causal variant does not exist or is not multiple in a region, the MARS will not be advantageous over the univariate test and that improvement with the use of MARS is small for the tissues. Especially, suppose causal variants have extremely small effect sizes. In that case, even if multiple such variants exist in a region, we believe that it will be impossible to detect the region by MARS and any other methods.*

Reviewer 1 Minor Comment #3:

Technically speaking, the test statistics is not a likelihood ratio test because LRT = Likelihood (full model)/ Likelihood (null model). However, the statistic that the paper uses is Likelihood (alternative model)/ Likelihood (null model). Although this would not impact the validity of the model as a resampling method is used for hypothesis testing. But it would be nice to notice this. Further, it is seldomly denoted as LRT_score because usually score test is another big school of test in statistics, and such a notation would confuse readers.

*Author Response:*

*We entirely agree with the reviewer, which was one of the main issues we have considered throughout the writing. In the Introduction, instead of using the term “Likelihood Ratio Test”, we introduced the MARS as below. We clarified the sentence by adding and revising page 3-4, ln 63-65.*

*“MARS computes a likelihood ratio of a null model, where none of the variants are causal against an alternate model, where at least one variant is causal. Then an efficient re-sampling approach is applied for the significance test.”*

*We rewrote this in the Results section, “Overview of the MARS” and added a note to avoid readers confusing it with the standard LRT test (page 5, ln 105-109).*

*“To test the association between a set of variants of a locus and a trait, MARS estimates a likelihood ratio to compute a test statistic, which is referred to as Likelihood Ratio Test (LRT) statistic; LRTstat. We consider the likelihood of a null model (L₀) and the alternative model (L₁). Note that we are computing the likelihood ratio of a null model against the alternative model, not the full model, which is the standard form of the “Likelihood ratio test” uses.”*

*Also, we have changed the notation LRTscore to “LRTstat” throughout the manuscript. Thank you again for the great comment.*

Reviewer 1 Minor Comment #4:

How is the gamma prior chosen (page 19)? Is the model sensitive to the choice of this?

*Author Response:*

*To choose the prior of causal status (γ), we use the same assumptions that are widely used in fine-mapping methods including CAVIAR, which our primary model is based on. We clarified the gamma prior chosen by adding the Method section (page 20, ln 383-385).*

*“To compute the prior of causal status, we use the same assumptions that are widely used in fine-mapping methods, and γ is set to 0.01 [13, 77–81]. We assume each SNP is independent and that the probability of a SNP to be causal is equal to 0.01 [79-80]. Therefore, we compute the prior probability as follows: “*

Reviewer 2 Remarks to the Author:

This is a potentially interesting paper that might result in more powerful GWAS. Unfortunately, the writing is so unclear to consistently distract the reader from its message and at times to make that message unclear. I gave up on documenting the myriad examples of poor writing and instead focus only on truly important ambiguities along with relevant issues of science. I urge the authors to do a careful review of all aspects of the manuscript (well beyond my comments here), engaging an editor if necessary.

Reviewer 2 Specific Comment #1:

1. Please state early on

Reviewer 2 Specific Comment #1-a:

a. Which summary statistics are required for the methods

*Author Response:*

*Thank you very much for pointing this out. GWAS summary statistics such as Z-scores with SNP identifier are required at a minimum in the MARS analysis. We clarified this in the Introduction section (page 4, ln 67).*

*“Our method does not require individual-level data, which is often not provided in GWAS. MARS only requires summary statistics such as Z-scores and the LD of variants in a locus, which can be obtained from a reference dataset such as HapMap.”*

Reviewer 2 Specific Comment #1-b:

b. That you routinely assume <4 causal variants in a region and that computation becomes (challenging? Impractical?) for larger numbers of causal variants

*Author Response:*

*Thank you very much for pointing this out, and we find that it is important to state this point early on for users to know whether considering more variants is practical or not. We added the section “Overview of MARS” (page 5-6, ln 118-124).*

*“In practice, we limited the number of allowed causal SNPs as well as the number of variants considered for a region to reduce the running time in experiments throughout the paper. We find that considering up to 3 causal variants and use 50 variants in a region to be reasonable in respect to both the running time and the accuracy in our experiments. However, a user may increase the numbers, which is amendable in high computing servers and may provide more accurate results in expense of the running time. For details, see the Materials and Methods section.”*

*In addition, we added more details in the section “Materials and Methods” “Model-based Association test Reflecting causal Status (MARS)”, where we show how much the computational burden increases with the number of causal SNPs to be considered increases (page 21, ln 411-413)*

*“However, depending on the available computational power and size and properties of the data, the number of possible causal variants for running MARS may increase using options in the MARS program.”*

Reviewer 2 Specific Comment #1-c:

c. That the method is limited to common variants

*Author Response:*

*Thank you for the comment. The MARS is limited to common variants, as the model is assuming the common variants. We have mentioned this in the Discussion section (page 17, ln 336-337).*

*“Third, our model is based on a linear model and can only be applied to common variants, not rare variants.”*

Reviewer 2 Specific Comment #1-d:

d. Whether inverse normalization of the phenotype data is required

*Author Response:*

*Thank you for the comment. As the method is based on the linear model (page 17, ln 353), we assume the data's normality. If the data does not follow a normal distribution, techniques such as rank-based inverse normal transformation could attain better analysis. We have mentioned this in the Discussion section (page 17, ln 337-339).*

*“For data that does not follow a normal distribution, we recommend to fit the data into a normal distribution using techniques such as inverse normal transformation.”*

Reviewer 2 Specific Comment #1-e:

e. Whether the method can deal with related individuals

*Author Response:*

*Thank you for the comment. We assume that the summary statistics are corrected for population stratification. We can extend our likelihood ratio model using the CAVIAR-gene model instead of CAVIAR to consider the population stratification. We think this is a great future study and has mentioned this in the Discussion section below (page 17, ln 339-341).*

*“Also, we assume the summary statistics are corrected for population stratification. We can extend our likelihood ratio model using CAVIAR-gene [15] instead of CAVIAR to consider the population stratification in the future study. “*

Reviewer 2 Specific Comment #1-f:

f. The method is limited to genes (if this is true)

*Author Response:*

*Thank you for the comment. In the manuscript, we applied our method to genes because we wanted to compare the number of genes identified by MARS with those reported elsewhere; however, they are not limited to genes but open to any sets of SNPs defined by a user. To make it clear, we added below to the Discussion section (page 16, ln 321-323).*

*“In our experiments, we have defined each locus as a gene; however, it could be defined as any set of variants that a user wants to apply to.*

Reviewer 2 Specific Comment #2:

2. The set of methods to which you compare MARS is inconsistent, for example, between the GTEx and NFBC data. Please be consistent or explain why you are not.

*Author Response:*

*Thank you for the comment, and we understand this could be a confusing point for a user. We wanted to show our method applies to either eQTL studies or GWAS. For this reason, we used the representative eQTL and GWAS datasets, GTEx, and NFBC with sufficient numbers of samples. We explained this in the Introduction section as follows (page 4, ln 79-82).*

*“In addition, to show the performance of MARS on both eQTL studies and GWAS, we have applied MARS to representative eQTL and GWAS datasets; Genotype-Tissue Expression (GTEx) data and Northern Finland Birth Cohort (NFBC) data, accordingly.”*

Reviewer 2 Specific Comment #3:

3. The authors assume without discussion that a reference dataset is available to allow accurate estimation of covariances. Attention needs to be paid to this assumption, either justifying it or admitting that it is a limitation that requires further investigation.

*Author Response:*

*The authors thank the reviewer for the helpful comment. We understand that attention is required to assume that the reference dataset will provide an accurate estimation of LD in common. We note that LD estimated from a global reference dataset may not provide an accurate estimate for multiancestry data or the human leukocyte antigen (HLA) region due to population stratification or complex LD patterns. We clarified these points to the Discussion section (page 16, ln 300-304).*

*“One of the advantages of MARS is that it may be applicable to data with only summary statistics, utilizing LD estimated from a global reference dataset such as 1000 genome data [72]. However, special attention is required for the data from multiancestry or human leukocyte antigen (HLA) region, for which a reference dataset may not provide an accurate LD estimate due to population stratification [72] or complex LD patterns [73].”*

Reviewer 2 Specific Comment #4:

4. The authors state that computation is substantially simplified by being able to re-use determinations of significance thresholds. What assumptions are made here? In particular, what must remain exactly or approximately the same? How does MARS deal with missing genotype data in this context and more generally?

*Author Response:*

*Thank you very much for the comment, and we find this was stated vaguely in the manuscript. We clarified them revising the Discussion section below (page 16, ln 307-309).*

*“For example, in our GTEx analysis, the null panel statistics for genes were established only once and applied to all of the 44 tissues. This may reduce the running time significantly as most of the running burden comes from the null panel generation.*

Reviewer 2 Specific Comment #5:

5. Since "the model of CAVIAR" is an important component of the method, that model should be defined or at least outlined in the main text.

*Author Response:*

*Thank you for the comment, and we understand that the CAVIAR is an important component of the method. To avoid confusion, we outlined the CAVIAR in the main section Introduction as below (page 3, ln 58-62). Besides, we gave details in the Materials and Methods as a whole section, “CAVIAR Generative Model” (page 19).*

*“CAVIAR [13] is a recent fine-mapping approach that estimates the probability of each variant being causal, thus allowing an arbitrary number of causal variants by jointly modeling the association statistics at all variants. We extend the likelihood model of CAVIAR to explicitly incorporate the Linkage Disequilibrium (LD) structure of data utilizing multivariate normal (MVN) distribution conditional on the causal status of the variants.”*

Reviewer 2 Specific Comment #6:

6. I do not understand the importance sampling procedure that allows pvalues of 5e-08 to be accurately estimated by 10000 simulation replicates. Also unclear is how limiting attention to 50 variants in a large region is acceptable.

*Author Response:*

*Thank you very much for the comment. We understand the concept of importance sampling is quite complicated that it may not clear to readers how we could dramatically reduce the number of sampling. We have added a reference to the section “Fast and space-efficient sampling for MARS” (page 23-24, ln 475-479), which can help readers who want to understand the procedure in detail.*

*“Thus, for GWAS, we have applied importance sampling, which is an approximation method of standard sampling. The main idea of importance sampling is that it draws the sample from a distribution with thicker tails than a target distribution. Then it uses importance weights so that the correct distribution is targeted [39]. The procedure is as follows.”*

*We have tried to describe the importance sampling procedure in more detail” with the Figure 6.*

*For the first comment," Also unclear is how limiting attention to 50 variants in a large region is acceptable."*

*We were trying to find the combination of causal variants among the 50 variants. We believe this is a reasonable assumption as we do not expect that more than 50 variants affect a region. It has been reported that a relatively small number of variants exist in a region. While including more variants in the test will give more accurate results, we believe top-50 statistics are good enough considering both the efficiency and accuracy of the method. We have tested this strategy extensively on both simulated and real datasets, and they showed good results in practice. We have mentioned this in the section “Overview of MARS” in Results (page 6, ln 120-122)*

*“We find that considering up to 3 causal variants and use 50 variants in a region to be reasonable in respect to both the running time and the accuracy in our experiments.”*

*We also have mentioned this in the section Discussion (page 17, ln 330-333)*

*“Second, we limited the number of causal variants in a locus up to three in the simulated studies to reduce the running time in experiments. We believe this is a reasonable assumption, as it has been reported that a relatively small number of variants exist in a region [13] as well as MARS showed better results in practice, utilizing real eQTL and GWAS datasets; GTEx data and NFBC data.”*

Reviewer 2 Specific Comment #7:

7. In what sense are alpha values of .10, .05, and .01 interesting? (p. 6)

*Author Response:*

*We agree that smaller alpha levels are appropriate and realistic to show the type I errors for genetic analysis. We replace the experiment showing smaller alpha in Figure 2 (a). Due to the time cost, we generated seven orders of simulations and showed alpha as small as 5e-06. We expect to have accurate results in smaller alpha as well with more simulations. We clarified the sentence by adding page 7, ln 143-145.*

*“Figure 2 (a) shows that MARS robustly controls type I error for all examined gene loci as the false-positive rates for different gene loci are very close to the corresponding 5e-06 to 5e-02, accordingly.”*

Reviewer 2 Specific Comment #8:

8. How large are effect sizes lambda of 4 to 6? And is lambda being used to represent three different variables over the course of the paper?

*Author Response:*

*Thank you very much for raising this matter. To generate the simulated data, we sampled statistics from multivariate normal distribution S^ALT~N(ΣΛ,Σ). Here, Λ is a vector of length m, where m is the number of SNPs that contain zeros except for the causal SNPs. For example, for a simulation in which two SNPs (e.g., SNP 1 and SNP 2) with effect size λ are implanted in the data, Λ is [λ,λ,0,..,0], and we tried to compare the power in a reasonable range. We agree that with just the number 4 to 6, it is very hard to infer the actual size of the effects. Unfortunately, it is very hard to define the effect size mathematically in terms of lambda. However, in each lambda case, we believe that readers can infer the size of an effect from the power itself because the power indicates how much of the true signals could be detected.*

Reviewer 2 Specific Comment #9:

9. Showing something true for 2 or 3 causal variants does not seem like a completely general proof. (p. 7)

*Author Response:*

*Thank you very much for the comment. We agree that showing results of 2 or 3 causal variants may not be sufficient depending on the data. In the article, we discussed this as a limitation in the Discussion section (page 17, ln 330-336).*

*“Second, we limited the number of causal variants in a locus up to three in the simulated studies to reduce the running time in experiments. We believe this is a reasonable assumption, as it has been reported that a relatively small number of variants exist in a region [13] and MARS showed better results in practice, utilizing real eQTL and GWAS datasets; GTEx data and NFBC data. However, this may not be a general assumption and further investigations may require for the cases with larger number of causal variants (>3), comparing with previous methods such as DAP-G and fastBAT, which may give better results for the cases.”*

Reviewer 2 Specific Comment #10:

10. The use of GTEx v6 and v7 makes sense. The inclusion of FHS is confusing. It would be helpful to give numbers of "additional loci" actually do look correct rather than characterizing this as "some".

*Author Response:*

*Thank you very much for this comment. We totally understand that readers can be confused, as using different loci may give different results. In the*

*section “MARS detects novel eGenes in GTEx data”, we focus on the validation of MARS results to show that novel eGenes (referred to as extra eGenes in the article). The eGenes are identified only by MARS but not reported by GTEx consortium using GTEx v6 data are reliable, not just false positives. Thus, we want to validate those eGenes utilizing other bigger studies that may have higher power with more sample size, such as Framingham Heart Study (FHS) that used the Whole Blood data on a large number (5,257) of samples. In this case, we believe the number of loci available in either GTEx data or the FHS data is not important because we aim to validate the novel eGenes from MARS using FHS data, not comparing the identifications.*

*We clarified this by adding the “MARS detects novel eGenes in GTEx data” section in Results (page 10, ln 205-212).*

*“To verify that the eGenes identified by MARS are true associations, we compared the extra eGenes with those reported by other studies that have larger sample sizes. Note that the results throughout the paper used data from GTEx version 6. GTEx version 7 has recently been published with more samples and improved technology in experiments. We expect more eGenes are detected in the newer version of the data as the power increases with the number of samples and etc. We compared the extra eGenes with those reported by GTEx version 7. In addition, we utilized a Whole Blood data of Framingham Heart Study (FHS) [30], which is independent of GTEx data but contains a larger sample size (5,257 samples), to validate the extra eGenes.”*

**Second round of review**

**Reviewer 1**

The revised manuscript is significantly improved, especially with respect to clarity. However, a few points are still confusing that needs some further address.

1. The MARS data is proposed as a linear additive model. When the outcome is case/control status, would the model need any change? In the previous response, the author states that there is no need for any change, which is strange. the model assumes that the residuals are Guassian distributed, which will be violated when outcome is 0/1 variable.

2. Is the model sensitive to the choices of priors on Gamma?

3. metaSKAT was proposed for meta-analysis, therefore, indeed it is designed for multiple studies. However, it should also be able to be applied when there is only a single study, with summary statistics for each SNP available instead of the genotypes. Any thought to compare your method to such a version of metaSKAT.

**Authors Response**

**Point-by-point responses to the reviewers’ comments:**

Reviewer 1 Remarks to the Author:

The revised manuscript is significantly improved, especially with respect to clarity. However, a few points are still confusing that needs some further address.

Reviewer 1 Minor Comment #1:

1. The MARS data is proposed as a linear additive model. When the outcome is case/control status, would the model need any change? In the previous response, the author states that there is no need for any change, which is strange. the model assumes that the residuals are Guassian distributed, which will be violated when outcome is 0/1 variable.

*Author Response:*

*Thank you very much for the comment. We agree with the reviewer that in the method section, we assume that phenotypic values are continuous value. However, this assumption is to ease some of mathematical derivations of computing the marginal (summary) statistics. MARS only require the joint distribution of summary statistics to follow multivariate normal distribution. It is shown in our previous works [1-3] that for binary phenotypes the joint distribution of summary statistics follows a multivariate normal distribution. Thus, as MARS only requires summary statistics and LD, we can provide marginal statistics obtained from case/control phenotypes. To make this clear, we added this description to the beginning of the Methods section.*

Reviewer 1 Minor Comment #2:

2. Is the model sensitive to the choices of priors on Gamma?

*Author Response:*

*We agree that the readers may be curious about the model sensitivity to the choices of priors on gamma. To clarify the sensitivity of the gamma on the result, we have performed an experiment using 10^5 simulations and a threshold of 10^-5. We compared the power of MARS with a range of gamma priors (γ = 0.0001, 0.001, 0.01, 0.1, 0.5) for different effect sizes (λ = 2, 2.5, 3, 3.5, 4, 4.5, 5, 5.5, 6). For all the effect sizes, the choices of gamma did not show big differences on the results. We have explained this to the main script and added the results below to the Additional file1: Fig. S7. Thank you for the comment.*

Reviewer 1 Minor Comment #3:

3. metaSKAT was proposed for meta-analysis, therefore, indeed it is designed for multiple studies. However, it should also be able to be applied when there is only a single study, with summary statistics for each SNP available instead of the genotypes. Any thought to compare your method to such a version of metaSKAT.

*Author Response:*

*Thank you for the comment and we compared MARS with MetaSKAT. The “SKAT statistic” that is derived from “score-statistic” and “weight” is used for MetaSKAT [4], which is not directly convertible to Z-scores that we have used for our simulated studies. Thus, alternatively, we generated phenotypes with a range of effect sizes β, and compared MARS with MetaSKAT along with SKAT and SKAT-O. We added the result to the Additional file 1: Fig. S1 as below.*

*Reference*

*1. Hormozdiari F, Kostem E, Kang EY, Pasaniuc B, Eskin E. Identifying causal variants at loci with multiple signals of association. Genetics. 2014;198:497–508.*

*2. Hormozdiari F, van de Bunt M, Segrè AV, Li X, Joo JWJ, Bilow M, et al. Colocalization of GWAS and eQTL Signals Detects Target Genes. Am J Hum Genet. 2016;99:1245–60.*

*3. Han B, Kang HM, Eskin E. Rapid and accurate multiple testing correction and power estimation for millions of correlated markers. PLoS Genet. 2009;5:e1000456.*

*4. Lee S, Teslovich TM, Boehnke M, Lin X. General framework for meta-analysis of rare variants in sequencing association studies. Am J Hum Genet. 2013;93:42–53.*
